# A finalized determinant for complete lignocellulose enzymatic saccharification potential to maximize bioethanol production in bioenergy *Miscanthus*

**DOI:** 10.1186/s13068-019-1437-4

**Published:** 2019-04-27

**Authors:** Aftab Alam, Ran Zhang, Peng Liu, Jiangfeng Huang, Yanting Wang, Zhen Hu, Meysam Madadi, Dan Sun, Ruofei Hu, Arthur J. Ragauskas, Yuanyuan Tu, Liangcai Peng

**Affiliations:** 1 Biomass and Bioenergy Research Centre, Huazhong Agricultural University, Wuhan, 430070 China; 2 College of Plant Science and Technology, Huazhong Agricultural University, Wuhan, 430070 China; 3 School of Materials and Chemical Engineering, Hubei University of Technology, Wuhan, 430068 China; 4 College of Food Science and Technology, Hubei University of Arts and Science, Xiangyang, 441053 China; 5 Department of Chemical and Biomolecular Engineering, University of Tennessee-Knoxville, Knoxville, TN 37996-2200 USA

**Keywords:** *Miscanthus*, Biomass saccharification, Bioethanol yield, Polymer features, Polymer linkages, Biomass porosity

## Abstract

**Background:**

*Miscanthus* is a leading bioenergy crop with enormous lignocellulose production potential for biofuels and chemicals. However, lignocellulose recalcitrance leads to biomass process difficulty for an efficient bioethanol production. Hence, it becomes essential to identify the integrative impact of lignocellulose recalcitrant factors on cellulose accessibility for biomass enzymatic hydrolysis. In this study, we analyzed four typical pairs of *Miscanthus* accessions that showed distinct cell wall compositions and sorted out three major factors that affected biomass saccharification for maximum bioethanol production.

**Results:**

Among the three optimal (i.e., liquid hot water, H_2_SO_4_ and NaOH) pretreatments performed, mild alkali pretreatment (4% NaOH at 50 °C) led to almost complete biomass saccharification when 1% Tween-80 was co-supplied into enzymatic hydrolysis in the desirable *Miscanthus* accessions. Consequently, the highest bioethanol yields were obtained at 19% (% dry matter) from yeast fermentation, with much higher sugar–ethanol conversion rates by 94–98%, compared to the other *Miscanthus* species subjected to stronger pretreatments as reported in previous studies. By comparison, three optimized pretreatments distinctively extracted wall polymers and specifically altered polymer features and inter-linkage styles, but the alkali pretreatment caused much increased biomass porosity than that of the other pretreatments. Based on integrative analyses, excellent equations were generated to precisely estimate hexoses and ethanol yields under various pretreatments and a hypothetical model was proposed to outline an integrative impact on biomass saccharification and bioethanol production subjective to a predominate factor (CR stain) of biomass porosity and four additional minor factors (DY stain, cellulose DP, hemicellulose X/A, lignin G-monomer).

**Conclusion:**

Using four pairs of *Miscanthus* samples with distinct cell wall composition and varied biomass saccharification, this study has determined three main factors of lignocellulose recalcitrance that could be significantly reduced for much-increased biomass porosity upon optimal pretreatments. It has also established a novel standard that should be applicable to judge any types of biomass process technology for high biofuel production in distinct lignocellulose substrates. Hence, this study provides a potential strategy for precise genetic modification of lignocellulose in all bioenergy crops.

**Electronic supplementary material:**

The online version of this article (10.1186/s13068-019-1437-4) contains supplementary material, which is available to authorized users.

## Background

Lignocellulose, an enormous renewable biomass resource, is now a well-established and valuable resource for cellulosic ethanol targeted as a partial replacement of fossil fuel energy with less net carbon release [1, 2]. In principle, cellulosic ethanol production involves three major steps: initial physical and chemical pretreatment to deconstruct plant cell walls, sequential enzymatic hydrolysis to release soluble sugars, and final yeast fermentation to produce bioethanol [3, 4]. However, the intrinsic recalcitrance of lignocellulose, which is evolutionally constructed to resist biotic attacks and abiotic stress, currently results in an unacceptably costly conversion process with potential secondary pollution to the environment [1, 5]. It hence becomes essential to explore optimal biomass process technology for efficient bioethanol production.

Lignocellulose recalcitrance is fundamentally determined by diverse cell wall compositions, specialized wall polymer features, and complicated wall-network structures [5, 6]. Cellulose is the major wall polymer of all lignocelluloses, but its crystallinity and degree of polymerization (DP) have been characterized as one of the factors negatively affecting biomass enzymatic saccharification under various physical and chemical pretreatments [3, 7]. By comparison, xylans are the major hemicelluloses in grass plants, and its arabinose substitution degree (reverse xylose/arabinose ratio, X/A) could positively affect biomass enzymatic hydrolysis by reducing cellulose crystallinity [8, 9]. Lignin is an amorphous wall polymer composed of three monomers: *p*-hydroxyphenyl (H), guaiacyl (G), and syringyl (S) [6, 10]. Lignin and hemicelluloses encapsulate cellulose microfibrils thereby restricting cellulose accessibility and providing plant rigidity and mechanical support which promote cell wall recalcitrance. Recent reports have indicated that lignin may play dual roles in biomass enzymatic hydrolysis due to three monomer distinct proportions [5, 10]. Because lignocellulose is a porous medium, biomass porosity has been regarded as a general factor directly accounting for biomass enzymatic hydrolysis [11–13], but much is yet unknown about how biomass porosity is characteristically affected by wall polymer features and wall-network styles. Notably, as biomass porosity has multiple parameters measured from different assays such as Simons’ stain [11, 14], Congo red [15], and nitrogen adsorptions [3], it remains yet to be identified the crucial parameter(s) precisely accounting for biomass saccharification and bioethanol production.

To overcome lignocellulose recalcitrance, many physical and chemical pretreatments have been performed in various biomass residues by partially removing lignin and hemicelluloses, distinctively altering wall polymer features and largely increasing biomass porosity [6, 11, 16, 17]. For instances, acid (H_2_SO_4_) and alkali (NaOH) agents have been broadly used for chemical pretreatments, whereas liquid hot water (LHW) is a practicable physicochemical pretreatment [12, 18]. Importantly, attempts have been made to find out the optimal pretreatment conditions for a complete biomass enzymatic hydrolysis by evaluating different pretreatment conditions such as chemical concentrations, treatment temperatures, incubation times, and substrate dosages [1, 17, 19, 20]. However, due to diverse cell wall compositions and complicated wall structures [5], different pretreatments distinctively extract different wall polymers and typically alter wall polymer features and inter-linkages [10, 12], leading to difficulties in sorting out a universal standard to judge optimal pretreatment. In addition, surfactants, such as Tween and PEG, have been co-supplied to enhance enzymatic hydrolysis of pretreated biomass residues by lessening cellulase adsorption with lignin and hemicelluloses [3, 21, 22].

Among the fast growing C4 perennial grasses, *Miscanthus* is a leading bioenergy crop due to much high biomass yield, low nitrogen input, and less water and energy requirements. Native *Miscanthus* genus consists of about 20 species with more than 1000 germplasm accessions, leading to wide-ranging ecological adaptability and divergent biomass resources [23, 24]. Although physical and chemical pretreatments have been conducted on biomass residues of *Miscanthus*, it remains to be determined the optimal technology for higher bioethanol production. Based on diverse cell wall compositions and relatedly varied enzymatic saccharification among large population of *Miscanthus* accessions examined in our previous studies [8, 10, 18, 25–27], we initially took advantage of these studies to select four representative pairs of *Miscanthus* samples, and then performed LHW and chemical pretreatments under various conditions. In terms of the optimal pretreatments, this study detected much-enhanced biomass saccharification and highest bioethanol yield compared to the previously reported ones [10, 28–31]. Furthermore, this study examined the changes of biomass porosity for the accessibility of lignocellulosic substrates at the expense of wall polymer extraction, and detected the alterations of major polymer features for understanding of how biomass porosity could be largely increased under optimal pretreatment. Notably, based on the integrative analyses, this work at the first time sorted out the applicability of equations to precisely account for biomass saccharification and bioethanol production, leading to raising a novel standard that should be applicable to judge any types of biomass process technology for maximum biofuels production in all bioenergy crops.

## Results and discussion

### Optimal pretreatments for enhancing biomass enzymatic saccharification

Among hundreds of *Miscanthus* accessions examined previously in our laboratory [8, 10, 11, 18, 23, 25, 26], this study selected four typical pairs of *Miscanthus* accessions that showed distinct cell wall compositions including cellulose, hemicelluloses, and lignin (Table 1). In comparison, Pair I samples only showed significantly different cellulose levels at *P *< 0.05 level (*n* = 3), Pair II had varied lignin contents at *P *< 0.01, and Pair III exhibited a significant difference in hemicellulose levels at *P *< 0.01, indicating that those samples could be applicable to examine each wall polymer distinctive impact on biomass enzymatic saccharification. Notably, Pair IV samples did not show significantly different cell wall compositions at *P *> 0.05, suggesting that this pair of samples should be powerful to explore how biomass enzymatic hydrolysis is affected by wall polymer features and interlink styles.

**Table 1:** Cell wall composition of four typical pairs of *Miscanthus* accessions (% dry matter)

Pair	Sample	Cellulose	Hemicelluloses	Lignin
I	Msi69 (H)	*37.2* (± *0.58*)*	30.9 (± 0.72)	21.9 (± 0.18)
	Mlu01 (L)	*44.3* (± *1.34*)	30.3 (± 0.39)	21.7 (± 1.49)
II	Mlu26 (H)	44.4 (± 0.76)	29.1 (± 0.78)	*20.4* (± *0.03*)**
	Msa01 (L)	44.1 (± 1.45)	29.4 (± 0.37)	*22.7* (± *0.34*)
III	Mlu11 (H)	39.5 (± 0.93)**	*30.5* (± *0.33*)**	22.0 (± 0.33)
	Mfl17 (L)	37.1 (± 0.85)	*27.4* (± *0.34*)	21.5 (± 0.15)
IV	Mlu02 (H)	39.7 (± 0.18)	29.0 (± 0.30)	20.2 (± 0.56)
	Mfl13 (L)	39.3 (± 0.95)	29.5 (± 0.15)	19.2 (± 0.08)

*^,^ ** as significant difference between two samples of each pair by t-test at P < 0.05 and 0.01 (values italicized) (n = 3); (H) and (L) represented two samples of each pair showing relatively high (H) and low (L) biomass saccharification. The data as means ± SD.

**Fig. 1:**
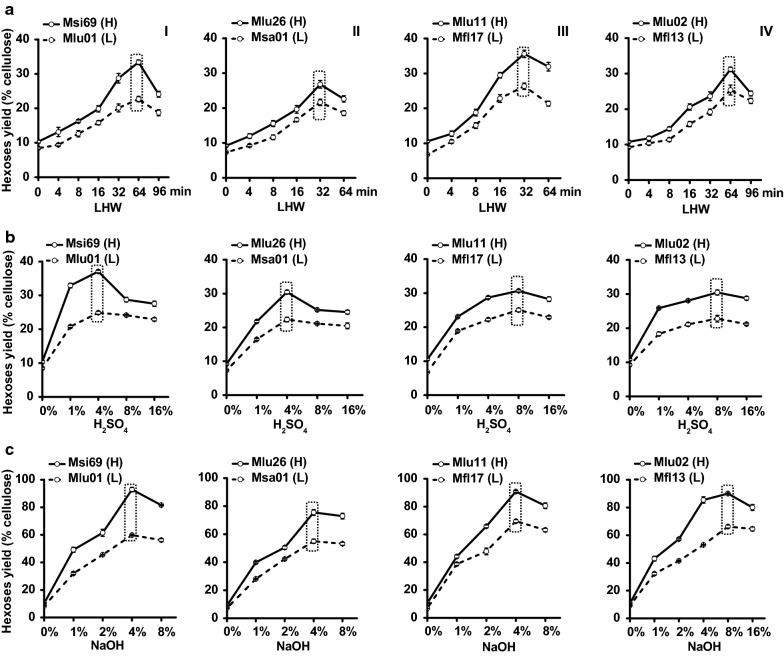
Biomass saccharification under liquid hot water and chemical pretreatments in four typical pairs of *Miscanthus* accessions. **a** Hexose yields (% cellulose) released from enzymatic hydrolysis after LHW pretreatments under a time course; **b**, **c** hexose yields from enzymatic hydrolysis after H_2_SO_4_, and NaOH at a series of concentrations. (H) and (L) represented two samples of each pair showing relatively high (H) and low (L) biomass saccharification, and the data as mean ± SD (*n* = 3)

Biomass saccharification (digestibility) has been well defined by measuring the hexoses yield (% cellulose) released from enzymatic hydrolysis of the pretreated lignocellulose [9, 20, 32, 33]. Using previously established approaches [21, 23, 25, 27], this study performed a series of LHW, H_2_SO_4_, and NaOH pretreatments to achieve highest biomass enzymatic saccharification (Fig. 1). For LHW pretreatments at fixed temperature (200 °C), a time course was conducted in all four pairs of *Miscanthus* samples (Fig. 1a). By comparison, Pairs I, IV samples, respectively, showed the highest hexose yields (23–33%) released from enzymatic hydrolysis after 64-min LHW pretreatments, whereas Pairs II, III had the highest values (22–36%) after 32 min LHW pretreatments (Additional file 1: Table S1). Notably, two *Miscanthus* samples of each pair showed significantly different hexose yields under the LHW pretreatments, which was attributed to their distinct cell wall compositions (Pairs I, II, III) and wall polymer features (Pair IV).

Because chemical (H_2_SO_4_, NaOH) pretreatments at high temperatures could increase inhibitor formation or cause sugar oxidation [10, 18, 26], this study focused on using a series of acid and alkali concentrations under fixed temperatures (Fig. 1). In terms of acid pretreatments, Pairs I, II samples showed the highest hexose yields under pretreatments of 4% H_2_SO_4_ at 121 °C for 20 min, whereas 8% H_2_SO_4_ pretreatments are optimal for Pairs III, IV (Fig. 1b; Additional file 1: Table S2). However, both optimal LHW and acid pretreatments led to the relatively low hexose yields at less than 40% (% cellulose). By comparison, optimal alkali pretreatments (4% NaOH at Pairs I, II, III; 8% NaOH at Pair IV at 50 °C for 2 h) yielded much higher hexose yields ranging from 55 to 93% (Fig. 1c; Additional file 1: Table S3), consistent with the previous reports that the alkali pretreatments lead to much higher biomass enzymatic saccharification than those of the acid pretreatments [10, 18, 26, 34]. Although the three optimal pretreatment conditions varied for the four pairs of *Miscanthus* accessions such as pretreatment time and chemical concentration, only Pair II (Mlu26, Mas01) samples consistently required relatively less pretreatment time (32 min) of LHW and low concentrations of acid (4% H_2_SO_4_) and alkali (4% NaOH) to achieve highest biomass saccharification. In addition, as shown in Fig. 1, the samples defined as H of the four pairs consistently yielded higher hexose yields than those of their paired samples defined as L in all pretreatments performed in this study, indicating that lignocellulosic properties fundamentally determine biomass enzymatic saccharification, regardless of the different pretreatments examined [3, 7, 11].

**Fig. 2:**
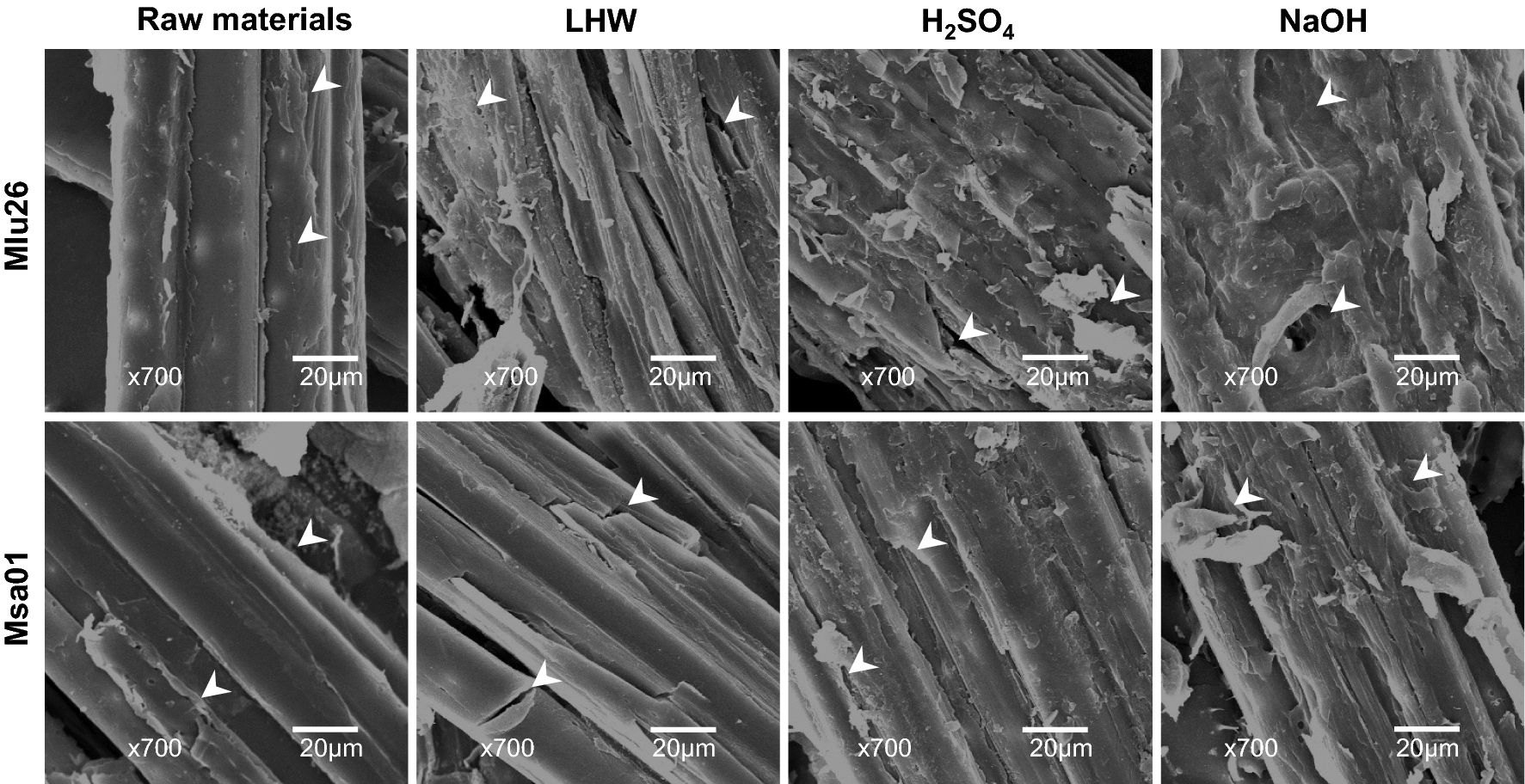
Scanning electron microscopic observations of lignocellulose residues after optimal LHW and chemical (4% H_2_SO_4_, 4% NaOH) pretreatments and sequential enzymatic hydrolysis in the representative Pair II samples. Arrows indicated rough points of lignocellulose surfaces

Furthermore, we observed the morphology of the pretreated biomass residues in the Pair II samples under scanning electron microscopy (Fig. 2). As compared to the raw materials, the biomass residues obtained from the three optimal pretreatments and enzymatic hydrolysis exhibited much rougher surfaces as indicated by arrows, in particular from the NaOH pretreatments, consistent with the previous reports in *Miscanthus* and other grass plants [25, 26, 35, 36]. Notably, the Mlu26 sample of Pair II showed rougher faces than those of its paired sample (Msa01), consistent with its relatively high biomass enzymatic saccharification properties.

### Tween-80 co-supply for almost complete biomass enzymatic hydrolysis

**Fig. 3:**
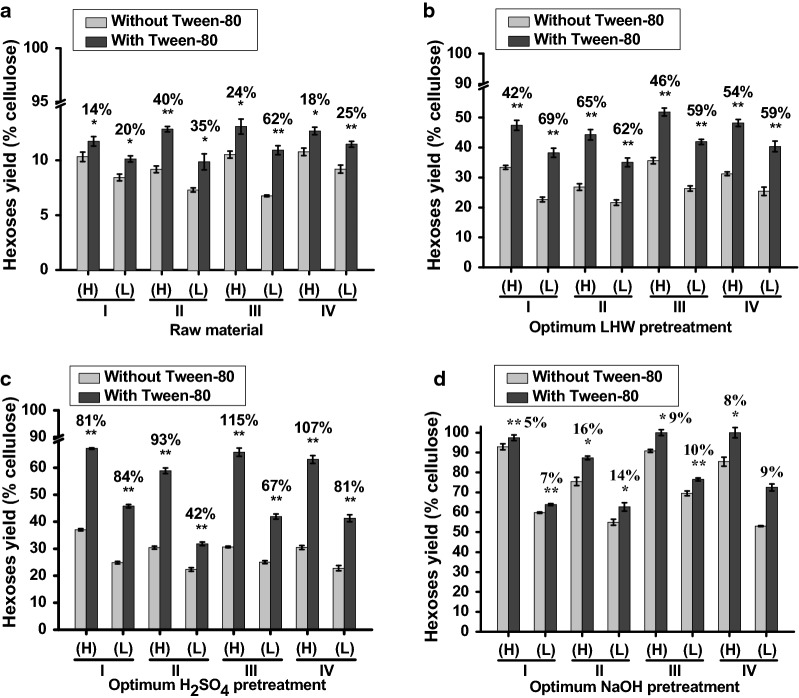
Tween-80 enhancement on biomass enzymatic saccharification under optimal pretreatments in four typical pairs of *Miscanthus* accessions. **a** Hexose yields (% cellulose) released from enzymatic hydrolysis of the raw materials (without pretreatment) co-supplied with 1% Tween-80. **b**–**d** Hexose yields released from enzymatic hydrolysis co-supplied with 1% Tween-80 after optimal LHW, H_2_SO_4_, and NaOH pretreatments. (H) and (L) represented two samples of each pair showing relatively high (H) and low (L) biomass saccharification, and the data as mean ± SD (*n* = 3). * and ** as significant difference between the control (without Tween-80) and the sample co-supplied with Tween-80 by *t* test at *P *< 0.05 and 0.01, respectively, and the percentage (%) calculated by subtraction of two samples divided by the value of control (without Tween-80)

It has been reported that co-supplement with Tween-80 could enhance enzymatic hydrolysis of the pretreated biomass residues in grass plants [3, 21, 37]. While 1% Tween-80 was co-supplied in this study, all four pairs of samples showed significantly increased hexose rates by 14–62% from enzymatic hydrolysis of the raw materials (without pretreatments), compared to the controls/without Tween-80 (Fig. 3a; Additional file 1: Table S4). By comparison, the Tween-80 co-supply led to significantly enhanced rates of hexose yields by 42–69% from the optimal LHW pretreatments, whereas it caused an increase of 42–115% for the optimal H_2_SO_4_ pretreatments (Fig. 3b, c; Additional file 1: Table S4). Notably, although the Tween-80 supplement could lead to the increased hexose rates by 5–14% in the optimal NaOH pretreatments (Fig. 3d), two H samples (Mlu11, Mlu02) of Pair III, IV both showed almost complete biomass enzymatic hydrolysis with hexose yields close to 100% (% cellulose) and other two H samples (Msi69, Mlu26,) of Pair I, II also had much higher hexose yields at 97% and 87%, as compared to the optimal LHW and H_2_SO_4_ pretreatments (Additional file 1: Table S4). However, the Mlu26 sample of Pair II showed slightly fewer hexoses yield for the optimal NaOH pretreatment, probably due to its relatively higher cellulose level than those of the other samples (Table 1). Therefore, the co-supply with 1% Tween-80 was effective for enhanced biomass enzymatic saccharification in both raw materials and pretreated biomass residues, consistent with previous findings in the steam-exploded biomass residues [11, 21, 37]. In addition, as Tween-80 is powerful for lessening cellulase adsorption with lignin, much more enhanced enzymatic hydrolysis from 1% Tween-80 co-supply in the optimal LHW and H_2_SO_4_ pretreatments was attributed to relatively high lignin levels in the pretreated biomass residues, compared to the optimal NaOH-pretreated residues as described below (Additional file 1: Table S7).

### Optimal pretreatments for the highest bioethanol production

**Fig. 4:**
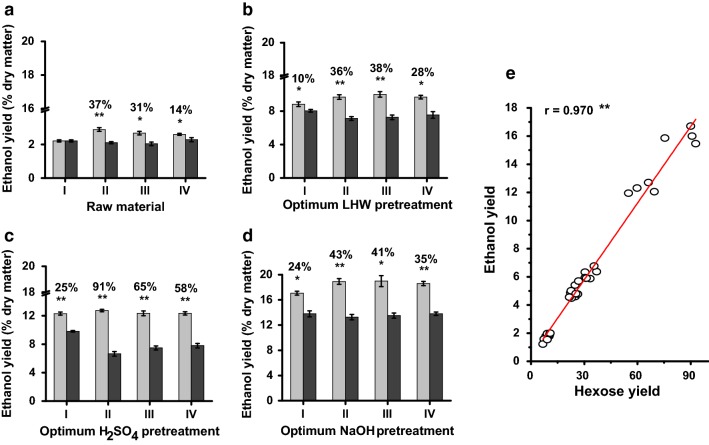
Bioethanol production released from yeast fermentation in four typical pairs of *Miscanthus* accessions. **a** Ethanol yield (% dry matter) using total hexoses released from enzymatic hydrolysis of raw materials co-supplied with 1% Tween-80. **b**–**d** Ethanol yields using total hexoses released from enzymatic hydrolysis of three optimal pretreatments co-supplied with 1% Tween-80. **e** Correlation analysis between hexoses and ethanol yields from all pretreated biomass residues and raw materials (*n* = 32). Gray and black column indicated two samples of each pair showing relatively high (H) and low (L) biomass saccharification as shown in Fig. 3, and the data as mean ± SD (*n* = 3). * and ** as significant difference between two samples of each pair by *t* test at *P *< 0.05 and 0.01 (*n* = 3), and the percentage (%) calculated by subtraction of two samples divided by relative low value

Using total hexoses released from enzymatic hydrolysis of the pretreated biomass residues co-supplied with 1% Tween-80, this study performed well-established yeast fermentation to obtain bioethanol production [3, 20, 21, 36, 37]. Without pretreatment, the four pairs of samples exhibited low bioethanol yields at 2.0–2.9% (% dry matter) using total hexoses released from direct enzymatic hydrolysis of the raw materials (Fig. 4a; Additional file 1: Table S5). By comparison, the optimal LHW pretreatments led to the bioethanol yields of 7.1–10.0% (% dry matter) in all samples, whereas the optimal H_2_SO_4_ pretreatments had the ethanol yields of 6.7–12.7% (Fig. 4b, c; Additional file 1: Table S5). Notably, due to much higher hexose yields obtained from optimal NaOH pretreatments (Additional file 1: Table S4), three H samples (Mlu26, Mlu11, Mlu02) of Pairs II, III, IV had the highest bioethanol yields at 19% (Fig. 4d; Additional file 1: Table S5), compared to the previous reports (12–18%) of the *Miscanthus* process subjected to relatively strong pretreatments (Table 2). However, the L samples of each pairs had the bioethanol yields at 13–14% from the optimal NaOH pretreatments, consistent with their relatively low hexose yields (Additional file 1: Table S4). Meanwhile, correlation analysis indicated that the bioethanol yields were positively correlated with the hexose yields from three optimal pretreatments at *P *< 0.01 level (*n* = 32) with much high *r* value at 0.97 (Fig. 4e), consistent with the findings that all samples had much high sugar–ethanol conversion rates (Additional file 1: Table S6). It also suggests that three optimal pretreatments may release little inhibitors to yeast fermentation. Hence, the high biomass saccharification and bioethanol production in the desirable samples of each pair should be mainly due to their distinct cell wall compositions and characteristic wall polymer features.

**Table 2:** Comparison of bioethanol yields obtained in this study and the previous studies in different *Miscanthus* accessions

Sample	Pretreatment	Ethanol yield (% dry matter)	Sugar–ethanol conversion rate (%)	References
Mlu26	4% NaOH (50 °C, 2 h) + 1% Tween-80	19%	98	This study
Mlu11	4% NaOH (50 °C, 2 h) + 1% Tween-80	19%	94	
	10% NaOH (90 °C, 1 h)	12%	78	[31]
	4% NaOH (50 °C, 2 h)	13%	78	[10]
	15% Aqua. NH_3_ (60 °C, 24 h) + EBI	16%	97	[30]
	2% NaOH (140 °C, 8 min)	17%	85	[28]
	1.6% NaOH (95 °C)	18%	66	[29]

### Large wall polymer extraction and hydrolysis for efficient energy conversion

**Fig. 5:**
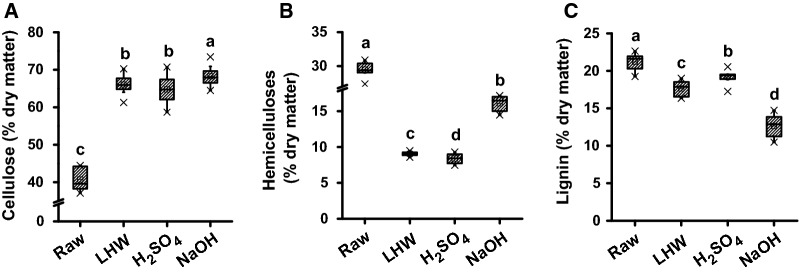
Alteration of cell wall composition after three optimal pretreatments in four typical pairs of *Miscanthus* accessions. **A** Cellulose level (% dry matter); **B** hemicelluloses and **C** lignin. Raw as raw materials without pretreatment. The line and square within the box presented the median and mean values of all data (*n* = 32); the bottom and top edges of the box indicated 25% and 75% of all data; the top and bottom bars (×) presented maximum and minimum values of all data, and the different letters (a, b, c, d) indicated that the mean values are significantly different from each other by LSD test (*P *< 0.05), respectively

To understand pretreatment enhancements of biomass saccharification and bioethanol production, this study examined major wall polymer extractions from three optimal pretreatments in four pairs of *Miscanthus* samples (Fig. 5; Additional file 1: Table S7). Compared to the controls (raw materials), three optimal pretreatments, respectively, led to increased cellulose levels by 62%, 59%, and 68% (average of four pairs), but the optimal LHW and H_2_SO_4_ pretreatments showed similar cellulose levels at *P *> 0.05 (Fig. 5A). By contrast, all biomass samples had significantly reduced hemicellulose contents from three optimal pretreatments at *P *< 0.01 levels, compared to the raw materials (Additional file 1: Table S7). However, both LHW and H_2_SO_4_ pretreatments led to much more hemicellulose extractions than those of the NaOH pretreatment (Fig. 5B). Notably, the NaOH pretreatment could largely extract lignin (Fig. 5C), which should be the partial cause for more enhanced biomass enzymatic saccharification achieved from the NaOH pretreatments, because lignin removal could provide cellulose microfibril accessibility and also reduce adsorption with cellulase enzymes [10, 18, 21]. Meanwhile, the extractions of both hemicelluloses and lignin resulted in relatively increased cellulose levels in the pretreated residues. Taken together, the results suggest that the three optimal pretreatments could distinctively extract hemicellulose and lignin, consistent with the previous reports in *Miscanthus* and other C4 grasses [11, 21, 33]. It also suggests that LHW or acid pretreatment at high temperature may mainly extract hemicelluloses by splitting chemical bonds, whereas the alkali pretreatment may largely solubilize ferulate cross-linked hemicelluloses and lignin by dissociating hydrogen bonds with cellulose microfibrils [10, 21, 38].

**Fig. 6:**
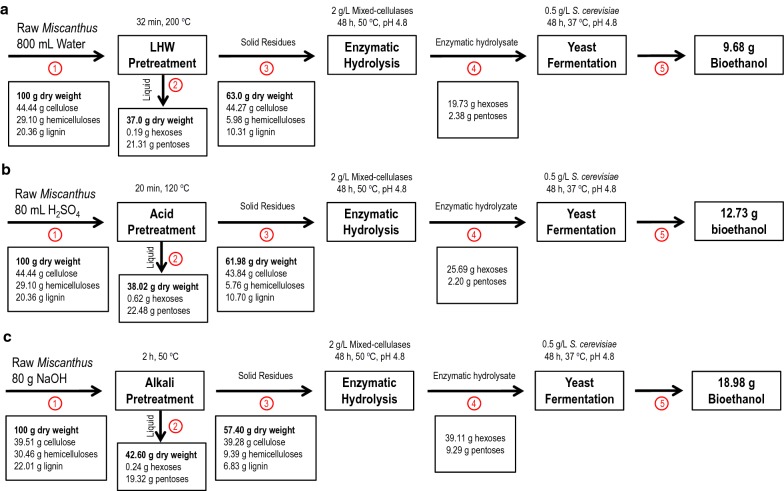
Mass balance flow chart of *Miscanthus* biomass for bioethanol (on a 100 g basis) among three optimal pretreatments, simultaneous saccharification, and fermentation process. **a** LHW pretreatment of Mlu26 *Miscanthus* sample; **b** H_2_SO_4_ (purity: 98%) pretreatment of Mlu26 *Miscanthus* sample; **c** NaOH pretreatment of Mlu11 *Miscanthus* sample. Mass amount of insoluble wall polymers (cellulose, hemicelluloses, and lignin) are presented in streams 1 and 3. Mass amount of soluble sugars (hexoses and pentoses) and ethanol yield are presented in streams 2, 4, and 5

Furthermore, this study investigated the overall mass balance among the three optimal pretreatments, subsequent enzymatic hydrolysis, and yeast fermentation (Table 3; Fig. 6). The resultant composition of the products was normalized to a 100 g dried raw material of *Miscanthus* and the solid recovery after each pretreatment was calculated as the mass of oven-dried pretreated residues. Under the three optimal pretreatments, the cellulose-rich residues were recovered at 53–69%, but the highest ethanol yield (18.98 g) could be produced from 100 g of the biomass pretreated with NaOH at 50 °C for 2 h, indicating an efficient energy conversion from the *Miscanthus* biomass pretreated with mild alkali pretreatment.

**Table 3:** Overall mass balance for the optimal (LHW, H_2_SO_4_, NaOH) pretreatments and SSF process of *Miscanthus* to bioethanol

	Raw material	Optimal pretreatments		
		LHW^a^	H_2_SO_4_	NaOH
Solid recovery after optimal pretreatments	100	56.3‒68.9 (± 4.55)	58.9‒64.9 (± 2.07)	53.1‒67.7 (± 5.14)
Cellulose content in pretreated residues	37.1‒44.4 (± 3.13)	37.1‒44.3 (± 3.09)	36.4‒43.8 (± 3.07)	36.3‒43.9 (± 3.07)
Hemicellulose in pretreated residues	27.4‒30.9 (± 1.10)	4.8‒6.4 (± 0.52)	4.6‒5.9 (± 0.55)	7.7‒11.4 (± 1.34)
Lignin in pretreated residues	19.2‒22.6 (± 1.14)	9.7‒12.9 (± 1.10)	10.7‒13.3 (± 0.82)	5.6‒9.0 (± 1.21)
Glucose produced by enzymatic hydrolysis	4.0‒5.7 (± 0.59)	15.5‒20.3 (± 2.03)	13.9‒25.7 (± 5.03)	28.2‒39.1 (± 5.36)
Ethanol produced by yeast fermentation^b^	2.0‒2.9 (± 0.31)	7.1‒10.0 (± 1.18)	6.7‒12.7 (± 2.56)	13.3‒19.0 (± 2.64)

All the data are reported as grams per 100 g of initial *Miscanthus* biomass

^a^The ranges are the results of four pairs of *Miscanthus* samples with standard deviation in brackets (± SD)

^b^The data are the results of 48-h simultaneous saccharification and fermentation by 2 g/L mixed-cellulases and 0.5 g/L *S. cerevisiae*

### Distinct alterations of wall polymer features from optimal pretreatments

**Fig. 7:**
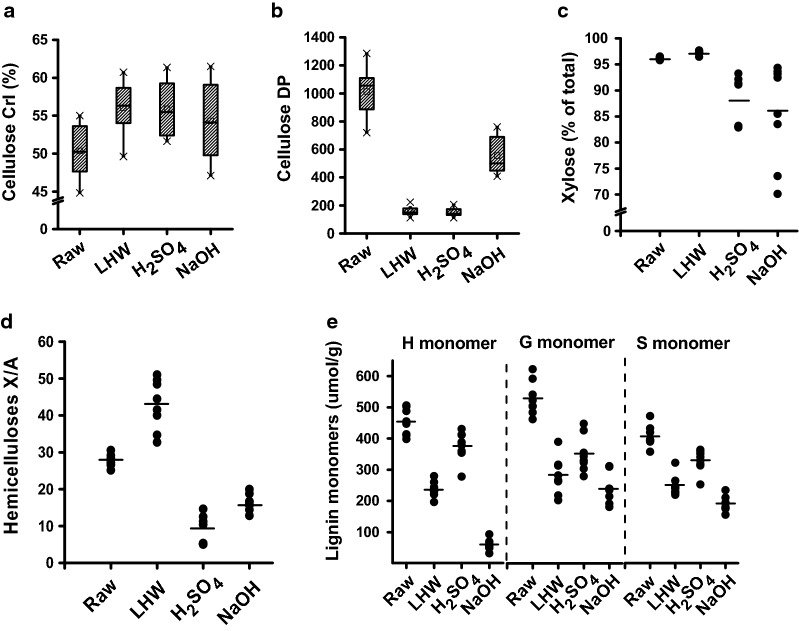
Alteration of major wall polymer features after three optimal pretreatments in four typical pairs of *Miscanthus* accessions. **a** Cellulose CrI (%); **b** cellulose DP; **c** xylose of hemicelluloses (% of total); **d** X/A ratio of hemicelluloses, and **e** three lignin monomers (µmol/g). Raw as raw materials without pretreatment. The line and square within the box presented the median and mean values of all data (*n* = 32); the bottom and top edges of the box indicated 25 and 75 percentiles of all data; the top and bottom bars (×) presented maximum and minimum values of all data, and the different letters (a, b, c, d) indicated that the mean values are significantly different from each other by LSD test, respectively

As wall polymer features could distinctively affect biomass enzymatic saccharification under physical and chemical pretreatments [6–9, 19, 32], this study determined major wall polymer features of biomass samples after three optimal pretreatments (Fig. 7). Compared with the controls (starting materials), the three optimal pretreatments led to slightly increased cellulose crystalline index (CrI) values in all biomass residues (Fig. 7a; Additional file 1: Table S8), consistent with the previous reports that hemicellulose and lignin extractions could raise cellulose crystallinity due to disassociation of hydrogen bonds among wall polymers [11, 18, 19, 39]. Meanwhile, three optimal pretreatments largely reduced cellulose degree of polymerization (DP) by 2–8 fold in all pretreated biomass residues, compared to the raw materials (Fig. 7b, Additional file 1: Table S8). Because cellulose DP has been reported to negatively affect biomass enzymatic hydrolysis [3, 7, 32], the reduced cellulose DP should be the factor accounting for enhanced biomass enzymatic saccharification. Furthermore, this study determined hemicellulosic monosaccharides composition and characterized two major monosaccharide ratio (xylose/arabinose, X/A), which has been characterized to be a negative factor on biomass enzymatic saccharification [8, 9]. Despite of the LHW-pretreated samples containing similar xylose levels to the raw materials, they showed much higher X/A ratios (Fig. 7c, d; Additional file 1: Table S9), suggesting that the LHW optimal pretreatments should extract relatively more branched arabinose. By contrast, two chemical (H_2_SO_4_, NaOH)-pretreated samples showed relatively lower xylose and higher arabinose contents, leading to much reduced X/A ratios, which should be another cause for enhanced biomass enzymatic saccharification from both optimal chemical pretreatments. In addition, all pretreated biomass samples exhibited much reduced monomers (H, G, S) of lignin, compared to the raw materials, but the three monomer proportions (H/G, H/S, S/G) were variable from 0.24 to 1.07, 0.31 to 1.14, and 0.76 to 0.93 (Fig. 7e; Additional file 1: Table S10), indicating that three optimal pretreatments may rupture hemicellulose–lignin complexes. In particular, as the alkali pretreatment could effectively cleave ferulate and *p*-coumarate cross-linkages [6, 10, 38], it may explain why high biomass saccharification was achieved from the optimal NaOH pretreatment compared to the other two optimal pretreatments performed in this study.

### Characteristic changes of wall polymer linkages from optimal pretreatments

**Fig. 8:**
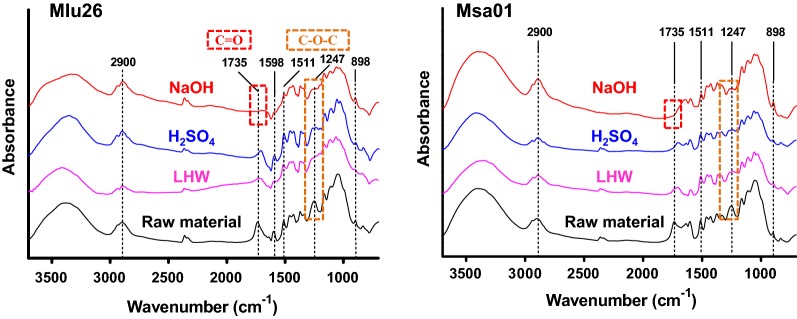
Comparison of Fourier transform infrared spectroscopic profiling among the raw materials (black) and three optimal pretreated (LHW-pink, 4% H_2_SO_4_-blue, 4% NaOH-red) biomass residues of Pair II *Miscanthus* samples. Dot squares indicated the majorly altered bonds from the optimal pretreatments as elucidated in Additional file 1: Table S11

Since three optimal pretreatments distinctively extracted wall polymers and altered wall polymer features, this study further applied Fourier transform infrared (FTIR) spectroscopy to detect potential alterations of wall polymer linkages in the pretreated biomass residues using the representative Pair II samples (Fig. 8; Additional file 1: Table S11). Despite the pretreated biomass residues and raw materials presented a similarity of most peaks examined, the apparent variations in characteristic peaks were observed in the regions of 1735–1247 cm^−1^ (Fig. 8). For instance, the absorption bands located at 1735 cm^−1^ were seen as either ester-linked acetyl and uronic groups of the hemicelluloses or carboxylic acid groups of ferulic and *p*-coumaric acids of lignin and hemicelluloses (Additional file 1: Table S11) [38]. However, as compared to the raw materials, the intensities of those bands were decreased in the optimal LHW- and H_2_SO_4_-pretreated biomass residues, and the peaks even disappeared in the NaOH-pretreated residues, suggesting an almost complete removal of hemicelluloses–lignin linkages from the optimal NaOH pretreatments. The absorptions at 1598 and 1511 cm^−1^ were attributed to C=C stretching vibration and aromatic skeleton C=C stretching in lignin [22, 40]; these two peaks declined after the LHW and H_2_SO_4_ pretreatments and were almost absent from the NaOH pretreatments. These results indicate that the chemical bonds associated with aromatic ring structures of lignin were removed from the optimal pretreatments, in particular from the NaOH pretreatments. The absorption peaks at 1247 cm^−1^, which were related to the aryl–alkyl ether bonds in lignin, showed diminished intensities in the pretreated residues as compared to the raw materials, confirming that the lignin removal occurred from three optimal pretreatments. Hence, the results demonstrated a significant extraction of hemicellulose–lignin complexes from the optimal pretreatments. It also confirmed more effective extraction of wall polymers from the optimal NaOH pretreatments, consistent with its higher biomass enzymatic saccharification properties.

### A significant increase of biomass porosity under optimal pretreatments

**Fig. 9:**
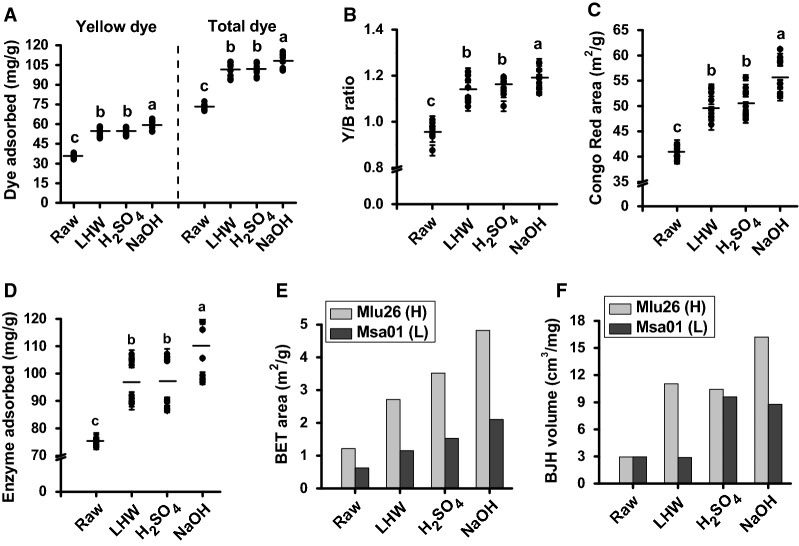
Comparison of biomass porosity among the raw materials (raw) and three optimal pretreated (LHW, H_2_SO_4_, NaOH) biomass residues of *Miscanthus* samples. **A** Yellow dye (DY) and total dye from Simons stains in four typical pairs of samples (mg/g). **B** Ratio (*Y*/*B*) between yellow dye (DY) and blue dye (DB) from Simons stains in four typical pairs of samples. **C** Congo red (CR) dye stain in four typical pairs of samples (m^2^/g; 1 g CR adsorbed corresponds to the area 1055 m^2^ of biomass). **D** Mixed-cellulase enzyme adsorption (*E*_max_; mg/g biomass) in four typical pairs of samples. **E** BET specific surface area and **F** BJH cumulative pore volume in the Pair II samples. The line among the solid dots as means (*n* = 8) and the different letters (a, b, c, d) indicated that the mean values are significantly different from each other by LSD test, respectively

It has been reported that wall polymer extraction could improve biomass porosity facilitating increased cellulase loading and accessibility to cellulose surface [3, 12, 13]. Because biomass porosity is an integrative and complicated parameter, this study performed multiple assessments including Simons’ stain (SS), Congo red (CR) dye adsorption, cellulase enzyme adsorption, and N_2_ adsorption (Fig. 9; Additional file 1: Table S12). Using our recently improved SS method that is tailored for high binding affinity with hydroxyl groups of cellulose and other wall polymers [11], we observed significantly increased yellow dye (DY) at *P *< 0.05 or 0.01 level in all four pairs of biomass samples from three optimal pretreatments, compared to the raw materials (Fig. 9A; Additional file 1: Table S12). Despite that the blue dye (DB) values were variable among the three optimal pretreatments, all pretreated biomass samples had much higher total dye values (DY + DB) and DY/DB (Y/B) ratios than those of the raw materials (Fig. 9A, B; Additional file 1: Table S12). Notably, the optimal NaOH pretreatments provided higher DY and total dye values, as well as Y/B ratios, compared to the optimal LHW and H_2_SO_4_ pretreatments in all biomass samples, which supports the findings of higher biomass enzymatic saccharification from the optimal NaOH pretreatments. In addition, because DY and DB are, respectively, accountable for relatively large and small pore sizes of biomass residues [11, 41], the results indicated that relatively large accessible surface areas of biomass residues could be generated from three optimal pretreatments, in particular from the optimal NaOH pretreatment. It also suggested that the small pore size of DB stain should not be suitable for cellulase enzyme accession and loading [11, 41].

Furthermore, the Congo red (CR) was applied to measure the specific surface area of cellulose [15, 42, 43]. In general, three optimal pretreatments led to significantly increased CR values in all biomass samples at *P *< 0.05 or 0.01 level, compared to the raw materials (Fig. 9C; Additional file 1: Table S12). In particular, the NaOH pretreatments even had higher CR values than those of the LHW and H_2_SO_4_ pretreatments, consistent with the SS assay. To confirm the CR assay, this study also characterized the adsorption behavior of total amount of mixed-cellulase enzymes at 4 °C in the pretreated biomass residues, which has been used to determine biomass enzymatic saccharification as shown in Fig. 1. In comparison, the average enzyme (mixed-cellulase) adsorption capacity of all raw materials was 75 mg/g, but was, respectively, increased by 30%, 29%, and 46% in the LHW-, H_2_SO_4_-, and NaOH-pretreated biomass residues (Fig. 9D; Additional file 1: Table S12), indicating that three optimal pretreatments should either produce more surface areas for enzyme contact with cellulose and other wall polymers or increase pore sizes for mixed-cellulase loading. Consistently, the optimal NaOH pretreatment provided much higher enzyme adsorption capacities than those of the optimal LHW and H_2_SO_4_ pretreatments, re-confirming the high effectiveness of alkali pretreatment for cellulose accessibility to cellulases during enzymatic hydrolysis. To re-verify that the optimal pretreatments could increase biomass porosity as measured by SS, CR, and enzyme adsorption, this study also used the nitrogen porosimetry method to determine Brunauer–Emmett–Teller (BET) surface area and Barrett, Joyner, and Halenda (BJH) pore volume in the representative Pair (II) of *Miscanthus* samples (Mlu26; Msa01). Compared to the controls (raw materials), both samples of Pair II showed similar increase trends of BET area or BJH pore volume from three optimal pretreatments, with the highest surface areas at 4.82 and 2.1 m^2^/g and pore volume 16.22 and 8.78 cm^3^/mg in the optimal NaOH-pretreated biomass residues (Fig. 9E, F; Additional file 1: Table S13), consistent with the findings of high values of SS, CR, and enzyme adsorption from the NaOH pretreatments.

Therefore, all H samples (relatively high saccharification) showed much higher biomass porosity than that of the L samples (low saccharification) from three optimal pretreatments, including SS, CR, enzyme adsorption, and nitrogen porosimetry assays, suggesting that biomass porosity could be an excellent parameter accounting for biomass enzymatic saccharification properties in *Miscanthus* [3, 11, 12, 17].

### A key factor of biomass porosity on biomass enzymatic saccharification

Correlation analysis has been broadly applied to explore significant lignocellulose impacts on biomass enzymatic saccharification under various physical and chemical pretreatments [7, 12, 36, 39, 44]. In this study, we initially performed a Spearman correlation analysis between biomass porosity and hexose yields released from enzymatic hydrolysis after three optimal pretreatments of all *Miscanthus* samples, as well as the bioethanol yields generated from the final yeast fermentation. Significantly, all four assays of biomass porosity showed a positive correlation with hexose yields or bioethanol yields at *P *< 0.01 levels (*n* = 32), with extremely high coefficient values (Additional file 1: Table S14), indicating that biomass porosity should be a powerful parameter accountable for biomass enzymatic saccharification under various pretreatments [11–13]. It also suggests that those four pairs of *Miscanthus* samples were sufficient for a statistical analysis. Meanwhile, correlation analysis was conducted with three wall polymer levels and their significant features (Additional file 1: Table S15). A significantly negative correlation was found between hexose yields and significant wall polymer levels and features including cellulose DP, two major hemicellulose monosaccharides and X/A ratio, total lignin level, and three monomers (H, G, S) proportions. Because two lignin monomer ratios (H/G, H/S) showed much lower coefficient values than those of three monomer proportions (Additional file 1: Table S15), this study then focused on investigating three lignin monomer impacts on biomass enzymatic saccharification. Despite that cellulose CrI of raw materials has been well examined as the negative factor on biomass enzymatic saccharification [3, 7, 9, 36], this study indicated that the CrI of three optimal pretreated biomass residues did not show significant correlation with biomass enzymatic hydrolysis, probably due to the distinct hemicellulose and lignin extractions and specialized polymer feature alterations from the optimal pretreatments as described above (Figs. 5 and 7).

**Fig. 10:**
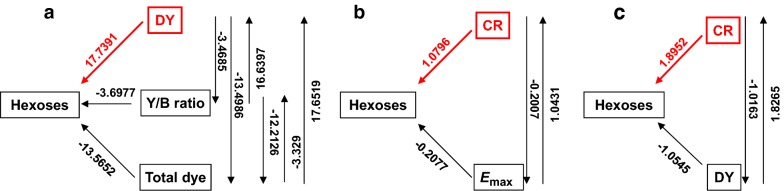
Path coefficient assay between major factors of biomass porosity and hexose yields released from enzymatic hydrolysis in the raw materials and three optimal pretreated biomass residues of four pairs of *Miscanthus* samples. **a** Path coefficient assay between hexose yields and Simons’ stains (DY, Y/B ratio, total dye). **b** Path coefficient assay between hexose yields and Congo red (CR)/enzyme adsorbed (*E*_max_). **c** Path coefficient assay between hexose yields and CR/DY (*n* = 32)

Although biomass porosity could be characterized by multiple assays as described above, little is yet reported about the key factor(s) of biomass porosity that is able to estimate biomass enzymatic saccharification under LHW and chemical pretreatments precisely. Taking advantage of four representative pairs of *Miscanthus* samples, we then performed a path coefficient assay between biomass porosity and hexose yields released from enzymatic hydrolysis after three optimal pretreatments (Fig. 10). In terms of Simons stain, the yellow dye (DY) showed a predominantly positive impact on hexoses yields, whereas other two factors (total dye and Y/B ratio) had relatively lower coefficient values (Fig. 10a). Meanwhile, path coefficient assay was conducted between Congo red (CR) and enzyme (mixed-cellulase) adsorption (*E*_max_), which were able to measure the surface area of cellulose (Fig. 10b). By comparison, CR had much higher coefficient value than that of *E*_max_, suggesting that the CR should be more precise to account for biomass enzymatic saccharification. Notably, regardless of both DY and CR showing the major impacts on hexose yields, the CR had much higher path coefficient value than that of DY, suggesting that CR should be a relatively more precise factor on biomass enzymatic saccharification upon three optimal pretreatments performed in this study (Fig. 10c).

### Integrative impact on biomass enzymatic saccharification

**Fig. 11:**
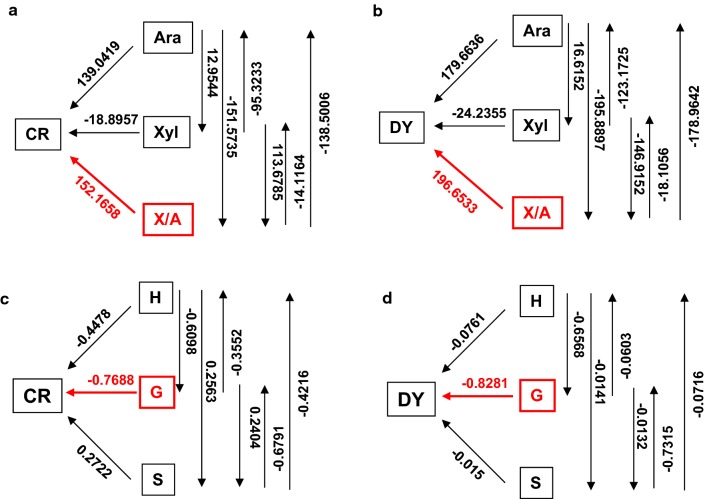
Path coefficient assay between wall polymer features and major factors of biomass porosity in the raw materials and three optimal pretreated biomass residues of four pairs of *Miscanthus* samples. **a**, **b** Path coefficient assay between hemicellulose monosaccharides (Ara, Xyl, A/X ratio) and Congo red (CR)/yellow dye (DY). **c**, **d** Path coefficient assay between lignin monomers (H, G, S) and CR/DY (*n* = 32)

**Fig. 12:**
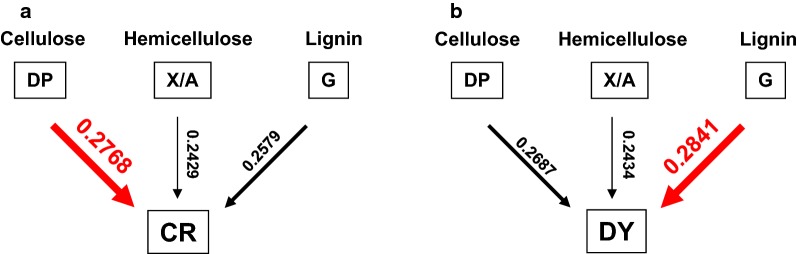
Gray correlation assay between three wall polymer features (cellulose DP, hemicellulose X/A, and lignin G-monomer) and two factors (DY and CR) of biomass porosity in the raw materials and three optimal pretreated biomass residues of four pairs of *Miscanthus* samples. **a** Gray correlation assay between CR and three wall polymer features. **b** Gray correlation assay between DY and three wall polymer features (*n* = 32)

As described above, three optimal pretreatments appear to distinctively extract wall polymers and alter residual polymer features, leading to characteristic enhancements of biomass porosity. To understand how biomass porosity was increased from the optimal pretreatments, path coefficient assay was also conducted between wall polymer features and biomass porosity using the pretreated biomass samples (Fig. 11). With respect to the hemicellulose features, X/A ratio showed a predominant contribution to CR and DY, whereas arabinose (Ara) and xylose (Xyl) exhibited relatively less contribution in particular about Xyl (Fig. 11a, b). In terms of the three lignin monomers, the G-monomer proved to be a significant contributor of CR and DY (Fig. 11c, d). For cellulose features (CrI, DP), it is assumed that cellulose DP should mainly affect biomass porosity, because cellulose CrI did not show significant correlation with hexose yields as described above (Additional file 1: Table S15). As a consequence, gray coefficient assay was performed between the identified three major wall polymer features and two key factors (CR, DY) of biomass porosity as examined above (Fig. 12). By comparison, cellulose DP showed a more significant contribution to the CR (Fig. 12a), G-monomer had higher contribution to the DY (Fig. 12b), and X/A showed a similar contribution to both CR and DY. Furthermore, based on the Spearman correlation analyses, all three significant wall polymer features (DP, X/A, G-monomer) exhibited negative correlations with CR and DY at *P *< 0.05 or 0.01 level (Additional file 1: Table S16), consistent with the findings that the three optimal pretreatments primarily reduced those wall polymer features which significantly increased biomass porosity in the pretreated biomass residues.

**Fig. 13:**
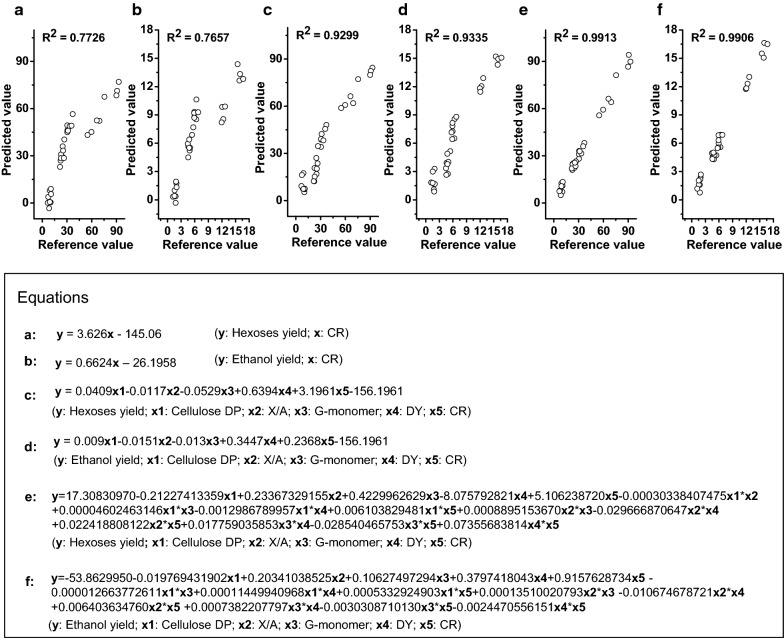
Regression calculation of equations to estimate hexoses and ethanol yields in the raw materials and three optimal pretreated biomass residues of four pairs of *Miscanthus* samples (*n* = 32). **a** Equation between CR of biomass porosity and hexose yields; **b** equation between CR of biomass porosity and ethanol yields; **c** equation between five factors (CR and DY of biomass porosity and three major wall polymer features) and hexose yields; **d** equation between five factors and ethanol yields; **e** equation between hexose yields and five factors plus their interactions with each other. **f** Equation between ethanol yields and five factors plus their interactions with each other

To directly compare biomass porosity and wall polymer features impacts on biomass saccharification, this study performed regression analyses to generate various equations accounting for the hexoses yields released from enzymatic hydrolysis of all biomass residues after three optimal pretreatments, as well as the bioethanol yields obtained from final yeast fermentation (Fig. 13). To sort out the CR-independent impacts on hexoses and bioethanol yields, two equations were generated with high *R*^2^ values at 0.77 (Fig. 13a, b), strongly confirming that CR was a predominant positive factor on biomass saccharification. However, while all other factors were co-calculated including three significant wall polymer features (DP, X/A, G-monomer) and DY, the two generated equations had much increased *R*^2^ values at 0.93 (Fig. 13c, d), confirming that all other factors should play an additional role in biomass enzymatic hydrolysis. Notably, while interactions among all factors were co-calculated, the two equations had *R*^2^ values of 0.99 (Fig. 13e, f), presenting an excellent regression model to precisely estimate hexoses and bioethanol yields from three optimal pretreatments. Hence, the results have demonstrated an integrative determinant on biomass enzymatic saccharification and bioethanol production from a predominant factor (CR) and other additional significant factors (DY, DP, X/A, G-monomer). It also indicated that the equations established in this study should be applicable as a novel standard to judge any types of biomass process technology for cost-effective biofuels production in all bioenergy crops.

### The mechanism for complete biomass saccharification and highest bioethanol production

**Fig. 14:**
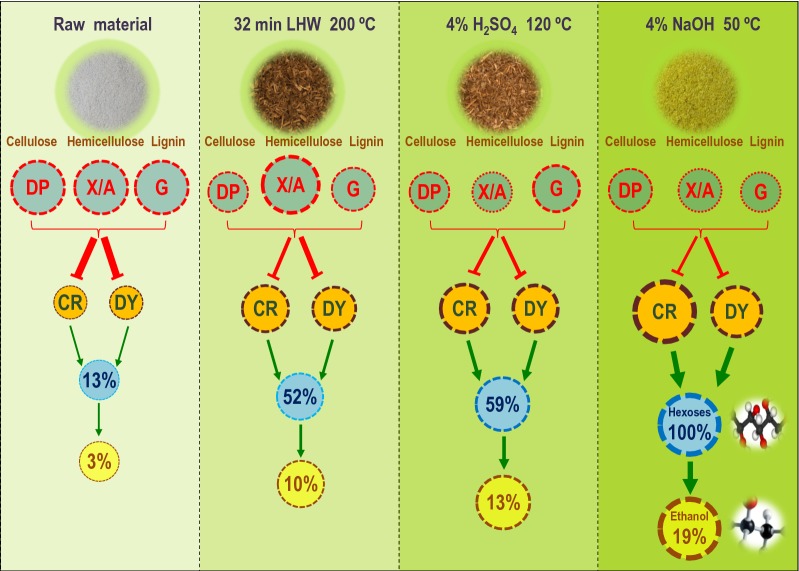
A hypothetical model to highlight an integrative impact on biomass saccharification and bioethanol production subjective to a predominate factor (CR stain) of biomass porosity and four additional minor factors (DY stain, cellulose DP, hemicellulose X/A, lignin G-monomer) under three optimal pretreatments in bioenergy *Miscanthus* and beyond

To understand how complete biomass saccharification could be achieved to acquire the highest bioethanol production from optimal LHW and chemical pretreatments, we proposed a hypothetical model subjected to all major findings obtained in this study (Fig. 14). (1) Compared to the control (raw materials), the optimal NaOH pretreatment mostly extracted lignin and hemicelluloses (Fig. 5), leading to reduced major wall polymer features (cellulose DP, hemicellulosic X/A, lignin G-monomer), which predominately increased CR of biomass porosity with a raised DY for almost complete biomass enzymatic saccharification with the hexoses yield close to 100% (% cellulose) and the highest bioethanol yield at 19% (% dry matter). (2) As compared with the optimal NaOH pretreatment, the optimal H_2_SO_4_ pretreatment extracted significantly less lignin with higher G-monomer content, which caused much lower CR and DY for hexoses and bioethanol yields at 59% and 13%. (3) Compared to the optimal H_2_SO_4_ pretreatment, the LHW pretreatment led to much higher X/A for slightly lower CR, resulting in the hexoses and ethanol yields at 52% and 10%. Hence, this model has outlined a distinct alteration of three major wall polymer features from three optimal pretreatments and indicated a predominant impact of biomass porosity on sequential biomass enzymatic saccharification and final bioethanol production in bioenergy *Miscanthus*.

## Conclusion

In this study, three optimal (LHW, H_2_SO_4_, and NaOH) pretreatments were performed in four typical pairs of *Miscanthus* accessions, and mild alkali pretreatment (4% NaOH at 50 °C for 2 h) could lead to almost complete biomass saccharification with hexose yields close to 100% (% cellulose) while 1% Tween-80 was co-supplied into enzymatic hydrolysis. Notably, the highest bioethanol yields at 19% (% dry matter) were obtained from yeast fermentation in three *Miscanthus* accessions with much high sugar–ethanol conversion rates by 94–98%. Chemical analyses indicated that three optimal pretreatments primarily extracted hemicelluloses and lignin, and distinctively altered major wall polymer features, leading to significantly increased biomass porosity accounting for much-enhanced biomass enzymatic saccharification, in particular from the optimal alkali pretreatment. Based on the regression analyses of major wall polymer features and biomass porosity factors, equations were generated as an excellent standard to precisely estimate hexoses and bioethanol yields under three optimal pretreatments, and a hypothetical model was then raised to highlight an integrative determinant on biomass saccharification and bioethanol production including a predominant factor of biomass porosity and other four minor factors. It has thus provided an applicable standard to judge any types of biomass pretreatments for high biofuel production in *Miscanthus* and beyond.

## Methods

### Collection of biomass samples

Four representative pairs of *Miscanthus* samples were selected from *Miscanthus* germplasm accessions collected in 2007 in China [18, 23, 26]. The mature *Miscanthus* samples were harvested from Huazhong Agricultural University experimental field. The stem tissues were first inactivated at 105 °C for 10 min and dried at 60 °C until constant weight. To remove sample heterogeneity, the dried tissues were ground through a 40-mesh screen for uniform biomass digestibility. Four pairs of *Miscanthus* samples with distinct cell wall composition and varied biomass digestibility were selected (Table 1) and stored in the container until use.

### Wall polymer extraction and assay

Wall polysaccharides were extracted as previously described by Peng et al. [45] with minor modification by Li et al. [7]. After removals of soluble sugars, lipid, starch, and pectin from successive extractions with phosphate buffer (pH 7.0), chloroform–methanol (1:1, v/v), dimethyl sulphoxide (DMSO)–water (9:1, v/v), and ammonium oxalate (0.5%, w/v), the remaining crude cell walls were suspended in 4 M KOH containing NaBH_4_ (1.0 mg/mL) and the combined supernatants were used as KOH-extractable hemicellulose fraction. The remaining pellets were suspended in 67% H_2_SO_4_ (v/v) for 1 h at 25 °C to determine hexoses as cellulose level. Total hemicelluloses were determined based on hexoses and pentoses in the hemicellulose fraction and pentoses in the remained cellulose pellets. UV–VIS Spectrophotometer (V-1100D, Shanghai MAPADA Instruments Co., Ltd., Shanghai, China) was employed for detection of hexoses and pentoses. Anthrone/H_2_SO_4_ and orcinol/HCl methods were, respectively, used for hexoses and pentoses assay [23]. In terms of the high pentoses content interfering the absorbance reading at 620 nm for hexoses assay, the deduction from pentoses reading at 660 nm was conducted for final hexoses calculation. Total lignin, consisting of acid-soluble and insoluble lignin, was measured using Laboratory Analytical Procedure of the National Renewable Energy Laboratory as previously described by Xu et al. [25]. All analyses were performed in independent biological triplicate.

### Detection of cellulose CrI and DP

Cellulose crystallinity index (CrI) was determined using X-ray diffraction (XRD) method (Rigaku-D/MAX, Ultima III; Japan) as previously described by Xu et al. [25] and Li et al. [7]. The powder samples laid on the glass holder were analyzed under plateau conditions. Ni-filtered Cu-Kα radiation (*λ* = 0.154056 nm) generated at voltage of 40 kV and current of 18 mA, and the scans at speed of 0.0197°/s from 10° to 45° were employed to collect diffraction data for the estimation of CrI using Eq. (1), where *I*_200_ is the intensity of the 200 peak at 2*θ* around 22.5°, which represents both crystalline and amorphous materials, while *I*_am_ is the minimum intensity of amorphous material between the 200 and 110 peaks at 2*θ* around 18.5°. The standard deviation (SD) of XRD method for CrI was detected at ± 0.05–0.15 (*n* = 15).

1 $${\text{CrI }}(\% ) = (I_{ 2 0 0} - I_{\text{am}} ) \times 1 0 0 /I_{ 2 0 0}$$

The cellulose DP was determined using the viscosity method and confirmed by gel-permeation chromatography method as previously described by Zhang et al. [27] and Sun et al. [11]. The well-mixed biomass powders (0.2–1.0 g) were extracted with 10 mL 4 M KOH (containing 1.0 mg/mL NaBH_4_) at 25 °C for 1 h, and then centrifuged (3000×*g*) for 5 min. The residues were washed once with 4 M KOH, and five times with distilled water until pH 7.0. The remaining residues were incubated with 10 mL 8% NaClO_2_ at 25 °C for 72 h (fresh NaClO_2_ solution was changed every 12 h). The sample residues were then washed at least five times with distilled water until at pH 7.0, and dried at 38 °C with vacuum suction filtration. The crude cellulose DP was measured at 25 ± 0.5 °C using cupriethylenediamine hydroxide (Cuen) as the solvent in Ubbelohde viscometer. The relative viscosity (*ƞ*_rel_) values were calculated using the ratio of *t*/*t*_0_, where *t* and *t*_0_ are the efflux times for the cellulose solution and Cuen (blank) solvent, respectively. The intrinsic viscosity was calculated by interpolation using the United States Pharmacopeia table (USP, 2002) that files the predetermined values of the product of intrinsic viscosity and concentration. The intrinsic viscosity values were converted to cellulose DP according to Eq. (2). All experiments were carried out in biological triplicate. For equation [2], [*ƞ*] is the intrinsic viscosity of the solution calculated by interpolation using the USP table.

2 $${\text{DP}}^{0.905} = 0.75\left[ \eta \right]$$

### Determination of hemicellulosic monosaccharides and lignin monomers

Monosaccharides of hemicelluloses were determined by GC–MS (SHIMADZU GCMS-QP2010 Plus) method as previously described by Xu et al. [25] and Li et al. [8]. Three monomers of lignin were analyzed by HPLC method (1525, Waters Corp., MA, USA) as described by Wu et al. [35] and Li et al. [10].

### Scanning electron microscopy (SEM) and Fourier transform infrared (FTIR) spectroscopy

The biomass morphology was observed using SEM (SEM JSM-IT300, Akishima, Tokyo, Japan). Well-mixed sample residues collected after pretreatments and enzymatic hydrolysis were sputter-coated with gold in a JFC-1600 ion sputter (Mito City, Japan) and visualized for 5–8 times to acquire representative images. FTIR spectroscopy was performed to observe the structural constituents and chemical linkages in the representative raw and pretreated *Miscanthus* samples. A Perkin-Elmer spectrophotometer (NEXUS 470, Thermo Fisher Scientific, Waltham, MA, USA) was used to qualitatively monitor the samples through spectroscopic grade potassium bromide (KBr) pellet. The well-dried biomasses were finely powdered to reduce scattering losses and deformations in absorption band. The samples (2–4 mg) were dispersed in KBr at the weight ratio of 1:100 and subsequently pressed to produce a transparent pelletized disc by applying 1 Mpa pressure for at least 2 min. The pelletized disc samples were positioned in the path of IR light and the spectra were recorded in absorption mode over 32 scans at a resolution of 4 cm^−1^ in the range of 4000 to 400 cm^−1^ region.

### Measurement of biomass porosity

#### Solvent exchange drying

Organic solvents were used to dry biomass samples by solvent exchange method in order to minimize irreversible pore collapse due to hornification during classical oven-drying practices. The samples were first soaked in Milli-Q distilled water overnight and filtered through a nylon filter of 160 mesh sizes. The water saturated samples were then soaked in methanol overnight to allow solvent exchange with methanol. The samples were recovered by centrifuging at 3000×*g* to remove the supernatant and washed twice with pure methanol. Afterward, the samples were allowed again to soak in anhydrous acetone overnight. The samples were then washed with anhydrous acetone twice, dehydrated in the hood overnight, and further dried in the oven at 50 °C for 2 h. The dried samples were stored until further use.

Simons’ stain (SS) was applied to determine the overall accessible surface area of lignocellulose biomass as previously described by Chandra et al. [14] and Sun et al. [11] with minor modification in this study. Direct Blue 15 (DB15, Phenamine Sky Blue A Conc) and Direct Yellow 11 (DY11) were purchased from Pylam Products Co. Inc., Garden City, NY. The fractionation of the yellow dye to remove the low molecular weight was performed using 100 kDa (molecular weight cut-off) ultracentrifugation membrane [41]. The samples (0.10 g) were added with 1 mL Alum saline solution (5 mM KAl (SO_4_)_2_ + 1.5 mM NaCl) in 15 mL polypropylene centrifuge tubes. A set of tubes with 1:1 solution of DB (blue dye) and DY (yellow dye) were prepared by adding the same amount of each dye solution (10 mg/mL) in a series of volumes (0.25, 0.50, 0.75, 1.0, 1.5 mL) to each sample. Distilled water was added to make the final volume up to 10 mL and incubated for 9 h at 70 °C and 200 rpm. The dye adsorption isotherm was measured from this gradient concentration of dyes. After the solutions were cooled at room temperature and centrifuged at 8000×*g*, the absorbance of the supernatants was measured on UV-1100 spectrophotometer at 612.5 nm and 410.5 nm, which are the wave lengths of maximum absorbance for DB15 and DY11. The concentration of DB and DY dyes in supernatants was calculated by Lambert–Beer law for binary solution using Eqs. (3) and (4). The dyes adsorption capacity of biomasses was determined between the initial added dyes and the final free dyes in the supernatants using Eq. (5). The maximum DB and DY dyes adsorbed to the biomasses were calculated by monolayer Langmuir adsorption model (Eq. 6).

3 $$A_{{410.5{\text{nm}}}} = \varepsilon_{{{\text{Y}}/410.5}} {\text{LC}}_{\text{Y}} + \varepsilon_{{{\text{B}}/410.5}} {\text{LC}}_{\text{B}}$$

4 $$A_{{612.5{\text{nm}}}} = \varepsilon_{{{\text{Y}}/612.5}} {\text{LC}}_{\text{Y}} + \varepsilon_{{{\text{B}}/612.5}} {\text{LC}}_{\text{B}}$$

5 $${\text{Ae}} = \left( {{\text{Ci}} - {\text{Ce}}} \right) \times V/\left( {M \times 1000} \right)$$

6 $$\left[ C \right]/\left[ A \right] = 1/K_{\text{ads}} \left[ A \right]_{ \text{max} } + \left[ C \right]/\left[ A \right]_{ \text{max} }$$

where *A* is the absorbance of the supernatant from dye mixture at 410.5 or 612.5 nm, *ε* is the extinction coefficient of each dye at the respective wavelength, and *L* is the path length equals to the cuvette width (1 cm). The extinction coefficients were calculated by preparing standard curves of each dye and measuring the slope of their absorbance at 410.5 and 612.5 nm. The calculated values used in this study were *ε*_Y/410.5_ = 33.50, *ε*_B/410.5_ = 3.56, *ε*_Y/612.5_ = 0.13, *ε*_B/612.5_ = 24.66 L/g/cm. Ae is the amount of dye adsorbed onto the biomass at equilibrium (mg/g), Ci is the initial dye concentration added (mg/L), Ce is the dye concentration in solution at equilibrium (mg/L), *M* is the mass of biomass used (g), and *V* is the total volume of dye mixture (mL). [*C*] is the free dye concentration at equilibrium (mg/mL), [*A*] is the amount of dye adsorbed by the substrate (mg/mg), [*A*]_max_ is the maximum amount of dye adsorbed onto the biomass sample (mg/g), and *K*_ads_ is the monolayer adsorption equilibrium constant. All samples were carried out in technological duplicate.

Congo Red (CR) stain was applied to estimate cellulosic surface area as previously described by Wiman et al. [15]. The samples (100 mg) were treated with dye solution in a series of increasing concentrations (0.25, 0.50, 0.75, 1.0, 1.50 mg/mL) in 0.3 M phosphate buffer (pH 6) with 1.4 mM NaCl and an incubation temperature of 60 °C for 24 h. After centrifugation at 8000×*g*, the absorbance of the supernatant was measured at 498 nm and the maximum amount of adsorbed dye was calculated by subtraction of free dye in the supernatant from the initial added dye (Eqs. 5 and 6).

Cellulose accessibility relationship to cellulase was determined based on the maximum enzyme adsorption by the substrate under non-catalytic condition according to the method described by Goshadrou and Lefsrud [22] with minor modification in this study. The samples were thoroughly mixed with 0.2 M Na-acetate buffer (pH 4.8) containing different amounts of dissolved mixed-cellulase enzymes (Imperial Jade Biotechnology Co., Ltd. Ningxia 750002, China) ranging from 0.5 to 3 mg/mL with 1% substrate consistency. All the samples were incubated at low temperature (4 °C) for 15 h to inhibit hydrolysis of the substrates with gentle shaking after every 3 h to increase enzyme contact with the substrate. After centrifugation at 3000×*g* for 15 min, the supernatants were used for protein analysis by Bradford protein assay with bovine serum albumin (BSA) as a protein standard. The adsorbed enzyme was calculated from the difference of initial enzyme added and free enzyme remained in the supernatant using Eq. (5). The maximum enzyme adsorbed on biomass was measured from the monolayer adsorption isotherm by Eq. (6).

Nitrogen porosimetry was carried out to determine Brunauer–Emmett–Teller (BET) surface area and Barrett, Joyner, and Halenda (BJH) pore volume of biomass samples using a multipurpose apparatus Micromeritics ASAP 2460 analyzer (Micromeritics Instrument Corp., Norcross, GA, USA) as described by Li et al. [3] with minor modification. After degassing the samples at 60 °C for 12 h, the samples were cooled in the presence of nitrogen to allow nitrogen gas condensed on the biomass surfaces and within the pores of samples. Each data point along with the isotherm was recorded with a minimum equilibration time of 100 s, allowing the pressure to stabilize in the sample holder. The quantity of condensed gas was measured from the pressure decrease after the samples were exposed to the gas. The surface area and pore volume were measured using the classical BET and BJH methods [46].

### Biomass pretreatments and enzymatic saccharification

LHW and chemical (H_2_SO_4_ and NaOH) pretreatments were, respectively, performed as previously described by Jin et al. [21] and Li et al. [3] with minor modification.

#### LHW pretreatment

The well-mixed biomass powder was added with 2.4 mL distilled water into a Teflon gasket well sealed in stainless steel bombs, and heated for 4, 8, 16, 32, 64, and 96 min at 200 °C and shaken at 15 rpm. The sealed bombs were cool down immediately and the sample liquor was transferred into a 15-mL plastic centrifuge tube making the final volume of 6 mL with distilled water. The supernatant obtained after centrifuging at 3000×*g* for 5 min was collected for the estimation of pentose and hexose yields released from LHW pretreatment.

#### H_2_SO_4_ pretreatment

Total 6 mL of H_2_SO_4_ at various concentrations (1%, 4%, 8%, and 16%, v/v) was added to the well-mixed powder samples in 15-mL centrifuge tubes and autoclaved at 121 °C for 20 min (15 psi), and then incubated for 2 h at 50 °C and 150 rpm. After centrifugation at 3000×*g* for 5 min, the supernatant was collected for the estimation of pentose and hexose yields.

#### NaOH pretreatment

Total 6 mL of NaOH at various concentrations (1%, 2%, 4%, 8%, and 16%, w/v) was added to the well-mixed powder samples in 15-mL centrifuge tubes and incubated for 2 h at 50 °C and 150 rpm. After centrifugation at 3000×*g* for 5 min, the supernatant was collected for the estimation of pentose and hexose yields. Meanwhile, the well-mixed sample incubated with 6 mL distilled water for 2 h at 50 °C and 150 rpm was used as control. The solid:liquid ratio during LHW and chemical pretreatments was 1:8 and 1:20. The remained residues after each pretreatment were washed with distilled water for at least 5 times to reach pH 7.0, and then used for enzymatic hydrolysis as described below. All samples were conducted in independent biological triplicate.

Enzymatic hydrolysis and Tween-80 supplementation: The pretreated biomass samples were washed once with 0.2 M Na-acetate buffer (pH 4.8). The pretreated pellets and control were incubated with the final concentrations of 2 g/L mixed-cellulases (40 mg/g biomass) purchased from Imperial Jade Biotechnology Co., Ltd. Ningxia 750002, China, which has been applied to measure biomass porosity as described above, with cellulases at 13.25 FPU/g biomass and xylanase at 8.40 U/g biomass. Another set of pretreated and control samples were incubated with the same concentration of mixed-cellulases co-supplied with 1% Tween-80. The mixed-cellulase activity was measured based on the filter paper assay following the International Union of Pure and Applied Chemistry (IUPAC) guidelines, 1 FPU = 1 μmol/min of “glucose” (reducing sugars as glucose) formed during the hydrolysis reaction. The measurement of xylanase activity used 1% (w/v) xylan (Sigma-Aldrich Co. LLC, California, USA) as the substrate, 1 U = 1 μmol/min of “xylose” (reducing sugars as xylose) formed during the hydrolysis reaction. The sealed samples with 5% solid loading were shaken for 48 h at 50 °C and 150 rpm. The supernatants obtained after centrifuging at 3000×*g* for 5 min were used for the estimation of total sugar (hexoses and pentoses) yields released from enzymatic hydrolysis. The hexoses yield was calculated by the following Eq. (7).

7 $${\text{Hexoses yield }}\left( {\text{\% }} \right) = {\text{Hexoses released }}\left( {\text{g}} \right)\times 100/{\text{Cellulose content }}\left( {\text{g}} \right)$$

where hexoses released (g) from the substrates during mixed-cellulase hydrolysis reaction for 48 h; cellulose content (g) of the substrates is given in Table 1; all experiments were performed with biological triplicates.

### Yeast fermentation and ethanol estimation

Yeast fermentation was performed using hexoses released from above enzymatic hydrolysis as previously described by Jin et al. [21] and Li et al. [3]. Yeast *Saccharomyces cerevisiae* strain (purchased from Angel Yeast Co., Ltd., Yichang, China) was used in all fermentation reactions, and the dry yeast powder was dissolved in 0.2 M phosphate buffer (pH 4.8) for 30 min to activate inoculums prior to fermentation experiments. The hydrolysates and residues were then inoculated with activated yeast to an initial cell mass concentration of 0.5 g/L (cell dry weight) with cell density of approximately 9.8 × 10^6^ CFU/mL in all fermentation tubes, and the fermentation was performed at 37 °C for 48 h in tubes. Ethanol was measured using K_2_Cr_2_O_7_ method as described by Li et al. [10]. The fermentation liquid was distilled at 100 °C for 10 min, and appropriate amount of ethanol sample was heated in 2 mL 5% K_2_Cr_2_O_7_ for 10 min in a boiling water bath. After cooling at room temperature, the samples were added to distilled water to 10 mL and the absorbance was measured at 600 nm. Absolute ethanol (99.9%) was used as the standard. The sugar–ethanol conversion rate (%) was estimated using the following Eq. (8):

8 $$S - E \left( {\text{\% }} \right) = E/\left( {F \times H} \right) \times 100$$

where *S*–*E* is the sugar–ethanol conversion rate (%), *E* is the total ethanol weight (g) at the end of fermentation, *F* is the theoretical conversion rate of glucose to ethanol, i.e., 0.511, and *H* is the total hexoses weight (g) at the beginning of fermentation. The fermentation experiments were conducted in biological triplicates.

### Statistical interpretation of correlation coefficients

Statistical analysis was carried out using Superior Performance Software Systems (SPSS version 16.0, Inc., Chicago, IL) and Data Processing System (DPS). Correlation coefficients were determined using Spearman’s rank, Path coefficient, and Grey coefficient assays for all measured parameters. Multiple regression models were used to generate the equations. Pair-wise comparisons were conducted between two measurements by Student’s *t* test. Means were separated by least significant difference (LSD) test at *P* = 0.05. The line graph, histogram, and regression analysis for the best-fit curve were generated using Origin 8.5 software (Microcal Software, Northampton, MA). The average values were calculated from the original triplicate measurements for these analyses.

## Additional file

**Additional file 1**: **Table S1.** Hexose yields (% cellulose) released from enzymatic hydrolysis after pretreatments with LHW under a time course in four typical pairs of *Miscanthus* accessions. **Table S2.** Hexose yields (% cellulose) released from enzymatic hydrolysis after H_2_SO_4_ pretreatments with a series concentrations in four typical pairs of *Miscanthus* accessions. **Table S3.** Hexose yields (% cellulose) released from enzymatic hydrolysis after NaOH pretreatments with a series concentrations in four typical pairs of *Miscanthus* accessions. **Table S4.** Hexose yields (% cellulose) released from enzymatic hydrolysis co-supplied with 1% Tween-80 after three optimal pretreatments in four typical pairs of *Miscanthus* accessions. **Table S5.** Bioethanol yields (% dry matter) released from yeast fermentation using total hexoses obtained from enzymatic hydrolysis co-supplied with 1% Tween-80 after three optimal pretreatments in four typical pairs of *Miscanthus* accessions. **Table S6.** Sugar-ethanol conversion rates (%) based on the calculation of total hexoses and ethanol yields obtained from three optimal pretreatments as shown in Table S4 and S5. **Table S7.** Wall polymer levels (% dry matter) of raw materials and the biomass residues obtained after three optimal pretreatments. **Table S8.** Cellulose features (CrI and DP) of raw materials and the biomass residues obtained from three optimal pretreatments. **Table S9.** Hemicellulose monosaccharide composition of raw materials and the biomass residues obtained from three optimal pretreatments. **Table S10.** Three monomer ratios of lignin in raw materials and the biomass residues obtained from three optimal pretreatments. **Table S11.** Characteristic bands of the FTIR spectra in biomass residues as referred from previous studies. **Table S12.** Biomass porosity of raw materials and the biomass residues obtained from three optimal pretreatments in four pairs of *Miscanthus* accessions including Simons stains (DY, DB, Total, Y/B), Congo red dye (CR) and mixed-cellulase enzyme adsorption (*E*_max_). **Table S13.** Pore characteristics (specific surface area and cumulative pore volume) in the representative Pair II of *Miscanthus* samples determined by BET and BJH methods from nitrogen adsorption porosimetry. **Table S14.** Correlation coefficients (Spearman rank) between hexose/ethanol yield and major factors of biomass porosity in raw materials and three optimal pretreated biomass residues of four pairs of samples. **Table S15.** Correlation coefficients (Spearman rank) between hexose/ethanol yield and major wall polymer features in four pairs of *Miscanthus* samples. **Table S16.** Correlation coefficients (Spearman rank) among major wall polymer features and major factors of biomass porosity in four pairs of *Miscanthus* samples.

## Abbreviations

Ara: arabinose
BET: Brunauer–Emmett–Teller
BJH: Barrett–Joyner–Halenda
CR: Congo red
CrI: crystalline index
DP: degree of polymerization
DY: Direct yellow 11
*E*_max_: maximum enzyme adsorbed
G: guaiacyl alcohol
GC–MS: gas chromatography–mass spectroscopy
H: *p*-hydroxyphenyl alcohol
HPLC: high performance liquid chromatography
S: sinapyl alcohol
*S*–*E*: sugar to ethanol conversion rate
SS: Simons’ stain
X/A: xylose-to-arabinose ratio
XRD: X-ray diffraction
Xyl: xylose
Y/B: ratio of yellow and blue dyes

## References

[1] Chen X, Kuhn E, Jennings EW, Nelson R, Tao L, Zhang M, Tucker MP (2016). DMR (deacetylation and mechanical refining) processing of corn stover achieves high monomeric sugar concentrations (230 g/L) during enzymatic hydrolysis and high ethanol concentrations (> 10% v/v) during fermentation without hydrolysate purification or concentration. Energy Environ Sci.

[2] da Costa Sousa L, Jin M, Chundawat SPS, Bokade V, Tang X, Azarpira A, Lu F, Avci U, Humpula J, Uppugundla N, Gunawan C, Pattathil S, Cheh AM, Kothari N, Kumar R, Ralph J, Hahn MG, Wyman CE, Singh S, Simmons BA, Dale BE, Balan V (2016). Next-generation ammonia pretreatment enhances cellulosic biofuel production. Energy Environ Sci.

[3] Li Y, Liu P, Huang J, Zhang R, Hu Z, Feng S, Wang Y, Wang L, Xia T, Peng L (2018). Mild chemical pretreatments are sufficient for bioethanol production in transgenic rice straws overproducing glucosidase. Green Chem.

[4] Huang J, Xia T, Li G, Li X, Li Y, Wang Y, Wang Y, Chen Y, Xie G, Bai FW, Peng L, Wang L (2019). Overproduction of native endo-β-1, 4-glucanases leads to largely enhanced biomass saccharification and bioethanol production by specific modification of cellulose features in transgenic rice. Biotechnol Biofuels.

[5] DeMartini JD, Pattathil S, Miller JS, Li H, Hahn MG, Wyman CE (2013). Investigating plant cell wall components that affect biomass recalcitrance in poplar and switchgrass. Energy Environ Sci.

[6] Yoo CG, Yang Y, Pu Y, Meng X, Muchero W, Yee K, Thompson O, Rodriguez M, Bali G, Engle NL, Lindquist E, Singan V, Schmutz J, DiFazio S, Tschaplinski TJ, Tuskas G, Chen JG, Davison BH, Ragauskas AJ (2017). Insights of biomass recalcitrance in *Populus trichocarpa* natural variants for biomass conversion. Green Chem.

[7] Li F, Xie G, Huang J, Zhang R, Li Y, Zhang M, Wang Y, Li A, Li X, Xia T, Qu C, Hu F, Ragauskas AJ, Peng L (2017). OsCESA9 conserved-site mutation leads to largely enhanced plant lodging resistance and biomass enzymatic saccharification by reducing cellulose DP and crystallinity in rice. Plant Biotechnol J.

[8] Li F, Ren S, Zhang W, Xu Z, Xie G, Chen Y, Tu Y, Li Q, Zhou S, Li Y, Tu F, Liu L, Wang Y, Jiang J, Qin J, Li S, Li Q, Jing HC, Zhou F, Gutterson N, Peng L (2013). Arabinose substitution degree in xylan positively affects lignocellulose enzymatic digestibility after various NaOH/H_2_SO_4_ pretreatments in *Miscanthus*. Bioresour Technol.

[9] Li F, Zhang M, Guo K, Hu Z, Zhang R, Feng Y, Yi X, Zou W, Wang L, Wu C, Tian J, Lu T, Xie G, Peng L (2015). High-level hemicellulosic arabinose predominately affects lignocellulose crystallinity for genetically enhancing both plant lodging resistance and biomass enzymatic digestibility in rice mutants. Plant Biotechnol J.

[10] Li M, Si S, Hao B, Zha Y, Wan C, Hong S, Kang Y, Jia J, Zhang J, Li M, Zhao C, Tu Y, Zhou S, Peng L (2014). Mild alkali-pretreatment effectively extracts guaiacyl-rich lignin for high lignocellulose digestibility coupled with largely diminishing yeast fermentation inhibitors in *Miscanthus*. Bioresour Technol.

[11] Sun D, Alam A, Tu Y, Zhou S, Wang Y, Xia T, Huang J, Li Y, Zahoor, Wei X, Hao B, Peng L (2017). Steam-exploded biomass saccharification is predominately affected by lignocellulose porosity and largely enhanced by Tween-80 in *Miscanthus*. Bioresour Technol.

[12] Meng X, Wells T, Sun Q, Huang F, Ragauskas A (2015). Insights into the effect of dilute acid, hot water or alkaline pretreatment on the cellulose accessible surface area and the overall porosity of *Populus*. Green Chem.

[13] Chandra RP, Arantes V, Saddler J (2015). Steam pretreatment of agricultural residues facilitates hemicellulose recovery while enhancing enzyme accessibility to cellulose. Bioresour Technol.

[14] Chandra R, Ewanick S, Hsieh C, Saddler JN (2008). The characterization of pretreated lignocellulosic substrates prior to enzymatic hydrolysis, part 1: a Modified Simons’ staining technique. Biotechnol Prog.

[15] Wiman M, Dienes D, Hansen MAT, Van Der Meulen T, Zacchi G, Lidén G (2012). Cellulose accessibility determines the rate of enzymatic hydrolysis of steam-pretreated spruce. Bioresour Technol.

[16] Meng X, Foston M, Leisen J, DeMartini J, Wyman CE, Ragauskas AJ (2013). Determination of porosity of lignocellulosic biomass before and after pretreatment by using Simons’ stain and NMR techniques. Bioresour Technol.

[17] Tian D, Chandra RP, Lee JS, Lu C, Saddler JN (2017). A comparison of various lignin-extraction methods to enhance the accessibility and ease of enzymatic hydrolysis of the cellulosic component of steam-pretreated poplar. Biotechnol Biofuels.

[18] Si S, Chen Y, Fan C, Hu H, Li Y, Huang J, Liao H, Hao B, Li Q, Peng L, Tu Y (2015). Lignin extraction distinctively enhances biomass enzymatic saccharification in hemicelluloses-rich *Miscanthus* species under various alkali and acid pretreatments. Bioresour Technol.

[19] Sun Q, Foston M, Meng X, Sawada D, Pingali SV, O’Neill HM, Li H, Wyman CE, Langan P, Ragauskas AJ, Kumar R (2014). Effect of lignin content on changes occurring in poplar cellulose ultrastructure during dilute acid pretreatment. Biotechnol Biofuels.

[20] Zahoor, Sun D, Li Y, Wang J, Tu Y, Wang Y, Hu Z, Zhou S, Wang L, Xie G, Huang J, Alam A, Peng L (2017). Biomass saccharification is largely enhanced by altering wall polymer features and reducing silicon accumulation in rice cultivars harvested from nitrogen fertilizer supply. Bioresour Technol.

[21] Jin W, Chen L, Hu M, Sun D, Li A, Li Y, Zhen H, Zhou S, Tu Y, Xia T, Wang Y, Xie G, Li Y, Bai B, Peng L (2016). Tween-80 is effective for enhancing steam-exploded biomass enzymatic saccharification and ethanol production by specifically lessening cellulase absorption with lignin in common reed. Appl Energy.

[22] Goshadrou A, Lefsrud M (2017). Synergistic surfactant-assisted [EMIM]OAc pretreatment of lignocellulosic waste for enhanced cellulose accessibility to cellulase. Carbohydr Polym.

[23] Huang J, Xia T, Li A, Yu B, Li Q, Tu Y, Zhang W, Yi Z, Peng L (2012). A rapid and consistent near infrared spectroscopic assay for biomass enzymatic digestibility upon various physical and chemical pretreatments in *Miscanthus*. Bioresour Technol.

[24] Lee WC, Kuan WC (2015). *Miscanthus* as cellulosic biomass for bioethanol production. Biotechnol J.

[25] Xu N, Zhang W, Ren S, Liu F, Zhao C, Liao H, Xu Z, Huang J, Li Q, Tu Y, Yu B, Wang Y, Jiang J, Qin J, Peng L (2012). Hemicelluloses negatively affect lignocellulose crystallinity for high biomass digestibility under NaOH and H_2_SO_4_ pretreatments in *Miscanthus*. Biotechnol Biofuels.

[26] Wang Y, Huang J, Li Y, Xiong K, Wang Y, Li F, Liu M, Wu Z, Tu Y, Peng L (2015). Ammonium oxalate-extractable uronic acids positively affect biomass enzymatic digestibility by reducing lignocellulose crystallinity in *Miscanthus*. Bioresour Technol.

[27] Zhang W, Yi Z, Huang J, Li F, Hao B, Li M, Hong S, Lv Y, Sun W, Ragauskas A, Hu F, Peng J, Peng L (2013). Three lignocellulose features that distinctively affect biomass enzymatic digestibility under NaOH and H_2_SO_4_ pretreatments in *Miscanthus*. Bioresour Technol.

[28] Cha YL, Yang J, Park Y, An GH, Ahn JW, Moon YH, Yoon YM, Yu GD, Choi IH (2015). Continuous alkaline pretreatment of *Miscanthus sacchariflorus* using a bench-scale single screw reactor. Bioresour Technol.

[29] Kang KE, Han M, Moon SK, Kang HW, Kim Y, Cha YL, Choi GW (2013). Optimization of alkali-extrusion pretreatment with twin-screw for bioethanol production from *Miscanthus*. Fuel.

[30] Yang SJ, Yoo HY, Choi HS, Lee JH, Park C, Kim SW (2015). Enhancement of enzymatic digestibility of *Miscanthus* by electron beam irradiation and chemical combined treatments for bioethanol production. Chem Eng J.

[31] Yeh RH, Lin YS, Wang TH, Kuan WC, Lee WC (2016). Bioethanol production from pretreated *Miscanthus floridulus* biomass by simultaneous saccharification and fermentation. Biomass Bioenergy.

[32] Huang Y, Wei X, Zhou S, Liu M, Tu Y, Li A, Chen P, Wang Y, Zhang X, Tai H, Peng L, Xia T (2015). Steam explosion distinctively enhances biomass enzymatic saccharification of cotton stalks by largely reducing cellulose polymerization degree in *G. barbadense* and *G. hirsutum*. Bioresour Technol.

[33] Wu L, Li M, Huang J, Zhang H, Zou W, Hu S, Li Y, Fan C, Zhang R, Jing H, Peng L, Feng S (2015). A near infrared spectroscopic assay for stalk soluble sugars, bagasse enzymatic saccharification and wall polymers in sweet sorghum. Bioresour Technol.

[34] Noori MS, Karimi K (2016). Detailed study of efficient ethanol production from elmwood by alkali pretreatment. Biochem Eng J.

[35] Wu Z, Zhang M, Wang L, Tu Y, Zhang J, Xie G, Zhou W, Li F, Guo K, Li Q, Gao C, Peng L (2013). Biomass digestibility is predominantly affected by three factors of wall polymer features distinctive in wheat accessions and rice mutants. Biotechnol Biofuels.

[36] Fan C, Feng S, Huang J, Wang Y, Wu L, Li X, Wang L, Tu Y, Xia T, Li J, Cai X, Peng L (2017). AtCesA8-driven OsSUS3 expression leads to largely enhanced biomass saccharification and lodging resistance by distinctively altering lignocellulose features in rice. Biotechnol Biofuels.

[37] Zahoor, Tu Y, Wang L, Xia T, Sun D, Zhou S, Wang Y, Li Y, Zhang H, Zhang T, Madadi M, Peng L (2017). Mild chemical pretreatments are sufficient for complete saccharification of steam-exploded residues and high ethanol production in desirable wheat accessions. Bioresour Technol.

[38] Wang Q, Wei W, Kingori GP, Sun J (2015). Cell wall disruption in low temperature NaOH/urea solution and its potential application in lignocellulose pretreatment. Cellulose.

[39] Brienzo M, Fikizolo S, Benjamin Y, Tyhoda L, Gorgens J (2017). Influence of pretreatment severity on structural changes, lignin content and enzymatic hydrolysis of sugarcane bagasse samples. Renew Energy.

[40] Zhou X, Li Q, Zhang Y, Gu Y (2017). Effect of hydrothermal pretreatment on *Miscanthus* anaerobic digestion. Bioresour Technol.

[41] Kwok TT, Fogg DN, Realff MJ, Bommarius AS (2017). Applying direct yellow 11 to a modified Simons’ staining assay. Cellulose.

[42] He J, Huang C, Lai C, Huang C, Yong Q (2017). Relations between Moso bamboo surface properties pretreated by kraft cooking and dilute acid with enzymatic digestibility. Appl Biochem Biotechnol.

[43] Yu H, You Y, Lei F, Liu Z, Zhang W, Jiang J (2015). Comparative study of alkaline hydrogen peroxide and organosolv pretreatments of sugarcane bagasse to improve the overall sugar yield. Bioresour Technol.

[44] Huang J, Li Y, Wang Y, Chen Y, Liu M, Wang Y, Zhang R, Zhou S, Li J, Tu Y, Hao B, Peng L, Xia T (2017). A precise and consistent assay for major wall polymer features that distinctively determine biomass saccharification in transgenic rice by near-infrared spectroscopy. Biotechnol Biofuels.

[45] Peng L, Hocart CH, Redmond JW, Williamson RE (2000). Fractionation of carbohydrates in *Arabidopsis* root cell walls shows that three radial swelling loci are specifically involved in cellulose production. Planta.

[46] Barrett EP, Joyner LG, Halenda PP (1951). The determination of pore volume and area distributions in porous substances. 1. Computations from nitrogen isotherms. J Am Chem Soc.

