# Supplementary material for: A finalized determinant for complete lignocellulose enzymatic saccharification potential to maximize bioethanol production in bioenergy Miscanthus
Source: Biotechnol Biofuels. 2019 Apr 27;12:99. doi: 10.1186/s13068-019-1437-4 (PMC6486690; doi:10.1186/s13068-019-1437-4)
Supplement: Supplementary file 1 — Additional file 1: Table S1. Hexose yields (% cellulose) released from enzymatic hydrolysis after pretreatments with LHW under a time course in four typical pairs of Miscanthus accessions. Table S2. Hexose yields (% cellulose) released from enzymatic hydrolysis after H2SO4 pretreatments with a series concentrations in four typical pairs of Miscanthus accessions. Table S3. Hexose yields (% cellulose) released from enzymatic hydrolysis after NaOH pretreatments with a series concentrations in four typical pairs of Miscanthus accessions. Table S4. Hexose yields (% cellulose) released from enzymatic hydrolysis co-supplied with 1% Tween-80 after three optimal pretreatments in four typical pairs of Miscanthus accessions. Table S5. Bioethanol yields (% dry matter) released from yeast fermentation using total hexoses obtained from enzymatic hydrolysis co-supplied with 1% Tween-80 after three optimal pretreatments in four typical pairs of Miscanthus accessions. Table S6. Sugar-ethanol conversion rates (%) based on the calculation of total hexoses and ethanol yields obtained from three optimal pretreatments as shown in Table S4 and S5. Table S7. Wall polymer levels (% dry matter) of raw materials and the biomass residues obtained after three optimal pretreatments. Table S8. Cellulose features (CrI and DP) of raw materials and the biomass residues obtained from three optimal pretreatments. Table S9. Hemicellulose monosaccharide composition of raw materials and the biomass residues obtained from three optimal pretreatments. Table S10. Three monomer ratios of lignin in raw materials and the biomass residues obtained from three optimal pretreatments. Table S11. Characteristic bands of the FTIR spectra in biomass residues as referred from previous studies. Table S12. Biomass porosity of raw materials and the biomass residues obtained from three optimal pretreatments in four pairs of Miscanthus accessions including Simons stains (DY, DB, Total, Y/B), Congo red dye (CR) and mixed-cel [file 13068_2019_1437_MOESM1_ESM.pptx]

## Slide 1
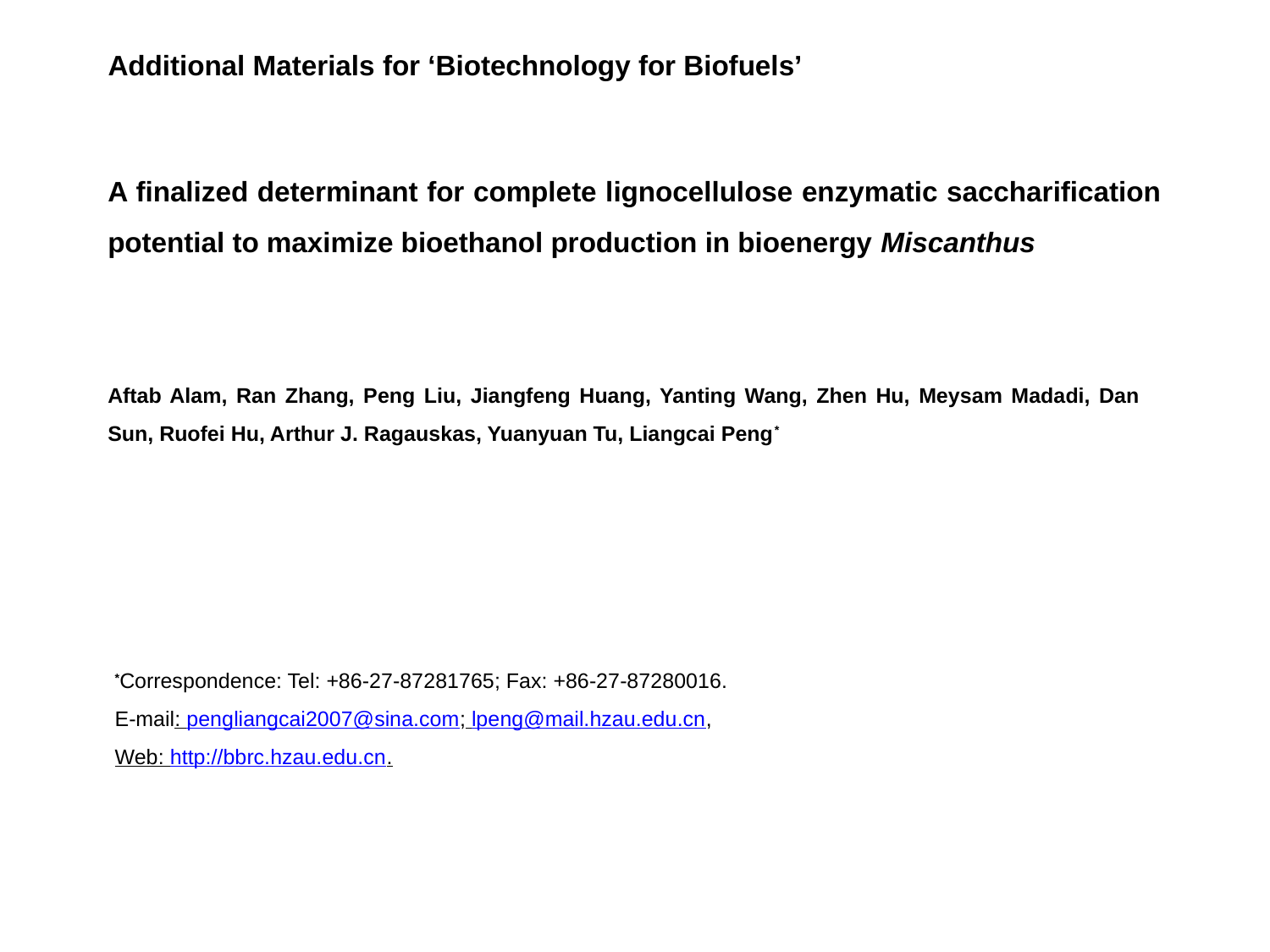

# Additional Materials for ‘Biotechnology for Biofuels’
A finalized determinant for complete lignocellulose enzymatic saccharification potential to maximize bioethanol production in bioenergy Miscanthus
Aftab Alam, Ran Zhang, Peng Liu, Jiangfeng Huang, Yanting Wang, Zhen Hu, Meysam Madadi, Dan Sun, Ruofei Hu, Arthur J. Ragauskas, Yuanyuan Tu, Liangcai Peng*
*Correspondence: Tel: +86-27-87281765; Fax: +86-27-87280016.
E-mail: pengliangcai2007@sina.com; lpeng@mail.hzau.edu.cn,
Web: http://bbrc.hzau.edu.cn.

## Slide 2
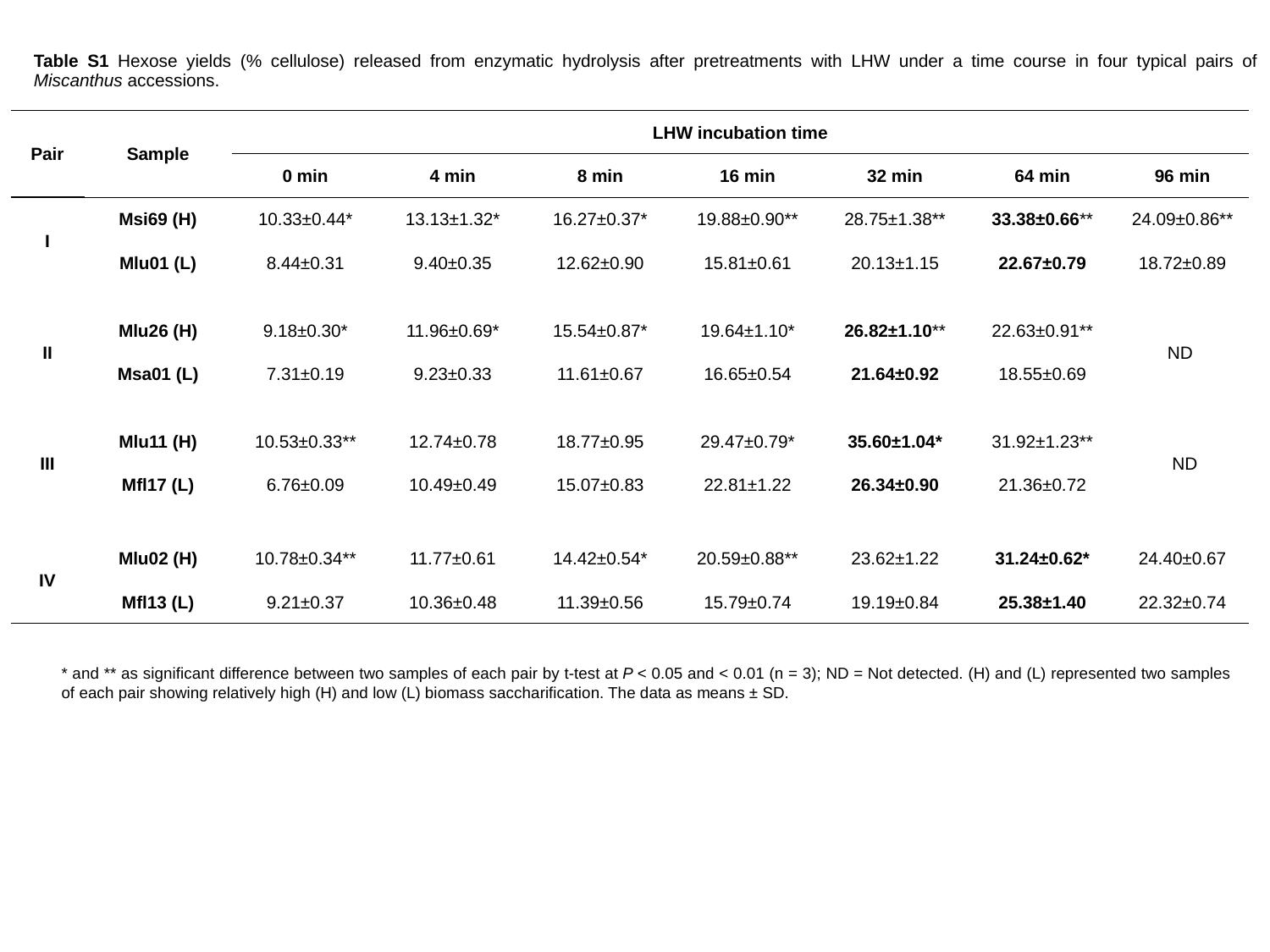

Table S1 Hexose yields (% cellulose) released from enzymatic hydrolysis after pretreatments with LHW under a time course in four typical pairs of Miscanthus accessions.
| Pair | Sample | LHW incubation time | | | | | | |
| --- | --- | --- | --- | --- | --- | --- | --- | --- |
| | | 0 min | 4 min | 8 min | 16 min | 32 min | 64 min | 96 min |
| I | Msi69 (H) | 10.33±0.44\* | 13.13±1.32\* | 16.27±0.37\* | 19.88±0.90\*\* | 28.75±1.38\*\* | 33.38±0.66\*\* | 24.09±0.86\*\* |
| | Mlu01 (L) | 8.44±0.31 | 9.40±0.35 | 12.62±0.90 | 15.81±0.61 | 20.13±1.15 | 22.67±0.79 | 18.72±0.89 |
| | | | | | | | | |
| II | Mlu26 (H) | 9.18±0.30\* | 11.96±0.69\* | 15.54±0.87\* | 19.64±1.10\* | 26.82±1.10\*\* | 22.63±0.91\*\* | ND |
| | Msa01 (L) | 7.31±0.19 | 9.23±0.33 | 11.61±0.67 | 16.65±0.54 | 21.64±0.92 | 18.55±0.69 | |
| | | | | | | | | |
| III | Mlu11 (H) | 10.53±0.33\*\* | 12.74±0.78 | 18.77±0.95 | 29.47±0.79\* | 35.60±1.04\* | 31.92±1.23\*\* | ND |
| | Mfl17 (L) | 6.76±0.09 | 10.49±0.49 | 15.07±0.83 | 22.81±1.22 | 26.34±0.90 | 21.36±0.72 | |
| | | | | | | | | |
| IV | Mlu02 (H) | 10.78±0.34\*\* | 11.77±0.61 | 14.42±0.54\* | 20.59±0.88\*\* | 23.62±1.22 | 31.24±0.62\* | 24.40±0.67 |
| | Mfl13 (L) | 9.21±0.37 | 10.36±0.48 | 11.39±0.56 | 15.79±0.74 | 19.19±0.84 | 25.38±1.40 | 22.32±0.74 |
* and ** as significant difference between two samples of each pair by t-test at P < 0.05 and < 0.01 (n = 3); ND = Not detected. (H) and (L) represented two samples of each pair showing relatively high (H) and low (L) biomass saccharification. The data as means ± SD.

## Slide 3
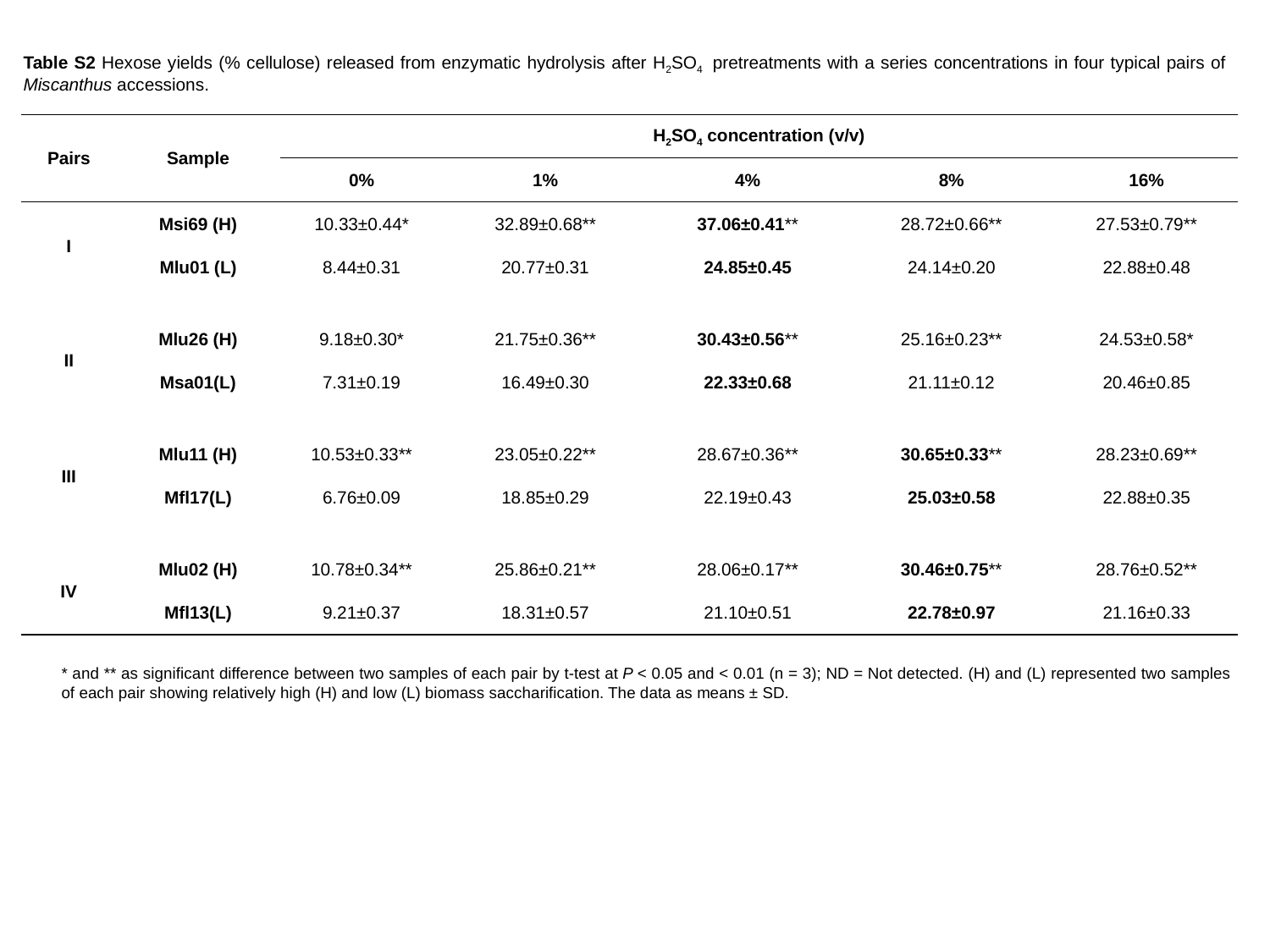

Table S2 Hexose yields (% cellulose) released from enzymatic hydrolysis after H2SO4 pretreatments with a series concentrations in four typical pairs of Miscanthus accessions.
| Pairs | Sample | H2SO4 concentration (v/v) | | | | |
| --- | --- | --- | --- | --- | --- | --- |
| | | 0% | 1% | 4% | 8% | 16% |
| I | Msi69 (H) | 10.33±0.44\* | 32.89±0.68\*\* | 37.06±0.41\*\* | 28.72±0.66\*\* | 27.53±0.79\*\* |
| | Mlu01 (L) | 8.44±0.31 | 20.77±0.31 | 24.85±0.45 | 24.14±0.20 | 22.88±0.48 |
| | | | | | | |
| II | Mlu26 (H) | 9.18±0.30\* | 21.75±0.36\*\* | 30.43±0.56\*\* | 25.16±0.23\*\* | 24.53±0.58\* |
| | Msa01(L) | 7.31±0.19 | 16.49±0.30 | 22.33±0.68 | 21.11±0.12 | 20.46±0.85 |
| | | | | | | |
| III | Mlu11 (H) | 10.53±0.33\*\* | 23.05±0.22\*\* | 28.67±0.36\*\* | 30.65±0.33\*\* | 28.23±0.69\*\* |
| | Mfl17(L) | 6.76±0.09 | 18.85±0.29 | 22.19±0.43 | 25.03±0.58 | 22.88±0.35 |
| | | | | | | |
| IV | Mlu02 (H) | 10.78±0.34\*\* | 25.86±0.21\*\* | 28.06±0.17\*\* | 30.46±0.75\*\* | 28.76±0.52\*\* |
| | Mfl13(L) | 9.21±0.37 | 18.31±0.57 | 21.10±0.51 | 22.78±0.97 | 21.16±0.33 |
* and ** as significant difference between two samples of each pair by t-test at P < 0.05 and < 0.01 (n = 3); ND = Not detected. (H) and (L) represented two samples of each pair showing relatively high (H) and low (L) biomass saccharification. The data as means ± SD.

## Slide 4
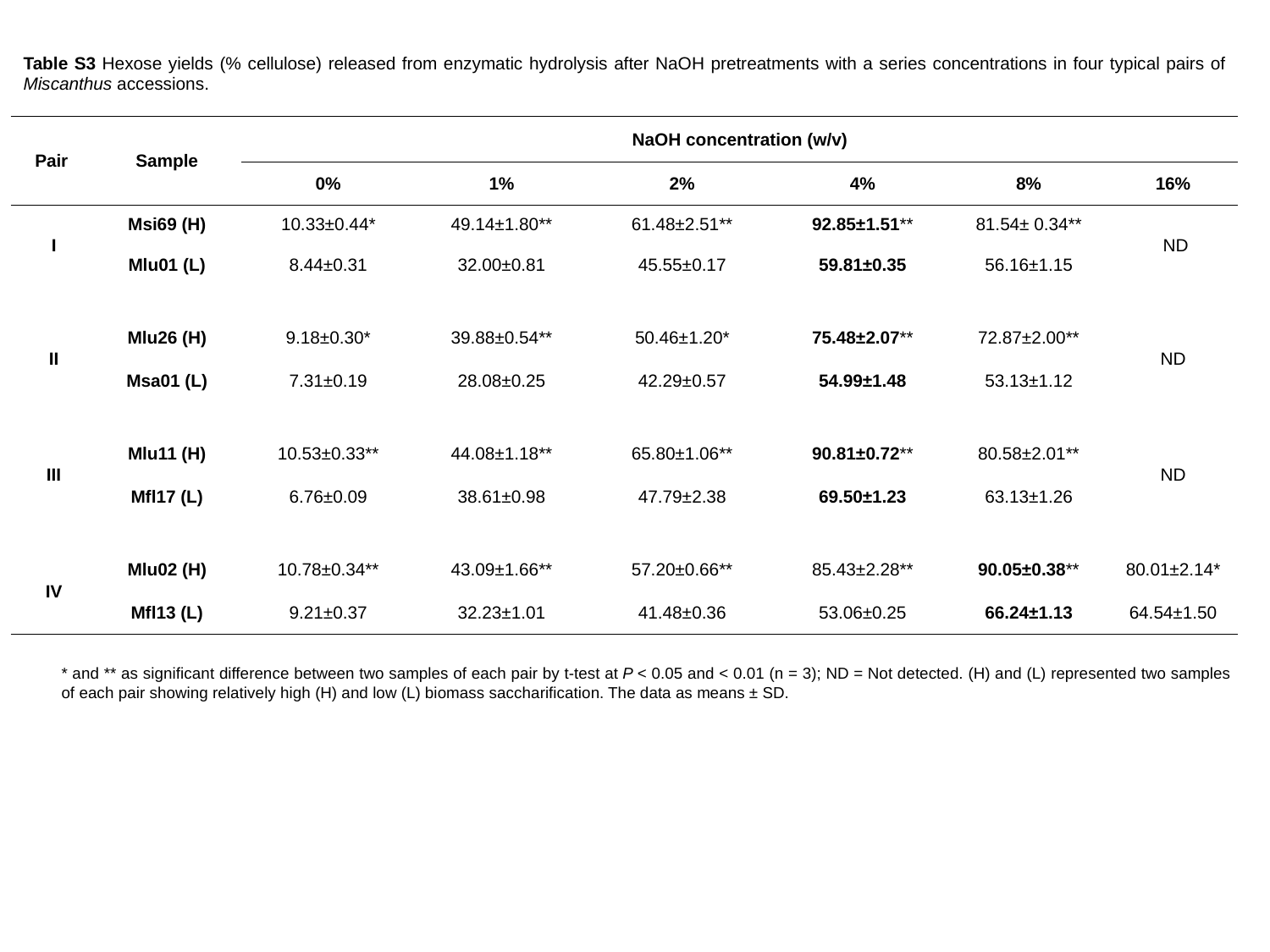

Table S3 Hexose yields (% cellulose) released from enzymatic hydrolysis after NaOH pretreatments with a series concentrations in four typical pairs of Miscanthus accessions.
| Pair | Sample | NaOH concentration (w/v) | | | | | |
| --- | --- | --- | --- | --- | --- | --- | --- |
| | | 0% | 1% | 2% | 4% | 8% | 16% |
| I | Msi69 (H) | 10.33±0.44\* | 49.14±1.80\*\* | 61.48±2.51\*\* | 92.85±1.51\*\* | 81.54± 0.34\*\* | ND |
| | Mlu01 (L) | 8.44±0.31 | 32.00±0.81 | 45.55±0.17 | 59.81±0.35 | 56.16±1.15 | |
| | | | | | | | |
| II | Mlu26 (H) | 9.18±0.30\* | 39.88±0.54\*\* | 50.46±1.20\* | 75.48±2.07\*\* | 72.87±2.00\*\* | ND |
| | Msa01 (L) | 7.31±0.19 | 28.08±0.25 | 42.29±0.57 | 54.99±1.48 | 53.13±1.12 | |
| | | | | | | | |
| III | Mlu11 (H) | 10.53±0.33\*\* | 44.08±1.18\*\* | 65.80±1.06\*\* | 90.81±0.72\*\* | 80.58±2.01\*\* | ND |
| | Mfl17 (L) | 6.76±0.09 | 38.61±0.98 | 47.79±2.38 | 69.50±1.23 | 63.13±1.26 | |
| | | | | | | | |
| IV | Mlu02 (H) | 10.78±0.34\*\* | 43.09±1.66\*\* | 57.20±0.66\*\* | 85.43±2.28\*\* | 90.05±0.38\*\* | 80.01±2.14\* |
| | Mfl13 (L) | 9.21±0.37 | 32.23±1.01 | 41.48±0.36 | 53.06±0.25 | 66.24±1.13 | 64.54±1.50 |
* and ** as significant difference between two samples of each pair by t-test at P < 0.05 and < 0.01 (n = 3); ND = Not detected. (H) and (L) represented two samples of each pair showing relatively high (H) and low (L) biomass saccharification. The data as means ± SD.

## Slide 5
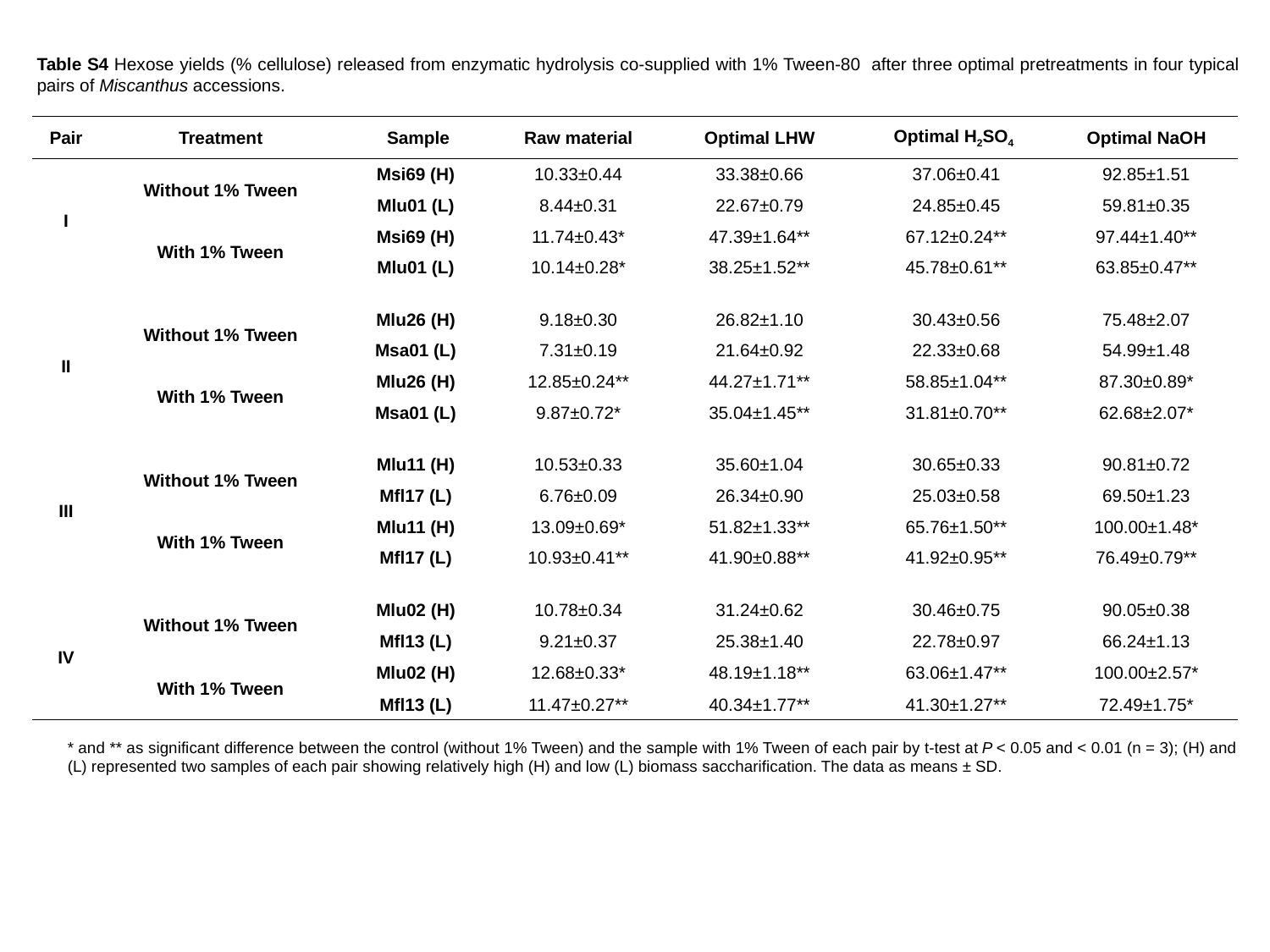

Table S4 Hexose yields (% cellulose) released from enzymatic hydrolysis co-supplied with 1% Tween-80 after three optimal pretreatments in four typical pairs of Miscanthus accessions.
| Pair | Treatment | Sample | Raw material | Optimal LHW | Optimal H2SO4 | Optimal NaOH |
| --- | --- | --- | --- | --- | --- | --- |
| I | Without 1% Tween | Msi69 (H) | 10.33±0.44 | 33.38±0.66 | 37.06±0.41 | 92.85±1.51 |
| | | Mlu01 (L) | 8.44±0.31 | 22.67±0.79 | 24.85±0.45 | 59.81±0.35 |
| | With 1% Tween | Msi69 (H) | 11.74±0.43\* | 47.39±1.64\*\* | 67.12±0.24\*\* | 97.44±1.40\*\* |
| | | Mlu01 (L) | 10.14±0.28\* | 38.25±1.52\*\* | 45.78±0.61\*\* | 63.85±0.47\*\* |
| | | | | | | |
| II | Without 1% Tween | Mlu26 (H) | 9.18±0.30 | 26.82±1.10 | 30.43±0.56 | 75.48±2.07 |
| | | Msa01 (L) | 7.31±0.19 | 21.64±0.92 | 22.33±0.68 | 54.99±1.48 |
| | With 1% Tween | Mlu26 (H) | 12.85±0.24\*\* | 44.27±1.71\*\* | 58.85±1.04\*\* | 87.30±0.89\* |
| | | Msa01 (L) | 9.87±0.72\* | 35.04±1.45\*\* | 31.81±0.70\*\* | 62.68±2.07\* |
| | | | | | | |
| III | Without 1% Tween | Mlu11 (H) | 10.53±0.33 | 35.60±1.04 | 30.65±0.33 | 90.81±0.72 |
| | | Mfl17 (L) | 6.76±0.09 | 26.34±0.90 | 25.03±0.58 | 69.50±1.23 |
| | With 1% Tween | Mlu11 (H) | 13.09±0.69\* | 51.82±1.33\*\* | 65.76±1.50\*\* | 100.00±1.48\* |
| | | Mfl17 (L) | 10.93±0.41\*\* | 41.90±0.88\*\* | 41.92±0.95\*\* | 76.49±0.79\*\* |
| | | | | | | |
| IV | Without 1% Tween | Mlu02 (H) | 10.78±0.34 | 31.24±0.62 | 30.46±0.75 | 90.05±0.38 |
| | | Mfl13 (L) | 9.21±0.37 | 25.38±1.40 | 22.78±0.97 | 66.24±1.13 |
| | With 1% Tween | Mlu02 (H) | 12.68±0.33\* | 48.19±1.18\*\* | 63.06±1.47\*\* | 100.00±2.57\* |
| | | Mfl13 (L) | 11.47±0.27\*\* | 40.34±1.77\*\* | 41.30±1.27\*\* | 72.49±1.75\* |
* and ** as significant difference between the control (without 1% Tween) and the sample with 1% Tween of each pair by t-test at P < 0.05 and < 0.01 (n = 3); (H) and (L) represented two samples of each pair showing relatively high (H) and low (L) biomass saccharification. The data as means ± SD.

## Slide 6
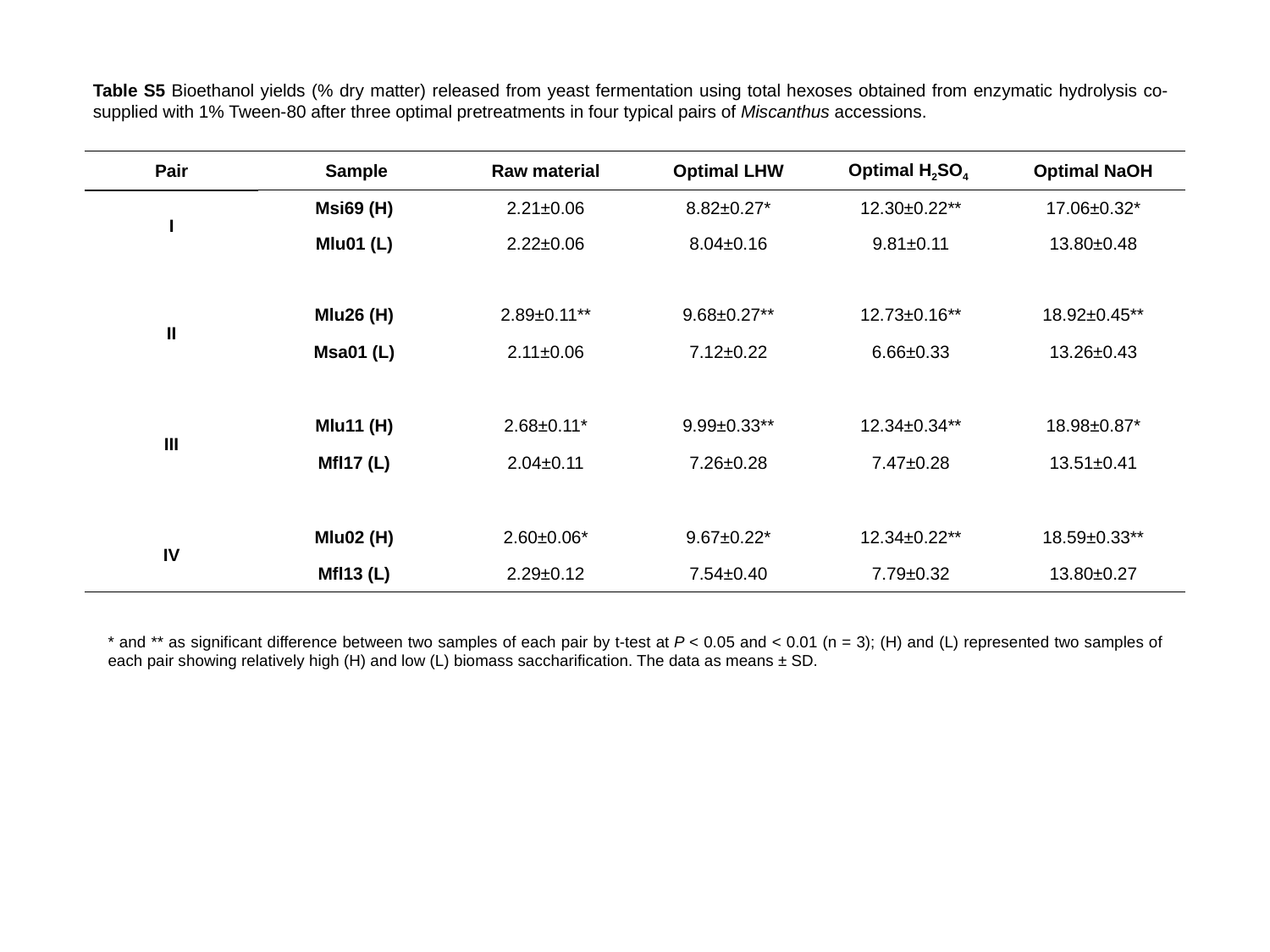

Table S5 Bioethanol yields (% dry matter) released from yeast fermentation using total hexoses obtained from enzymatic hydrolysis co-supplied with 1% Tween-80 after three optimal pretreatments in four typical pairs of Miscanthus accessions.
| Pair | Sample | Raw material | Optimal LHW | Optimal H2SO4 | Optimal NaOH |
| --- | --- | --- | --- | --- | --- |
| I | Msi69 (H) | 2.21±0.06 | 8.82±0.27\* | 12.30±0.22\*\* | 17.06±0.32\* |
| | Mlu01 (L) | 2.22±0.06 | 8.04±0.16 | 9.81±0.11 | 13.80±0.48 |
| | | | | | |
| II | Mlu26 (H) | 2.89±0.11\*\* | 9.68±0.27\*\* | 12.73±0.16\*\* | 18.92±0.45\*\* |
| | Msa01 (L) | 2.11±0.06 | 7.12±0.22 | 6.66±0.33 | 13.26±0.43 |
| | | | | | |
| III | Mlu11 (H) | 2.68±0.11\* | 9.99±0.33\*\* | 12.34±0.34\*\* | 18.98±0.87\* |
| | Mfl17 (L) | 2.04±0.11 | 7.26±0.28 | 7.47±0.28 | 13.51±0.41 |
| | | | | | |
| IV | Mlu02 (H) | 2.60±0.06\* | 9.67±0.22\* | 12.34±0.22\*\* | 18.59±0.33\*\* |
| | Mfl13 (L) | 2.29±0.12 | 7.54±0.40 | 7.79±0.32 | 13.80±0.27 |
* and ** as significant difference between two samples of each pair by t-test at P < 0.05 and < 0.01 (n = 3); (H) and (L) represented two samples of each pair showing relatively high (H) and low (L) biomass saccharification. The data as means ± SD.

## Slide 7
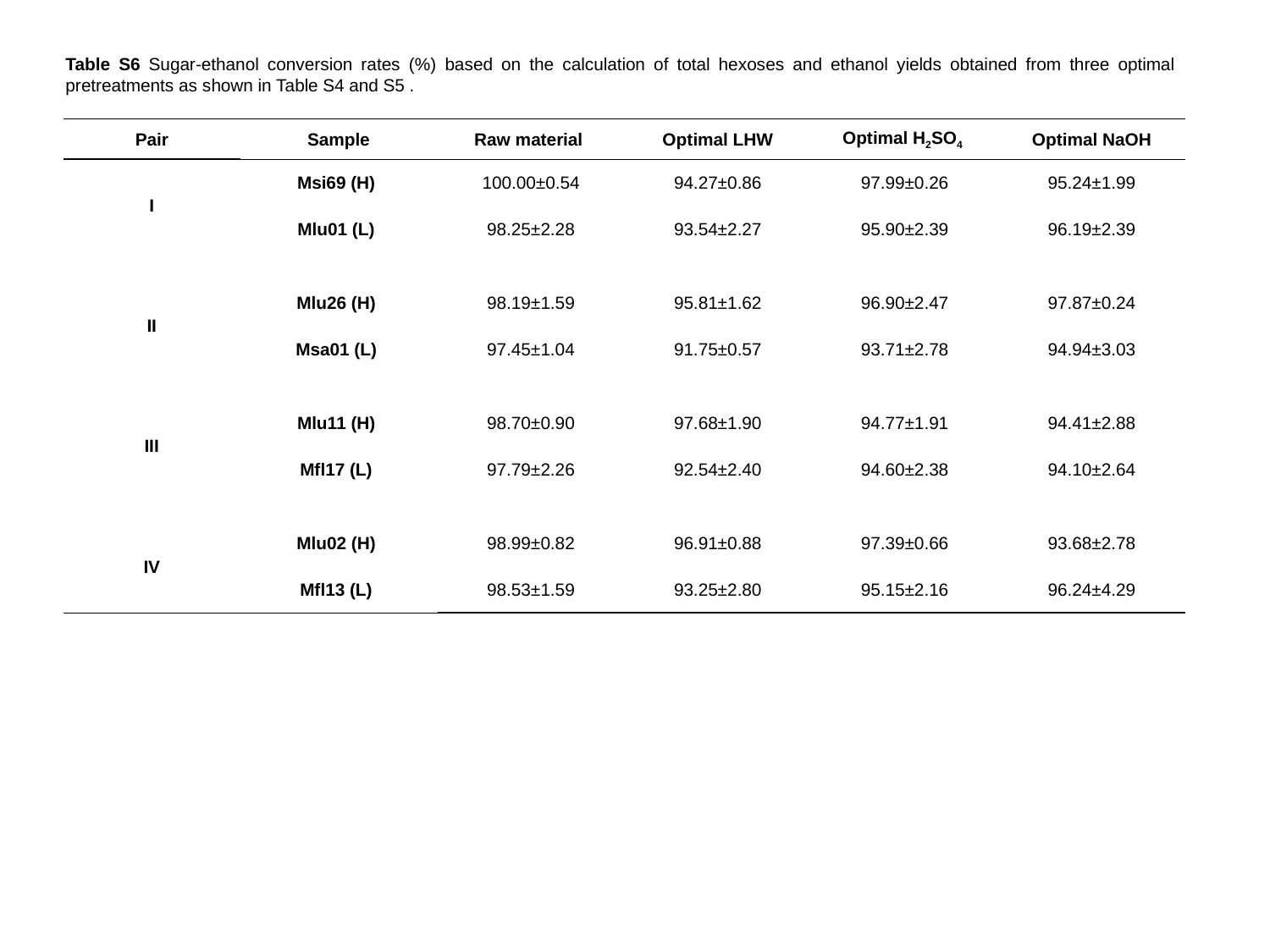

Table S6 Sugar-ethanol conversion rates (%) based on the calculation of total hexoses and ethanol yields obtained from three optimal pretreatments as shown in Table S4 and S5 .
| Pair | Sample | Raw material | Optimal LHW | Optimal H2SO4 | Optimal NaOH |
| --- | --- | --- | --- | --- | --- |
| I | Msi69 (H) | 100.00±0.54 | 94.27±0.86 | 97.99±0.26 | 95.24±1.99 |
| | Mlu01 (L) | 98.25±2.28 | 93.54±2.27 | 95.90±2.39 | 96.19±2.39 |
| | | | | | |
| II | Mlu26 (H) | 98.19±1.59 | 95.81±1.62 | 96.90±2.47 | 97.87±0.24 |
| | Msa01 (L) | 97.45±1.04 | 91.75±0.57 | 93.71±2.78 | 94.94±3.03 |
| | | | | | |
| III | Mlu11 (H) | 98.70±0.90 | 97.68±1.90 | 94.77±1.91 | 94.41±2.88 |
| | Mfl17 (L) | 97.79±2.26 | 92.54±2.40 | 94.60±2.38 | 94.10±2.64 |
| | | | | | |
| IV | Mlu02 (H) | 98.99±0.82 | 96.91±0.88 | 97.39±0.66 | 93.68±2.78 |
| | Mfl13 (L) | 98.53±1.59 | 93.25±2.80 | 95.15±2.16 | 96.24±4.29 |

## Slide 8
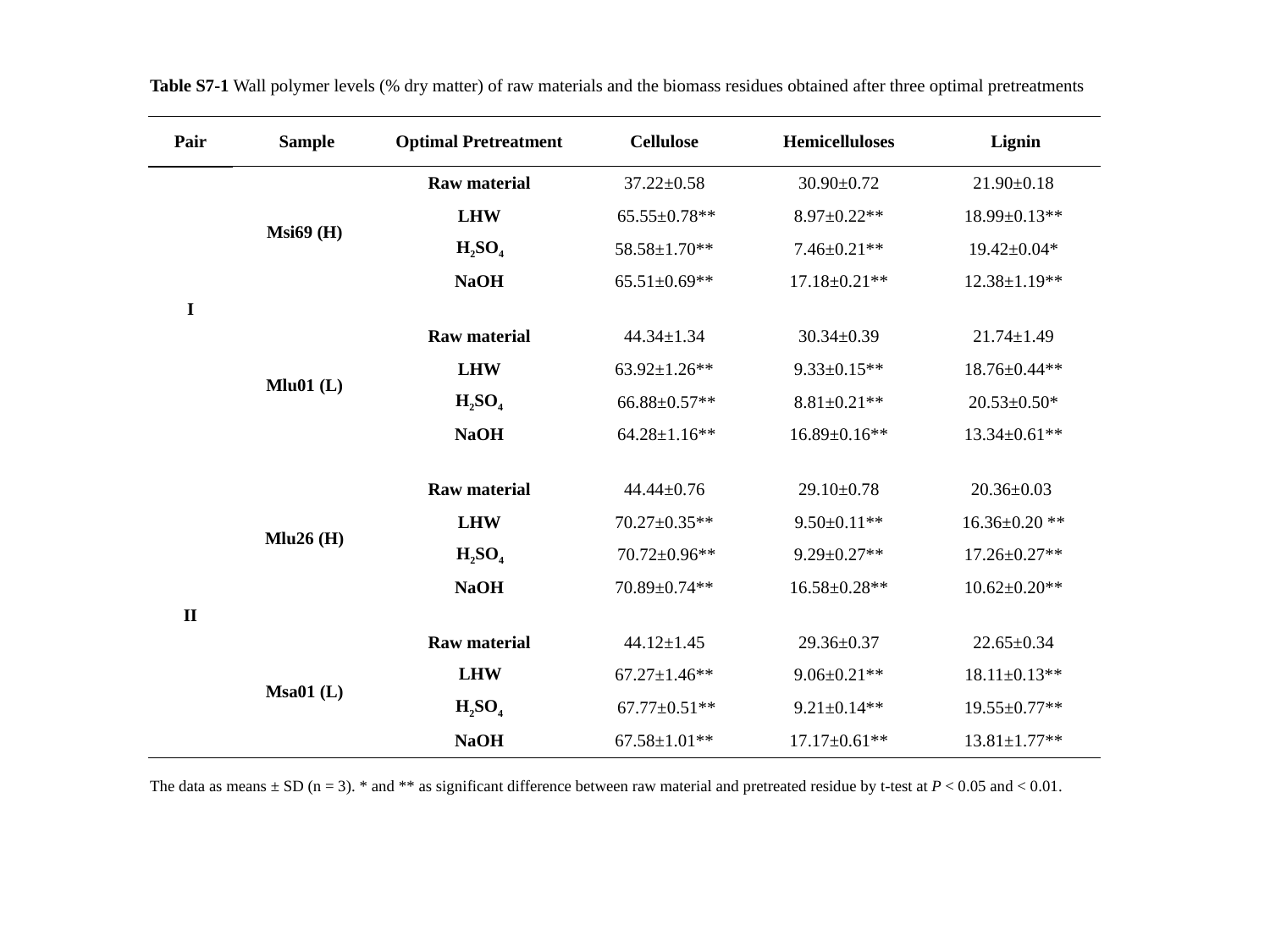

# Table S7-1 Wall polymer levels (% dry matter) of raw materials and the biomass residues obtained after three optimal pretreatments
| Pair | Sample | Optimal Pretreatment | Cellulose | Hemicelluloses | Lignin |
| --- | --- | --- | --- | --- | --- |
| I | Msi69 (H) | Raw material | 37.22±0.58 | 30.90±0.72 | 21.90±0.18 |
| | | LHW | 65.55±0.78\*\* | 8.97±0.22\*\* | 18.99±0.13\*\* |
| | | H2SO4 | 58.58±1.70\*\* | 7.46±0.21\*\* | 19.42±0.04\* |
| | | NaOH | 65.51±0.69\*\* | 17.18±0.21\*\* | 12.38±1.19\*\* |
| | | | | | |
| | Mlu01 (L) | Raw material | 44.34±1.34 | 30.34±0.39 | 21.74±1.49 |
| | | LHW | 63.92±1.26\*\* | 9.33±0.15\*\* | 18.76±0.44\*\* |
| | | H2SO4 | 66.88±0.57\*\* | 8.81±0.21\*\* | 20.53±0.50\* |
| | | NaOH | 64.28±1.16\*\* | 16.89±0.16\*\* | 13.34±0.61\*\* |
| | | | | | |
| II | Mlu26 (H) | Raw material | 44.44±0.76 | 29.10±0.78 | 20.36±0.03 |
| | | LHW | 70.27±0.35\*\* | 9.50±0.11\*\* | 16.36±0.20 \*\* |
| | | H2SO4 | 70.72±0.96\*\* | 9.29±0.27\*\* | 17.26±0.27\*\* |
| | | NaOH | 70.89±0.74\*\* | 16.58±0.28\*\* | 10.62±0.20\*\* |
| | | | | | |
| | Msa01 (L) | Raw material | 44.12±1.45 | 29.36±0.37 | 22.65±0.34 |
| | | LHW | 67.27±1.46\*\* | 9.06±0.21\*\* | 18.11±0.13\*\* |
| | | H2SO4 | 67.77±0.51\*\* | 9.21±0.14\*\* | 19.55±0.77\*\* |
| | | NaOH | 67.58±1.01\*\* | 17.17±0.61\*\* | 13.81±1.77\*\* |
The data as means ± SD (n = 3). * and ** as significant difference between raw material and pretreated residue by t-test at P < 0.05 and < 0.01.

## Slide 9
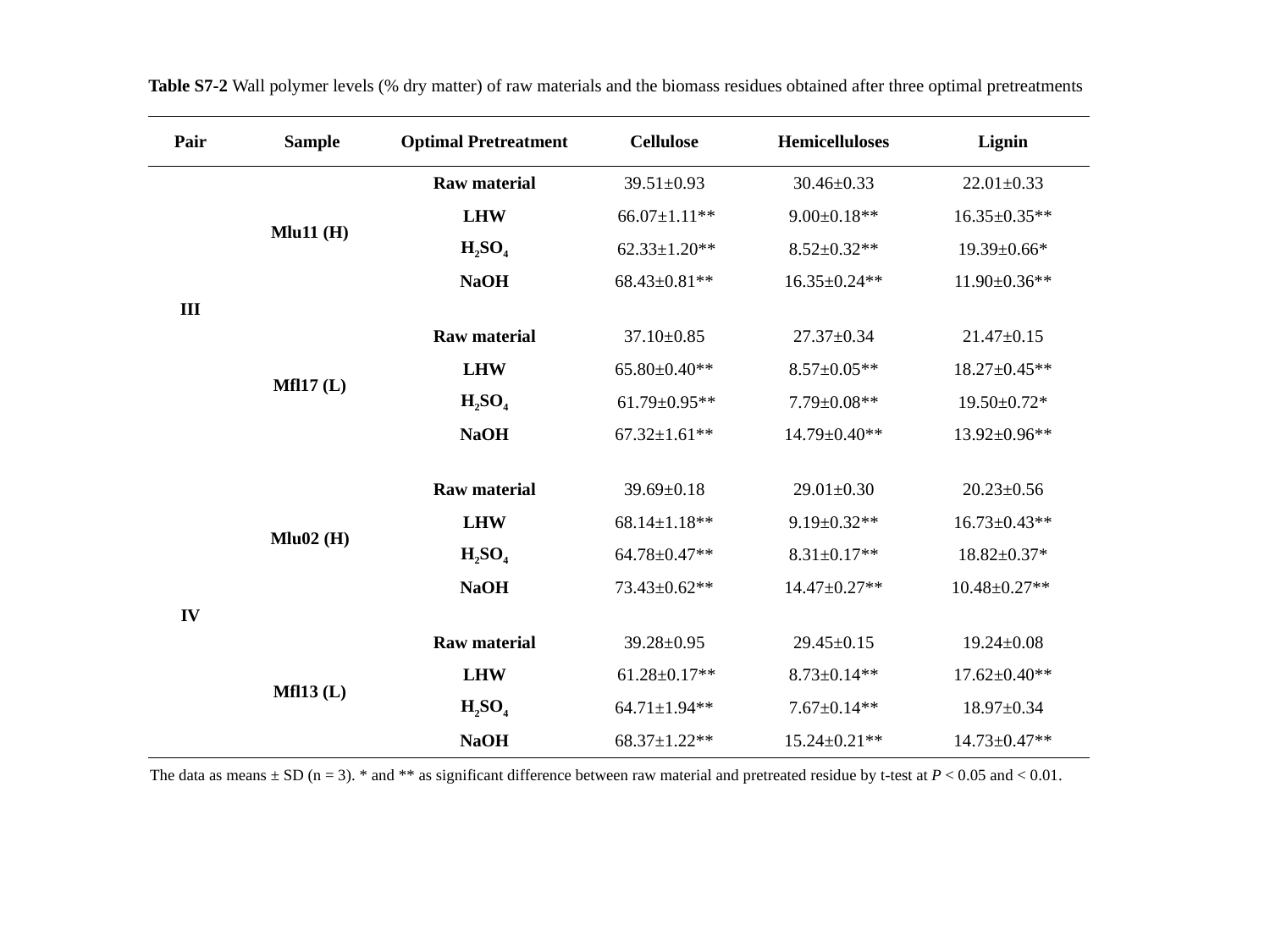

# Table S7-2 Wall polymer levels (% dry matter) of raw materials and the biomass residues obtained after three optimal pretreatments
| Pair | Sample | Optimal Pretreatment | Cellulose | Hemicelluloses | Lignin |
| --- | --- | --- | --- | --- | --- |
| III | Mlu11 (H) | Raw material | 39.51±0.93 | 30.46±0.33 | 22.01±0.33 |
| | | LHW | 66.07±1.11\*\* | 9.00±0.18\*\* | 16.35±0.35\*\* |
| | | H2SO4 | 62.33±1.20\*\* | 8.52±0.32\*\* | 19.39±0.66\* |
| | | NaOH | 68.43±0.81\*\* | 16.35±0.24\*\* | 11.90±0.36\*\* |
| | | | | | |
| | Mfl17 (L) | Raw material | 37.10±0.85 | 27.37±0.34 | 21.47±0.15 |
| | | LHW | 65.80±0.40\*\* | 8.57±0.05\*\* | 18.27±0.45\*\* |
| | | H2SO4 | 61.79±0.95\*\* | 7.79±0.08\*\* | 19.50±0.72\* |
| | | NaOH | 67.32±1.61\*\* | 14.79±0.40\*\* | 13.92±0.96\*\* |
| | | | | | |
| IV | Mlu02 (H) | Raw material | 39.69±0.18 | 29.01±0.30 | 20.23±0.56 |
| | | LHW | 68.14±1.18\*\* | 9.19±0.32\*\* | 16.73±0.43\*\* |
| | | H2SO4 | 64.78±0.47\*\* | 8.31±0.17\*\* | 18.82±0.37\* |
| | | NaOH | 73.43±0.62\*\* | 14.47±0.27\*\* | 10.48±0.27\*\* |
| | | | | | |
| | Mfl13 (L) | Raw material | 39.28±0.95 | 29.45±0.15 | 19.24±0.08 |
| | | LHW | 61.28±0.17\*\* | 8.73±0.14\*\* | 17.62±0.40\*\* |
| | | H2SO4 | 64.71±1.94\*\* | 7.67±0.14\*\* | 18.97±0.34 |
| | | NaOH | 68.37±1.22\*\* | 15.24±0.21\*\* | 14.73±0.47\*\* |
The data as means ± SD (n = 3). * and ** as significant difference between raw material and pretreated residue by t-test at P < 0.05 and < 0.01.

## Slide 10
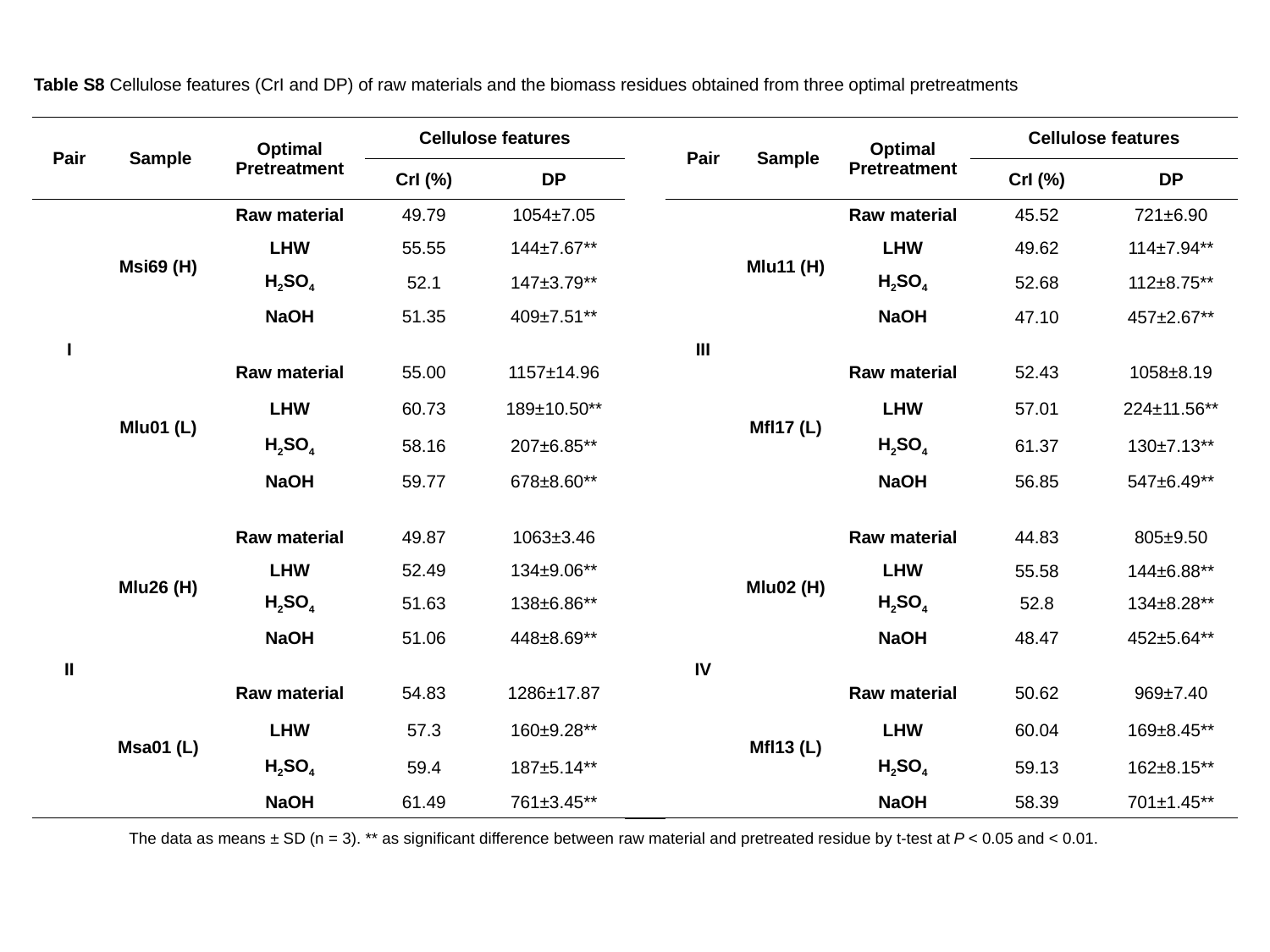

# Table S8 Cellulose features (CrI and DP) of raw materials and the biomass residues obtained from three optimal pretreatments
| Pair | Sample | Optimal Pretreatment | Cellulose features | | | Pair | Sample | Optimal Pretreatment | Cellulose features | |
| --- | --- | --- | --- | --- | --- | --- | --- | --- | --- | --- |
| | | | CrI (%) | DP | | | | | CrI (%) | DP |
| I | Msi69 (H) | Raw material | 49.79 | 1054±7.05 | | III | Mlu11 (H) | Raw material | 45.52 | 721±6.90 |
| | | LHW | 55.55 | 144±7.67\*\* | | | | LHW | 49.62 | 114±7.94\*\* |
| | | H2SO4 | 52.1 | 147±3.79\*\* | | | | H2SO4 | 52.68 | 112±8.75\*\* |
| | | NaOH | 51.35 | 409±7.51\*\* | | | | NaOH | 47.10 | 457±2.67\*\* |
| | | | | | | | | | | |
| | Mlu01 (L) | Raw material | 55.00 | 1157±14.96 | | | Mfl17 (L) | Raw material | 52.43 | 1058±8.19 |
| | | LHW | 60.73 | 189±10.50\*\* | | | | LHW | 57.01 | 224±11.56\*\* |
| | | H2SO4 | 58.16 | 207±6.85\*\* | | | | H2SO4 | 61.37 | 130±7.13\*\* |
| | | NaOH | 59.77 | 678±8.60\*\* | | | | NaOH | 56.85 | 547±6.49\*\* |
| | | | | | | | | | | |
| II | Mlu26 (H) | Raw material | 49.87 | 1063±3.46 | | IV | Mlu02 (H) | Raw material | 44.83 | 805±9.50 |
| | | LHW | 52.49 | 134±9.06\*\* | | | | LHW | 55.58 | 144±6.88\*\* |
| | | H2SO4 | 51.63 | 138±6.86\*\* | | | | H2SO4 | 52.8 | 134±8.28\*\* |
| | | NaOH | 51.06 | 448±8.69\*\* | | | | NaOH | 48.47 | 452±5.64\*\* |
| | | | | | | | | | | |
| | Msa01 (L) | Raw material | 54.83 | 1286±17.87 | | | Mfl13 (L) | Raw material | 50.62 | 969±7.40 |
| | | LHW | 57.3 | 160±9.28\*\* | | | | LHW | 60.04 | 169±8.45\*\* |
| | | H2SO4 | 59.4 | 187±5.14\*\* | | | | H2SO4 | 59.13 | 162±8.15\*\* |
| | | NaOH | 61.49 | 761±3.45\*\* | | | | NaOH | 58.39 | 701±1.45\*\* |
The data as means ± SD (n = 3). ** as significant difference between raw material and pretreated residue by t-test at P < 0.05 and < 0.01.

## Slide 11
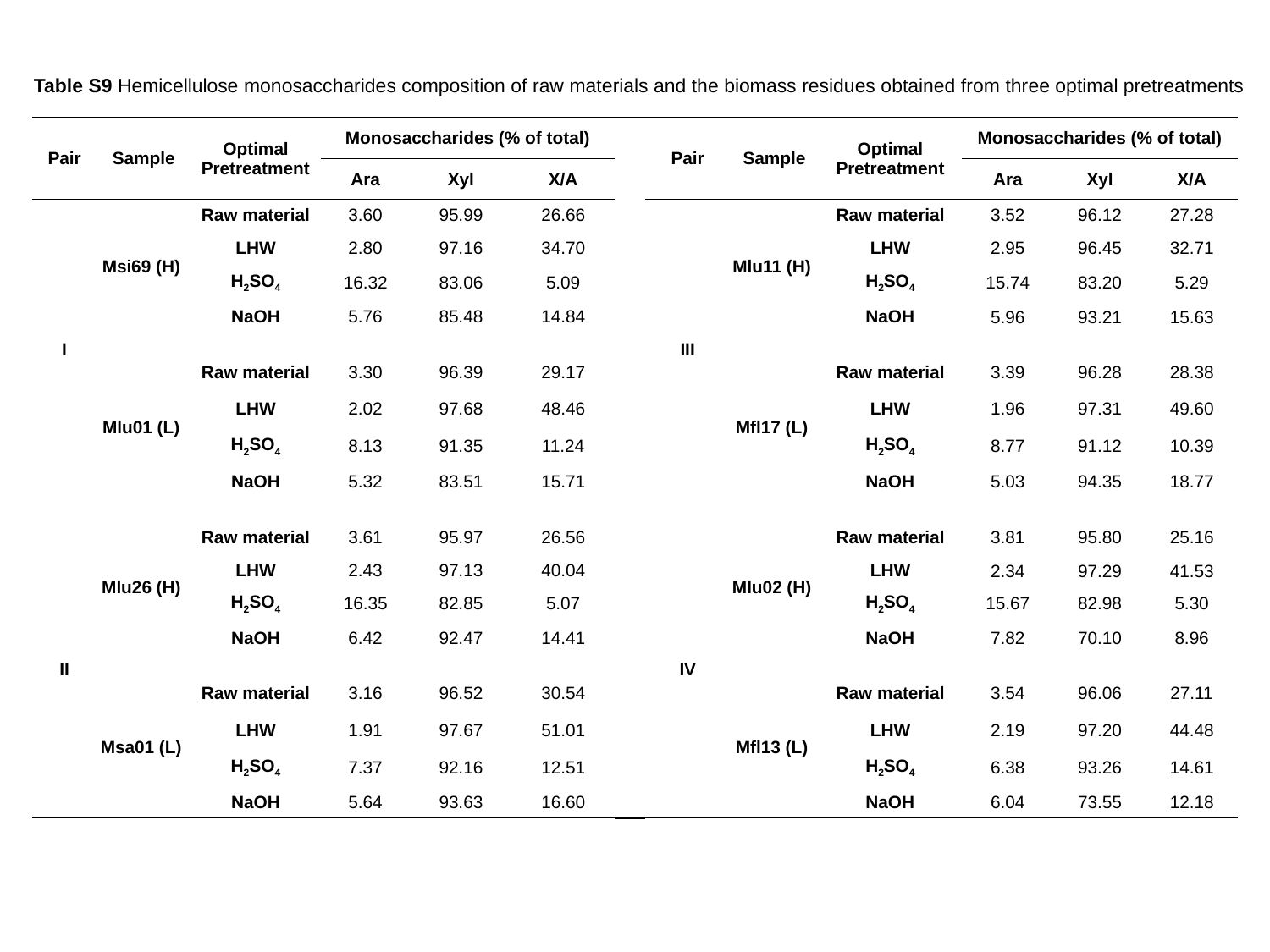

# Table S9 Hemicellulose monosaccharides composition of raw materials and the biomass residues obtained from three optimal pretreatments
| Pair | Sample | Optimal Pretreatment | Monosaccharides (% of total) | | | | Pair | Sample | Optimal Pretreatment | Monosaccharides (% of total) | | |
| --- | --- | --- | --- | --- | --- | --- | --- | --- | --- | --- | --- | --- |
| | | | Ara | Xyl | X/A | | | | | Ara | Xyl | X/A |
| I | Msi69 (H) | Raw material | 3.60 | 95.99 | 26.66 | | III | Mlu11 (H) | Raw material | 3.52 | 96.12 | 27.28 |
| | | LHW | 2.80 | 97.16 | 34.70 | | | | LHW | 2.95 | 96.45 | 32.71 |
| | | H2SO4 | 16.32 | 83.06 | 5.09 | | | | H2SO4 | 15.74 | 83.20 | 5.29 |
| | | NaOH | 5.76 | 85.48 | 14.84 | | | | NaOH | 5.96 | 93.21 | 15.63 |
| | | | | | | | | | | | | |
| | Mlu01 (L) | Raw material | 3.30 | 96.39 | 29.17 | | | Mfl17 (L) | Raw material | 3.39 | 96.28 | 28.38 |
| | | LHW | 2.02 | 97.68 | 48.46 | | | | LHW | 1.96 | 97.31 | 49.60 |
| | | H2SO4 | 8.13 | 91.35 | 11.24 | | | | H2SO4 | 8.77 | 91.12 | 10.39 |
| | | NaOH | 5.32 | 83.51 | 15.71 | | | | NaOH | 5.03 | 94.35 | 18.77 |
| | | | | | | | | | | | | |
| II | Mlu26 (H) | Raw material | 3.61 | 95.97 | 26.56 | | IV | Mlu02 (H) | Raw material | 3.81 | 95.80 | 25.16 |
| | | LHW | 2.43 | 97.13 | 40.04 | | | | LHW | 2.34 | 97.29 | 41.53 |
| | | H2SO4 | 16.35 | 82.85 | 5.07 | | | | H2SO4 | 15.67 | 82.98 | 5.30 |
| | | NaOH | 6.42 | 92.47 | 14.41 | | | | NaOH | 7.82 | 70.10 | 8.96 |
| | | | | | | | | | | | | |
| | Msa01 (L) | Raw material | 3.16 | 96.52 | 30.54 | | | Mfl13 (L) | Raw material | 3.54 | 96.06 | 27.11 |
| | | LHW | 1.91 | 97.67 | 51.01 | | | | LHW | 2.19 | 97.20 | 44.48 |
| | | H2SO4 | 7.37 | 92.16 | 12.51 | | | | H2SO4 | 6.38 | 93.26 | 14.61 |
| | | NaOH | 5.64 | 93.63 | 16.60 | | | | NaOH | 6.04 | 73.55 | 12.18 |

## Slide 12
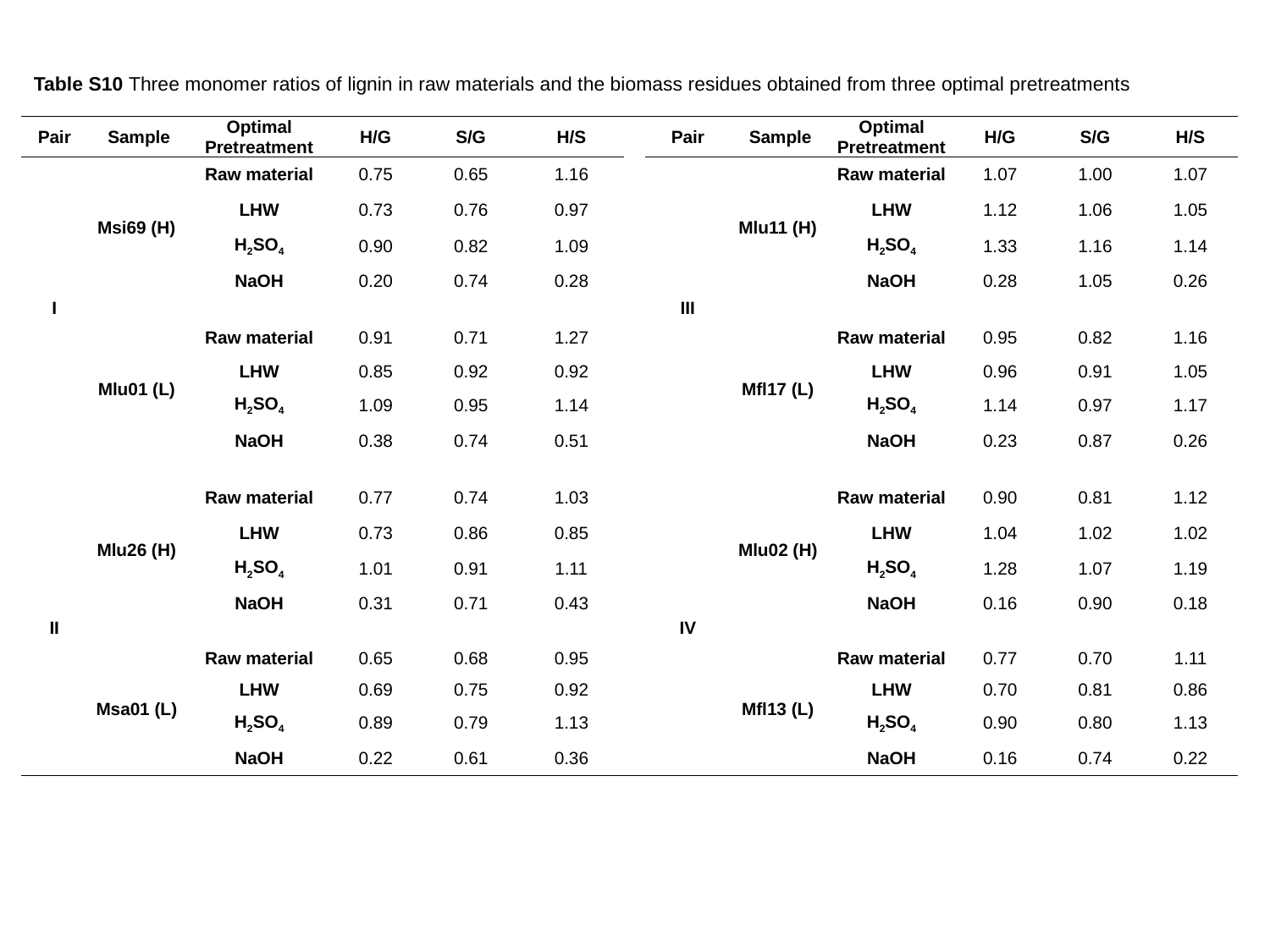

# Table S10 Three monomer ratios of lignin in raw materials and the biomass residues obtained from three optimal pretreatments
| Pair | Sample | Optimal Pretreatment | H/G | S/G | H/S | | Pair | Sample | Optimal Pretreatment | H/G | S/G | H/S |
| --- | --- | --- | --- | --- | --- | --- | --- | --- | --- | --- | --- | --- |
| I | Msi69 (H) | Raw material | 0.75 | 0.65 | 1.16 | | III | Mlu11 (H) | Raw material | 1.07 | 1.00 | 1.07 |
| | | LHW | 0.73 | 0.76 | 0.97 | | | | LHW | 1.12 | 1.06 | 1.05 |
| | | H2SO4 | 0.90 | 0.82 | 1.09 | | | | H2SO4 | 1.33 | 1.16 | 1.14 |
| | | NaOH | 0.20 | 0.74 | 0.28 | | | | NaOH | 0.28 | 1.05 | 0.26 |
| | | | | | | | | | | | | |
| | Mlu01 (L) | Raw material | 0.91 | 0.71 | 1.27 | | | Mfl17 (L) | Raw material | 0.95 | 0.82 | 1.16 |
| | | LHW | 0.85 | 0.92 | 0.92 | | | | LHW | 0.96 | 0.91 | 1.05 |
| | | H2SO4 | 1.09 | 0.95 | 1.14 | | | | H2SO4 | 1.14 | 0.97 | 1.17 |
| | | NaOH | 0.38 | 0.74 | 0.51 | | | | NaOH | 0.23 | 0.87 | 0.26 |
| | | | | | | | | | | | | |
| II | Mlu26 (H) | Raw material | 0.77 | 0.74 | 1.03 | | IV | Mlu02 (H) | Raw material | 0.90 | 0.81 | 1.12 |
| | | LHW | 0.73 | 0.86 | 0.85 | | | | LHW | 1.04 | 1.02 | 1.02 |
| | | H2SO4 | 1.01 | 0.91 | 1.11 | | | | H2SO4 | 1.28 | 1.07 | 1.19 |
| | | NaOH | 0.31 | 0.71 | 0.43 | | | | NaOH | 0.16 | 0.90 | 0.18 |
| | | | | | | | | | | | | |
| | Msa01 (L) | Raw material | 0.65 | 0.68 | 0.95 | | | Mfl13 (L) | Raw material | 0.77 | 0.70 | 1.11 |
| | | LHW | 0.69 | 0.75 | 0.92 | | | | LHW | 0.70 | 0.81 | 0.86 |
| | | H2SO4 | 0.89 | 0.79 | 1.13 | | | | H2SO4 | 0.90 | 0.80 | 1.13 |
| | | NaOH | 0.22 | 0.61 | 0.36 | | | | NaOH | 0.16 | 0.74 | 0.22 |

## Slide 13
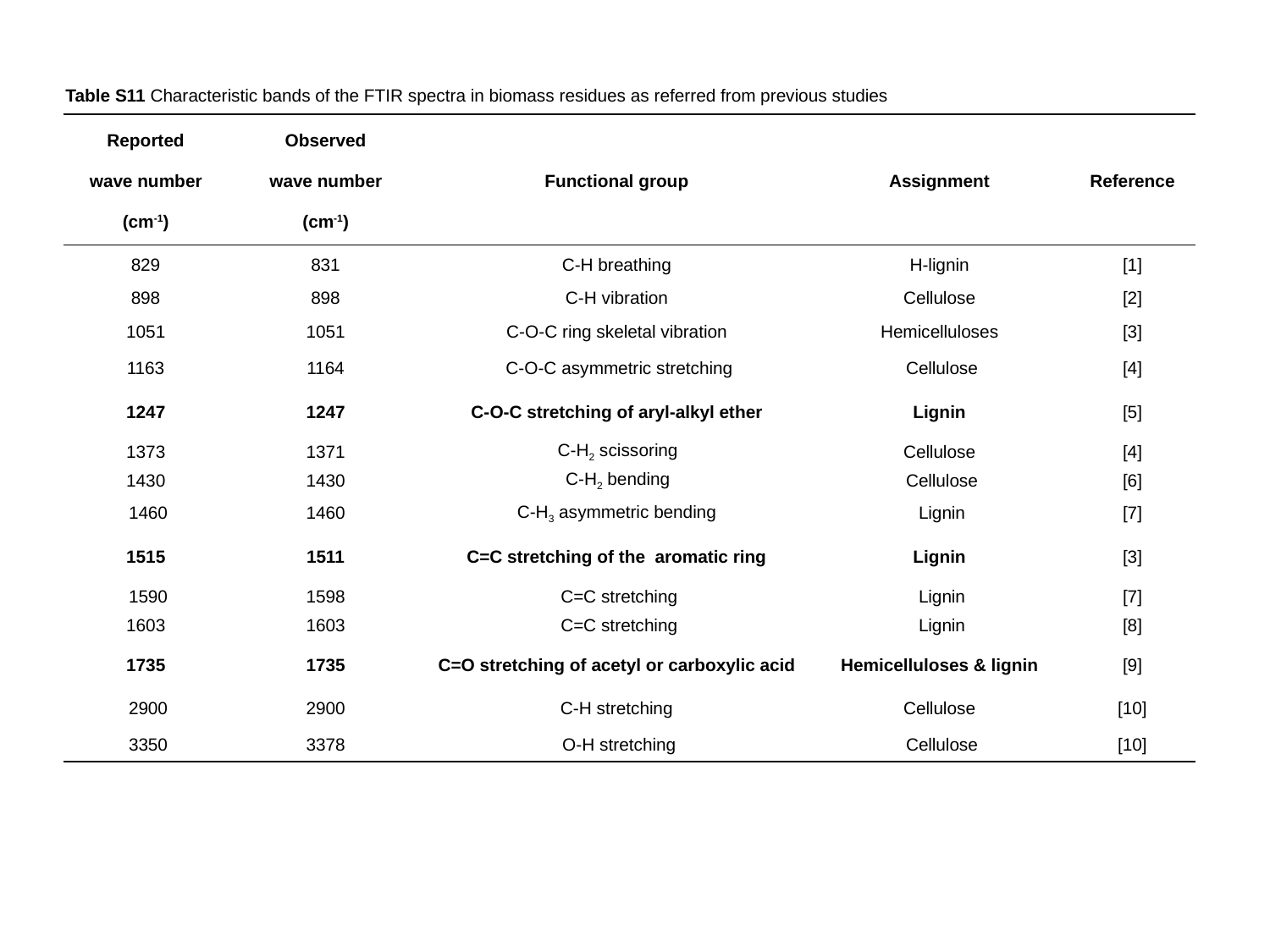

# Table S11 Characteristic bands of the FTIR spectra in biomass residues as referred from previous studies
| Reported wave number (cm-1) | Observed wave number (cm-1) | Functional group | Assignment | Reference |
| --- | --- | --- | --- | --- |
| 829 | 831 | C-H breathing | H-lignin | [1] |
| 898 | 898 | C-H vibration | Cellulose | [2] |
| 1051 | 1051 | C-O-C ring skeletal vibration | Hemicelluloses | [3] |
| 1163 | 1164 | C-O-C asymmetric stretching | Cellulose | [4] |
| 1247 | 1247 | C-O-C stretching of aryl-alkyl ether | Lignin | [5] |
| 1373 | 1371 | C-H2 scissoring | Cellulose | [4] |
| 1430 | 1430 | C-H2 bending | Cellulose | [6] |
| 1460 | 1460 | C-H3 asymmetric bending | Lignin | [7] |
| 1515 | 1511 | C=C stretching of the aromatic ring | Lignin | [3] |
| 1590 | 1598 | C=C stretching | Lignin | [7] |
| 1603 | 1603 | C=C stretching | Lignin | [8] |
| 1735 | 1735 | C=O stretching of acetyl or carboxylic acid | Hemicelluloses & lignin | [9] |
| 2900 | 2900 | C-H stretching | Cellulose | [10] |
| 3350 | 3378 | O-H stretching | Cellulose | [10] |

## Slide 14
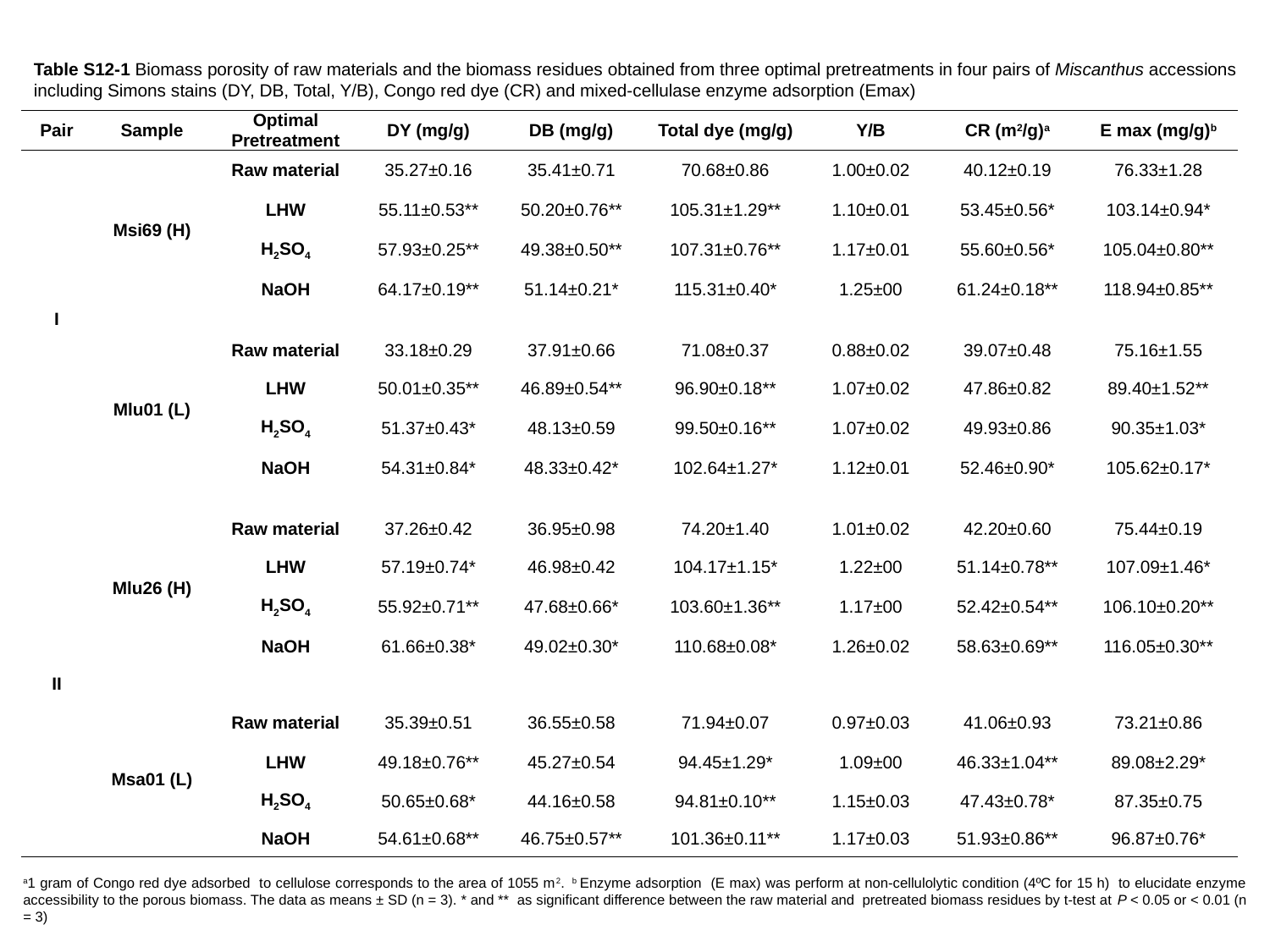

# Table S12-1 Biomass porosity of raw materials and the biomass residues obtained from three optimal pretreatments in four pairs of Miscanthus accessions including Simons stains (DY, DB, Total, Y/B), Congo red dye (CR) and mixed-cellulase enzyme adsorption (Emax)
| Pair | Sample | Optimal Pretreatment | DY (mg/g) | DB (mg/g) | Total dye (mg/g) | Y/B | CR (m2/g)a | E max (mg/g)b |
| --- | --- | --- | --- | --- | --- | --- | --- | --- |
| I | Msi69 (H) | Raw material | 35.27±0.16 | 35.41±0.71 | 70.68±0.86 | 1.00±0.02 | 40.12±0.19 | 76.33±1.28 |
| | | LHW | 55.11±0.53\*\* | 50.20±0.76\*\* | 105.31±1.29\*\* | 1.10±0.01 | 53.45±0.56\* | 103.14±0.94\* |
| | | H2SO4 | 57.93±0.25\*\* | 49.38±0.50\*\* | 107.31±0.76\*\* | 1.17±0.01 | 55.60±0.56\* | 105.04±0.80\*\* |
| | | NaOH | 64.17±0.19\*\* | 51.14±0.21\* | 115.31±0.40\* | 1.25±00 | 61.24±0.18\*\* | 118.94±0.85\*\* |
| | | | | | | | | |
| | Mlu01 (L) | Raw material | 33.18±0.29 | 37.91±0.66 | 71.08±0.37 | 0.88±0.02 | 39.07±0.48 | 75.16±1.55 |
| | | LHW | 50.01±0.35\*\* | 46.89±0.54\*\* | 96.90±0.18\*\* | 1.07±0.02 | 47.86±0.82 | 89.40±1.52\*\* |
| | | H2SO4 | 51.37±0.43\* | 48.13±0.59 | 99.50±0.16\*\* | 1.07±0.02 | 49.93±0.86 | 90.35±1.03\* |
| | | NaOH | 54.31±0.84\* | 48.33±0.42\* | 102.64±1.27\* | 1.12±0.01 | 52.46±0.90\* | 105.62±0.17\* |
| | | | | | | | | |
| II | Mlu26 (H) | Raw material | 37.26±0.42 | 36.95±0.98 | 74.20±1.40 | 1.01±0.02 | 42.20±0.60 | 75.44±0.19 |
| | | LHW | 57.19±0.74\* | 46.98±0.42 | 104.17±1.15\* | 1.22±00 | 51.14±0.78\*\* | 107.09±1.46\* |
| | | H2SO4 | 55.92±0.71\*\* | 47.68±0.66\* | 103.60±1.36\*\* | 1.17±00 | 52.42±0.54\*\* | 106.10±0.20\*\* |
| | | NaOH | 61.66±0.38\* | 49.02±0.30\* | 110.68±0.08\* | 1.26±0.02 | 58.63±0.69\*\* | 116.05±0.30\*\* |
| | | | | | | | | |
| | Msa01 (L) | Raw material | 35.39±0.51 | 36.55±0.58 | 71.94±0.07 | 0.97±0.03 | 41.06±0.93 | 73.21±0.86 |
| | | LHW | 49.18±0.76\*\* | 45.27±0.54 | 94.45±1.29\* | 1.09±00 | 46.33±1.04\*\* | 89.08±2.29\* |
| | | H2SO4 | 50.65±0.68\* | 44.16±0.58 | 94.81±0.10\*\* | 1.15±0.03 | 47.43±0.78\* | 87.35±0.75 |
| | | NaOH | 54.61±0.68\*\* | 46.75±0.57\*\* | 101.36±0.11\*\* | 1.17±0.03 | 51.93±0.86\*\* | 96.87±0.76\* |
a1 gram of Congo red dye adsorbed to cellulose corresponds to the area of 1055 m2. b Enzyme adsorption (E max) was perform at non-cellulolytic condition (4ºC for 15 h) to elucidate enzyme accessibility to the porous biomass. The data as means ± SD (n = 3). * and ** as significant difference between the raw material and pretreated biomass residues by t-test at P < 0.05 or < 0.01 (n = 3)

## Slide 15
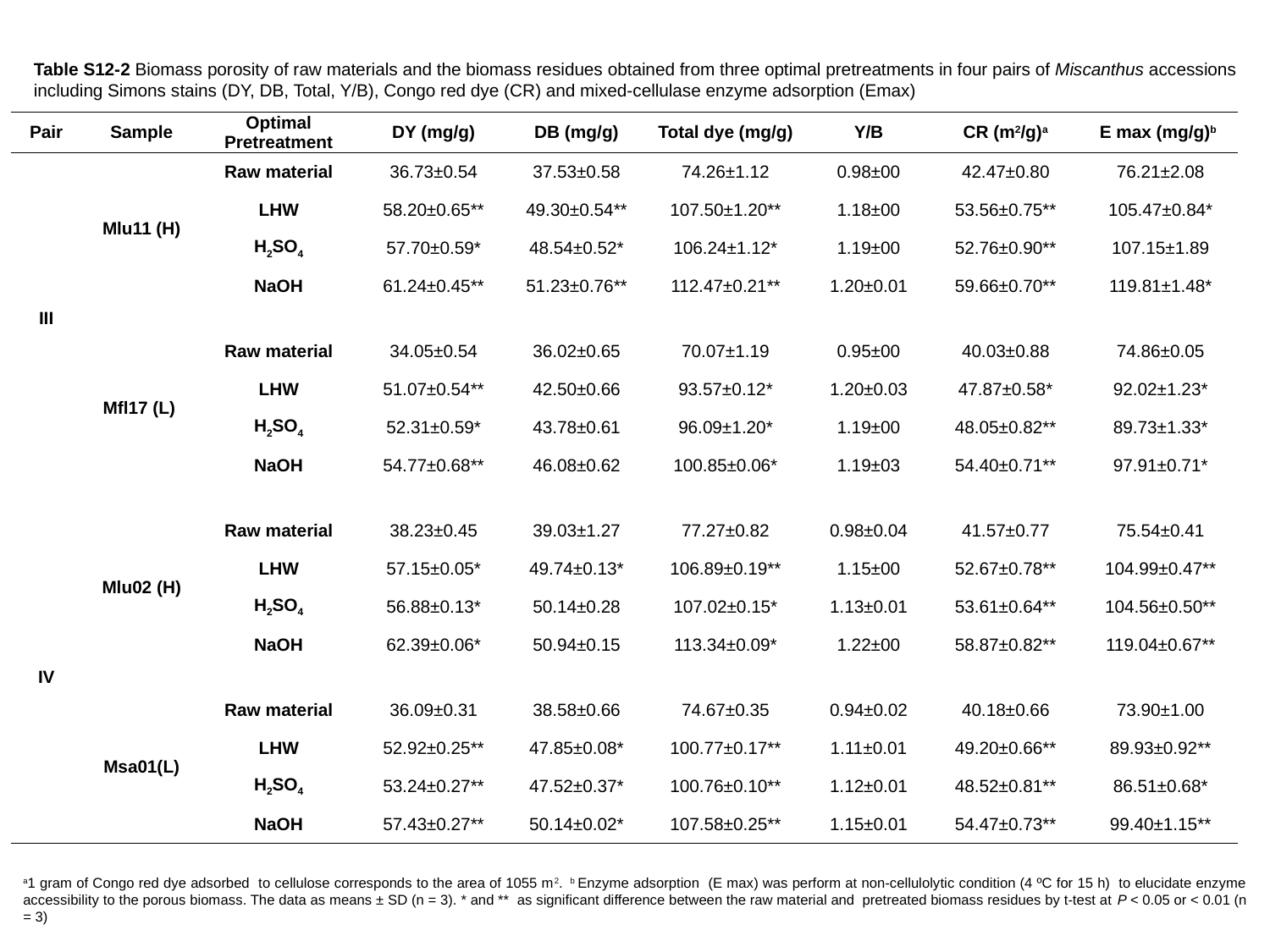

# Table S12-2 Biomass porosity of raw materials and the biomass residues obtained from three optimal pretreatments in four pairs of Miscanthus accessions including Simons stains (DY, DB, Total, Y/B), Congo red dye (CR) and mixed-cellulase enzyme adsorption (Emax)
| Pair | Sample | Optimal Pretreatment | DY (mg/g) | DB (mg/g) | Total dye (mg/g) | Y/B | CR (m2/g)a | E max (mg/g)b |
| --- | --- | --- | --- | --- | --- | --- | --- | --- |
| III | Mlu11 (H) | Raw material | 36.73±0.54 | 37.53±0.58 | 74.26±1.12 | 0.98±00 | 42.47±0.80 | 76.21±2.08 |
| | | LHW | 58.20±0.65\*\* | 49.30±0.54\*\* | 107.50±1.20\*\* | 1.18±00 | 53.56±0.75\*\* | 105.47±0.84\* |
| | | H2SO4 | 57.70±0.59\* | 48.54±0.52\* | 106.24±1.12\* | 1.19±00 | 52.76±0.90\*\* | 107.15±1.89 |
| | | NaOH | 61.24±0.45\*\* | 51.23±0.76\*\* | 112.47±0.21\*\* | 1.20±0.01 | 59.66±0.70\*\* | 119.81±1.48\* |
| | | | | | | | | |
| | Mfl17 (L) | Raw material | 34.05±0.54 | 36.02±0.65 | 70.07±1.19 | 0.95±00 | 40.03±0.88 | 74.86±0.05 |
| | | LHW | 51.07±0.54\*\* | 42.50±0.66 | 93.57±0.12\* | 1.20±0.03 | 47.87±0.58\* | 92.02±1.23\* |
| | | H2SO4 | 52.31±0.59\* | 43.78±0.61 | 96.09±1.20\* | 1.19±00 | 48.05±0.82\*\* | 89.73±1.33\* |
| | | NaOH | 54.77±0.68\*\* | 46.08±0.62 | 100.85±0.06\* | 1.19±03 | 54.40±0.71\*\* | 97.91±0.71\* |
| | | | | | | | | |
| IV | Mlu02 (H) | Raw material | 38.23±0.45 | 39.03±1.27 | 77.27±0.82 | 0.98±0.04 | 41.57±0.77 | 75.54±0.41 |
| | | LHW | 57.15±0.05\* | 49.74±0.13\* | 106.89±0.19\*\* | 1.15±00 | 52.67±0.78\*\* | 104.99±0.47\*\* |
| | | H2SO4 | 56.88±0.13\* | 50.14±0.28 | 107.02±0.15\* | 1.13±0.01 | 53.61±0.64\*\* | 104.56±0.50\*\* |
| | | NaOH | 62.39±0.06\* | 50.94±0.15 | 113.34±0.09\* | 1.22±00 | 58.87±0.82\*\* | 119.04±0.67\*\* |
| | | | | | | | | |
| | Msa01(L) | Raw material | 36.09±0.31 | 38.58±0.66 | 74.67±0.35 | 0.94±0.02 | 40.18±0.66 | 73.90±1.00 |
| | | LHW | 52.92±0.25\*\* | 47.85±0.08\* | 100.77±0.17\*\* | 1.11±0.01 | 49.20±0.66\*\* | 89.93±0.92\*\* |
| | | H2SO4 | 53.24±0.27\*\* | 47.52±0.37\* | 100.76±0.10\*\* | 1.12±0.01 | 48.52±0.81\*\* | 86.51±0.68\* |
| | | NaOH | 57.43±0.27\*\* | 50.14±0.02\* | 107.58±0.25\*\* | 1.15±0.01 | 54.47±0.73\*\* | 99.40±1.15\*\* |
a1 gram of Congo red dye adsorbed to cellulose corresponds to the area of 1055 m2. b Enzyme adsorption (E max) was perform at non-cellulolytic condition (4 ºC for 15 h) to elucidate enzyme accessibility to the porous biomass. The data as means ± SD (n = 3). * and ** as significant difference between the raw material and pretreated biomass residues by t-test at P < 0.05 or < 0.01 (n = 3)

## Slide 16
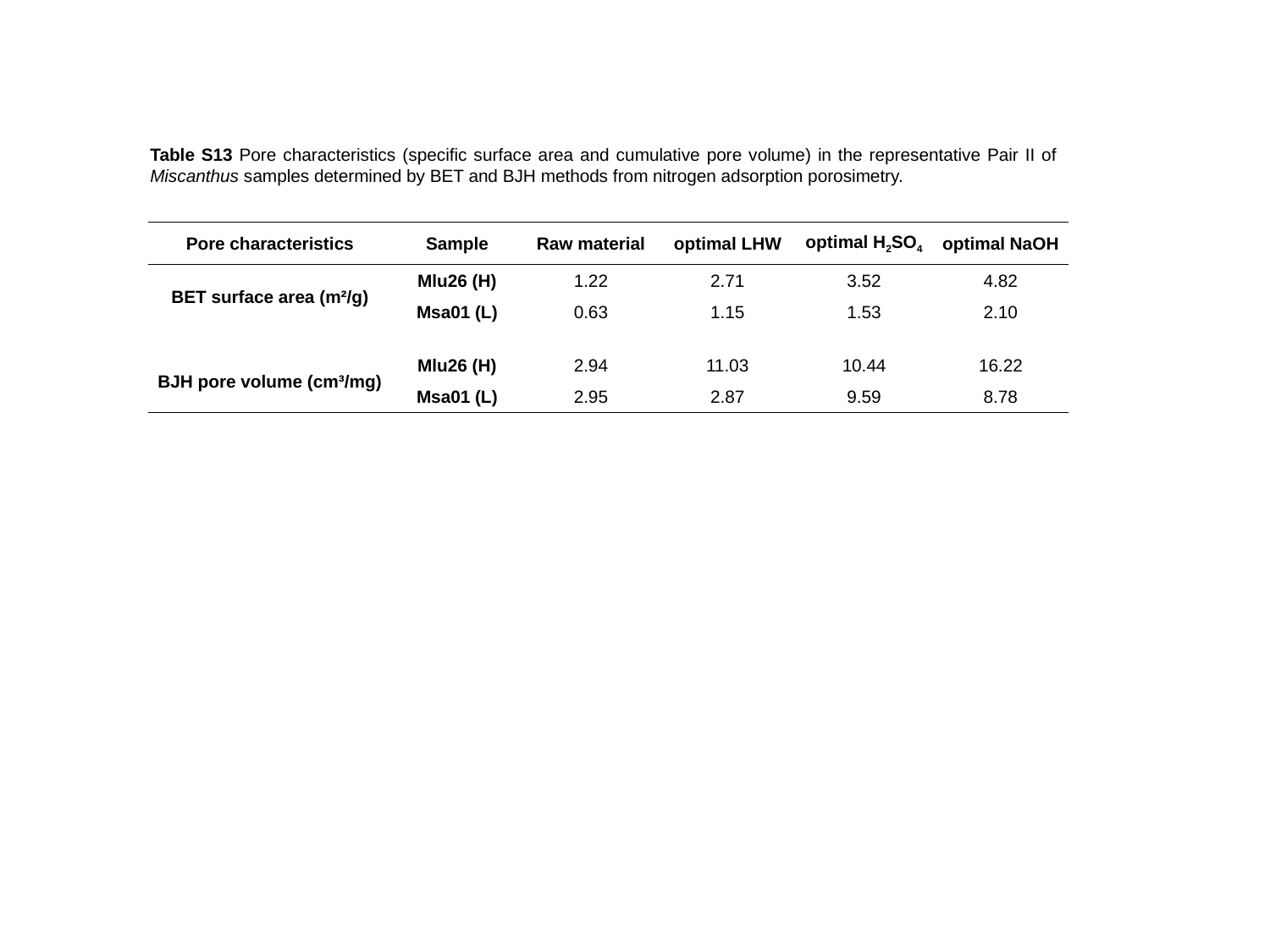

Table S13 Pore characteristics (specific surface area and cumulative pore volume) in the representative Pair II of Miscanthus samples determined by BET and BJH methods from nitrogen adsorption porosimetry.
| Pore characteristics | Sample | Raw material | optimal LHW | optimal H2SO4 | optimal NaOH |
| --- | --- | --- | --- | --- | --- |
| BET surface area (m²/g) | Mlu26 (H) | 1.22 | 2.71 | 3.52 | 4.82 |
| | Msa01 (L) | 0.63 | 1.15 | 1.53 | 2.10 |
| | | | | | |
| BJH pore volume (cm³/mg) | Mlu26 (H) | 2.94 | 11.03 | 10.44 | 16.22 |
| | Msa01 (L) | 2.95 | 2.87 | 9.59 | 8.78 |

## Slide 17
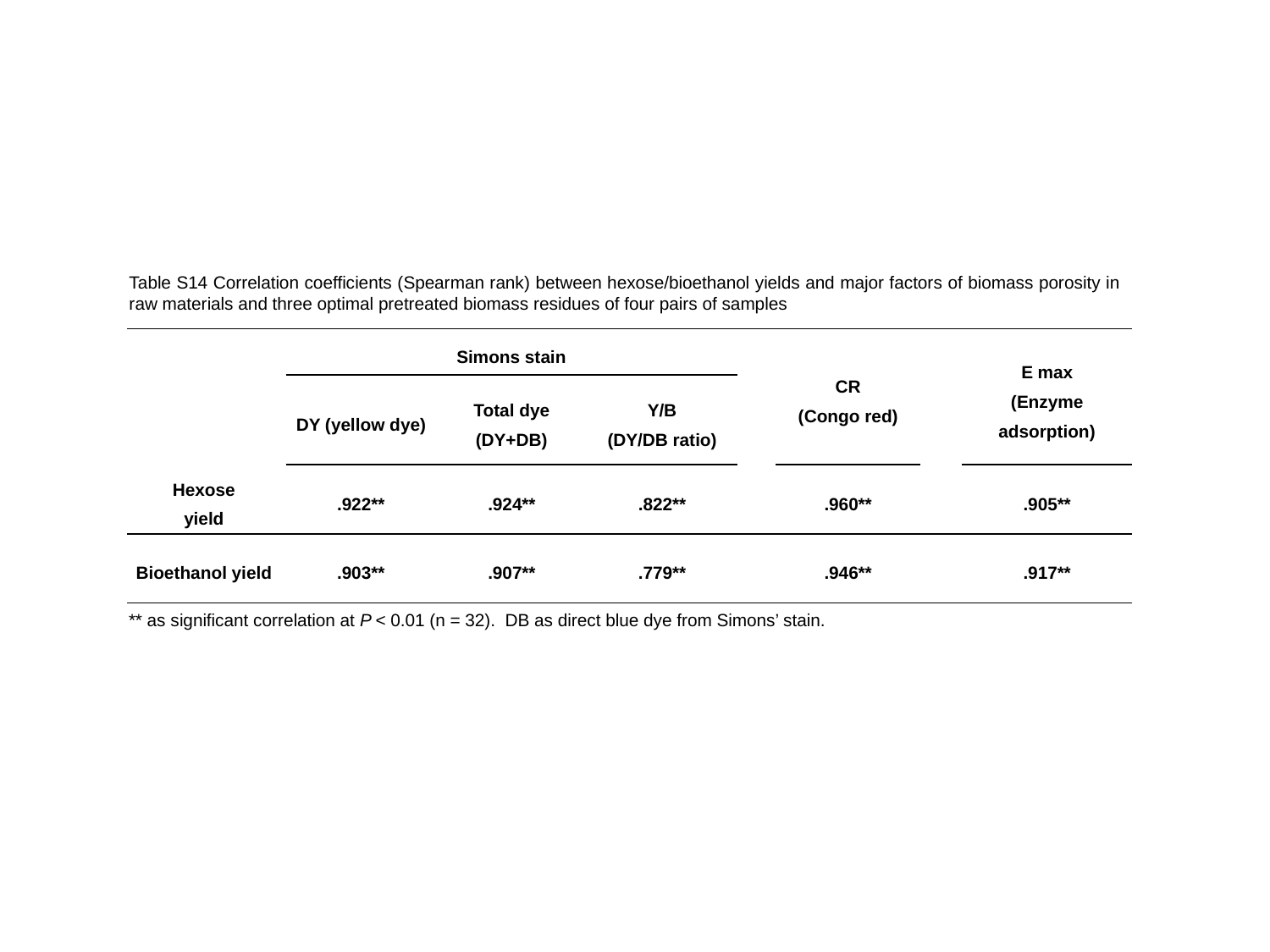

Table S14 Correlation coefficients (Spearman rank) between hexose/bioethanol yields and major factors of biomass porosity in raw materials and three optimal pretreated biomass residues of four pairs of samples
| | Simons stain | | | | CR (Congo red) | | E max (Enzyme adsorption) |
| --- | --- | --- | --- | --- | --- | --- | --- |
| | DY (yellow dye) | Total dye (DY+DB) | Y/B (DY/DB ratio) | | | | |
| Hexose yield | .922\*\* | .924\*\* | .822\*\* | | .960\*\* | | .905\*\* |
| Bioethanol yield | .903\*\* | .907\*\* | .779\*\* | | .946\*\* | | .917\*\* |
** as significant correlation at P < 0.01 (n = 32). DB as direct blue dye from Simons’ stain.

## Slide 18
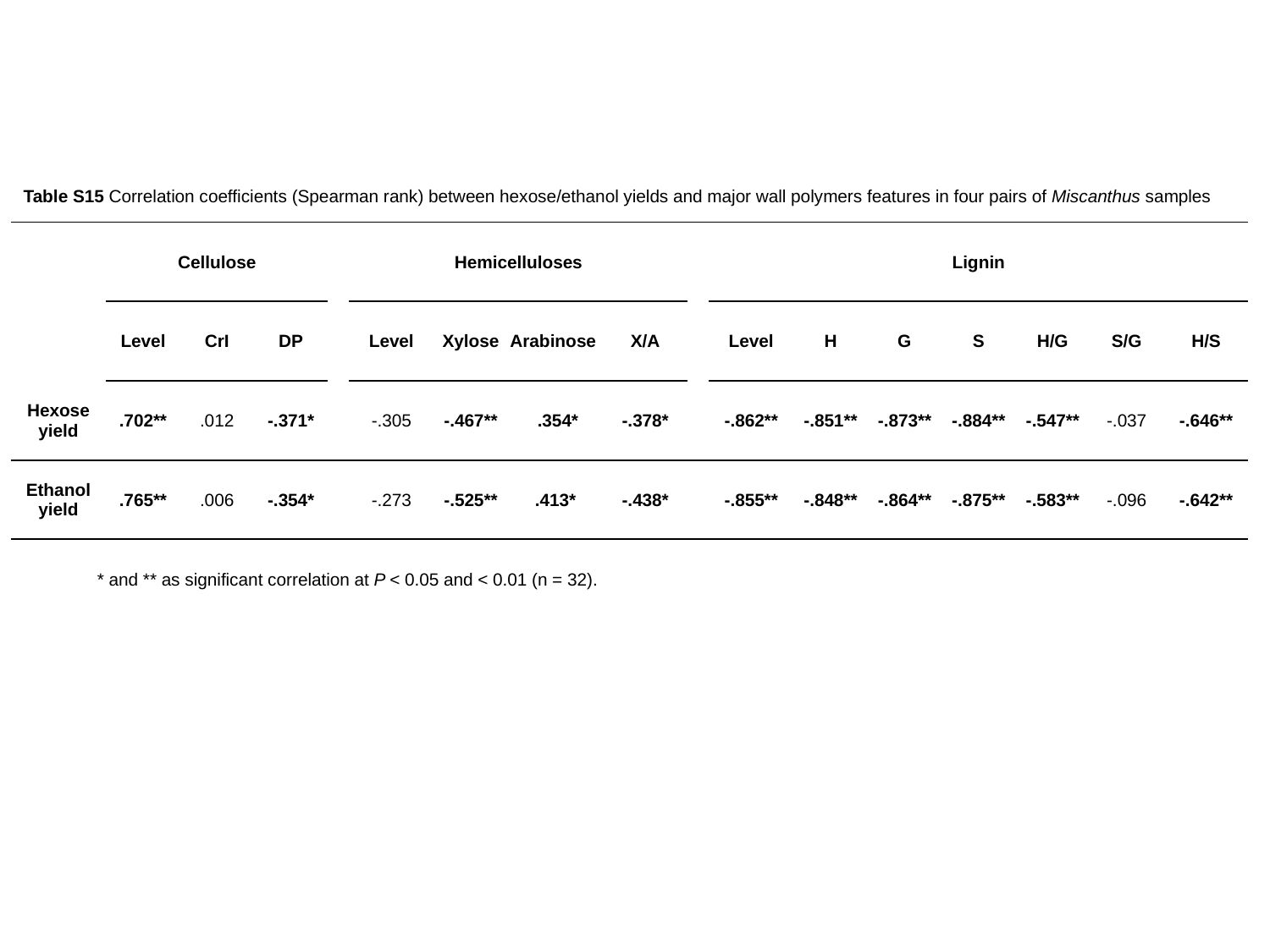

# Table S15 Correlation coefficients (Spearman rank) between hexose/ethanol yields and major wall polymers features in four pairs of Miscanthus samples
| | Cellulose | | | | Hemicelluloses | | | | | Lignin | | | | | | |
| --- | --- | --- | --- | --- | --- | --- | --- | --- | --- | --- | --- | --- | --- | --- | --- | --- |
| | Level | CrI | DP | | Level | Xylose | Arabinose | X/A | | Level | H | G | S | H/G | S/G | H/S |
| Hexose yield | .702\*\* | .012 | -.371\* | | -.305 | -.467\*\* | .354\* | -.378\* | | -.862\*\* | -.851\*\* | -.873\*\* | -.884\*\* | -.547\*\* | -.037 | -.646\*\* |
| Ethanol yield | .765\*\* | .006 | -.354\* | | -.273 | -.525\*\* | .413\* | -.438\* | | -.855\*\* | -.848\*\* | -.864\*\* | -.875\*\* | -.583\*\* | -.096 | -.642\*\* |
* and ** as significant correlation at P < 0.05 and < 0.01 (n = 32).

## Slide 19
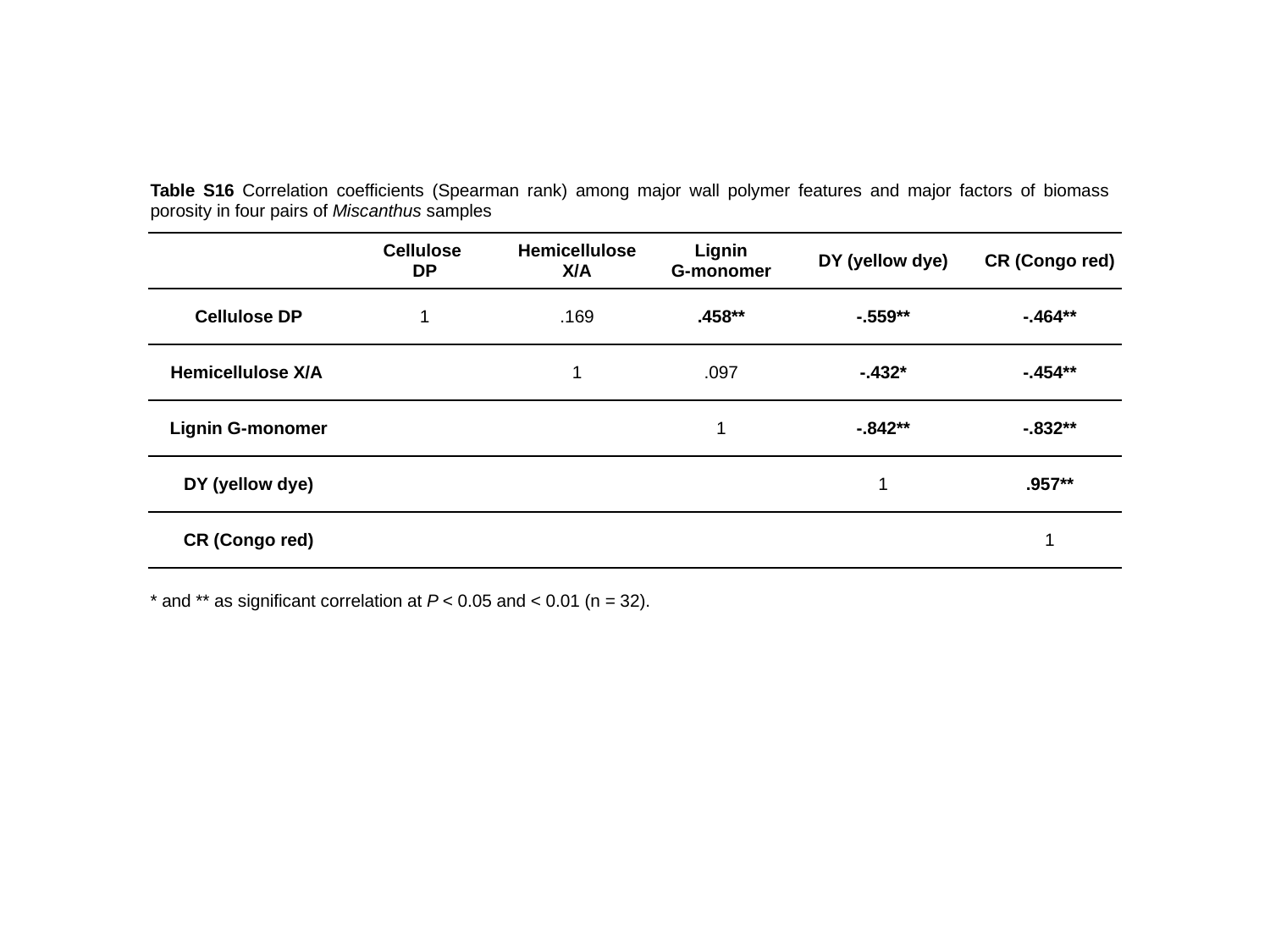

# Table S16 Correlation coefficients (Spearman rank) among major wall polymer features and major factors of biomass porosity in four pairs of Miscanthus samples
| | Cellulose DP | Hemicellulose X/A | Lignin G-monomer | DY (yellow dye) | CR (Congo red) |
| --- | --- | --- | --- | --- | --- |
| Cellulose DP | 1 | .169 | .458\*\* | -.559\*\* | -.464\*\* |
| Hemicellulose X/A | | 1 | .097 | -.432\* | -.454\*\* |
| Lignin G-monomer | | | 1 | -.842\*\* | -.832\*\* |
| DY (yellow dye) | | | | 1 | .957\*\* |
| CR (Congo red) | | | | | 1 |
* and ** as significant correlation at P < 0.05 and < 0.01 (n = 32).

## Slide 20
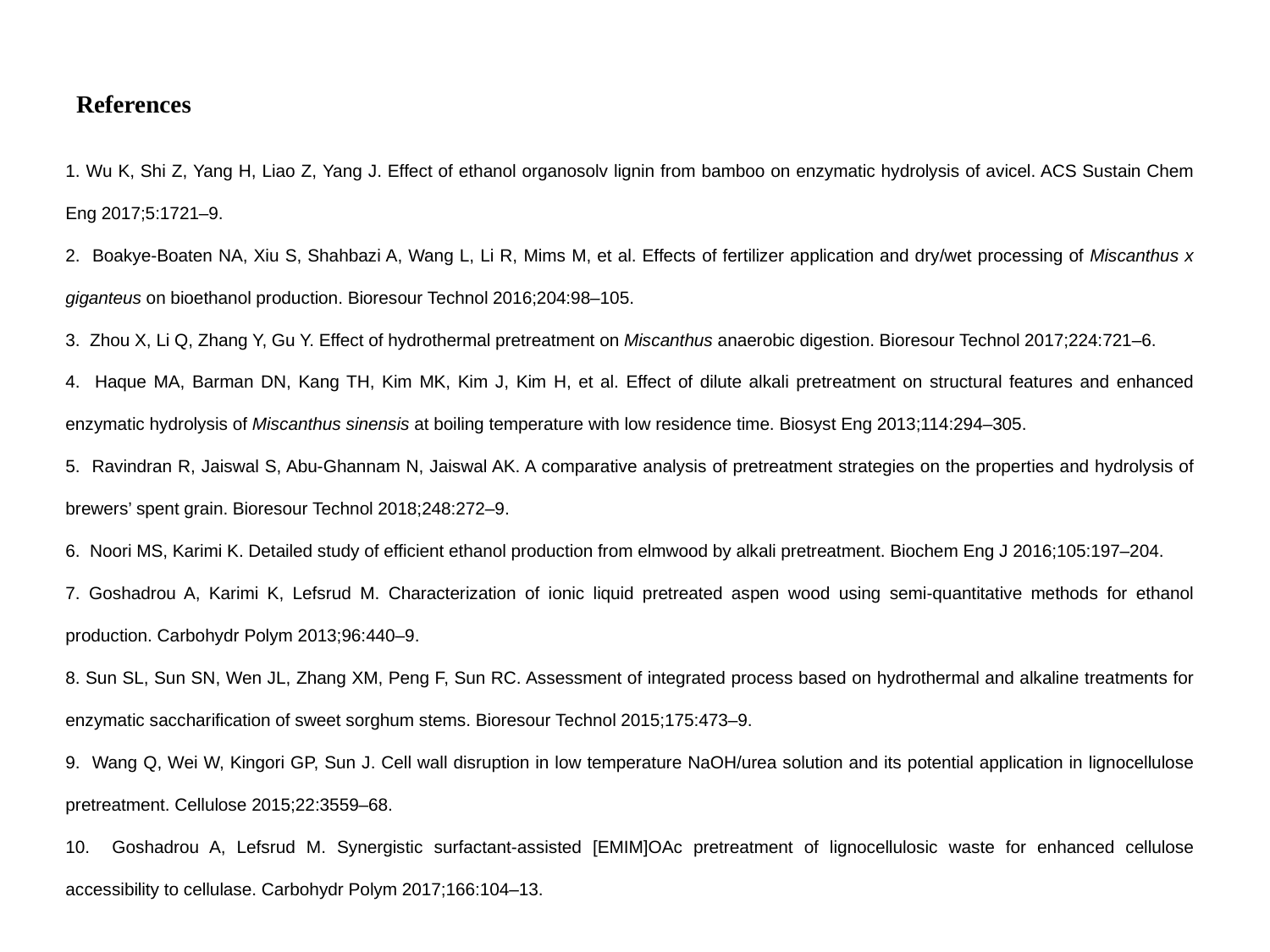

References
1. Wu K, Shi Z, Yang H, Liao Z, Yang J. Effect of ethanol organosolv lignin from bamboo on enzymatic hydrolysis of avicel. ACS Sustain Chem Eng 2017;5:1721–9.
2. Boakye-Boaten NA, Xiu S, Shahbazi A, Wang L, Li R, Mims M, et al. Effects of fertilizer application and dry/wet processing of Miscanthus x giganteus on bioethanol production. Bioresour Technol 2016;204:98–105.
3. Zhou X, Li Q, Zhang Y, Gu Y. Effect of hydrothermal pretreatment on Miscanthus anaerobic digestion. Bioresour Technol 2017;224:721–6.
4. Haque MA, Barman DN, Kang TH, Kim MK, Kim J, Kim H, et al. Effect of dilute alkali pretreatment on structural features and enhanced enzymatic hydrolysis of Miscanthus sinensis at boiling temperature with low residence time. Biosyst Eng 2013;114:294–305.
5. Ravindran R, Jaiswal S, Abu-Ghannam N, Jaiswal AK. A comparative analysis of pretreatment strategies on the properties and hydrolysis of brewers’ spent grain. Bioresour Technol 2018;248:272–9.
6. Noori MS, Karimi K. Detailed study of efficient ethanol production from elmwood by alkali pretreatment. Biochem Eng J 2016;105:197–204.
7. Goshadrou A, Karimi K, Lefsrud M. Characterization of ionic liquid pretreated aspen wood using semi-quantitative methods for ethanol production. Carbohydr Polym 2013;96:440–9.
8. Sun SL, Sun SN, Wen JL, Zhang XM, Peng F, Sun RC. Assessment of integrated process based on hydrothermal and alkaline treatments for enzymatic saccharification of sweet sorghum stems. Bioresour Technol 2015;175:473–9.
9. Wang Q, Wei W, Kingori GP, Sun J. Cell wall disruption in low temperature NaOH/urea solution and its potential application in lignocellulose pretreatment. Cellulose 2015;22:3559–68.
10. Goshadrou A, Lefsrud M. Synergistic surfactant-assisted [EMIM]OAc pretreatment of lignocellulosic waste for enhanced cellulose accessibility to cellulase. Carbohydr Polym 2017;166:104–13.
